# The Open-source Camera Trap for Organism Presence and Underwater Surveillance (OCTOPUS)

**DOI:** 10.1016/j.ohx.2023.e00394

**Published:** 2023-01-13

**Authors:** Jefferson W. Humbert, Kirt L. Onthank, Kresimir Williams

**Affiliations:** a Department of Biological Sciences, Walla Walla University, College Place, WA, United States; b National Marine Fisheries Service, Alaska Fisheries Science Center , Resource Assessment and Conservation Engineering Division, 7600 Sand Point Way NE, Seattle, WA 98115, United States

**Keywords:** Citizen science, Open-source technology, Trigger camera, Camera trap, Benthic ecology, Population ecology

## Abstract

This Open-source Camera Trap for Organism Presence and Underwater Surveillance (OCTOPUS) was designed to operate as a motion activated camera trap, deployable at depths of up to 800 ft for ∼72 h deployments. The core components of the OCTOPUS are built off a Raspberry Pi 3B+ with a custom PCB hat which operates a strobe lighting system and a high definition Arducam camera. When an appropriate threshold of motion is detected, the OCTOPUS captures a high-definition image of the subject. Field trials for this system demonstrated its use for cryptic benthic organisms, specifically small octopus (*Octopus rubescens*). The OCTOPUS collected data on several species allowing the observation and quantification of interspecific and conspecific interactions. This system unlocks the potential of autonomous underwater data collection for a wide range of applications, from species specific observations to large scale ecological assessments.

## Graphical abstract

**Figure:**
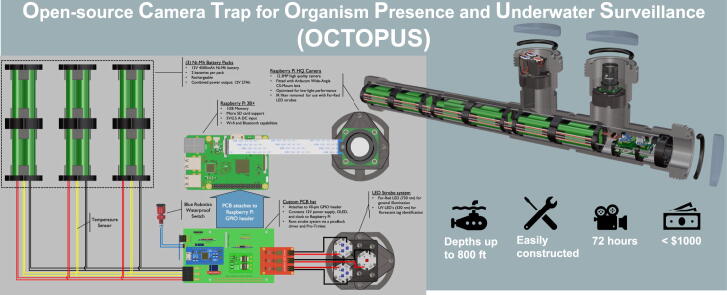

**Specifications table**

**Table:** 

Hardware name	The Open Underwater Trigger Camera
Subject area	Biological Sciences, Environmental Sciences, Open Source Alternatives to Existing Infrastructure
Hardware type	Field measurements and sensors, Imaging tools, Electrical engineering and computer science
Closest commercial analog	https://www.spotx.com.au/underwater-camera-trap/underwater-camera-trap
Open Source License	Creative Commons Attribution-ShareAlike license
Cost of Hardware	∼$900–1000
Source File Repository	Software, Housing and PCB: Zenodo (https://zenodo.org/record/7039012)
OSHWA Certification UID	US002113

## 1 Hardware in context

The lack of affordable long-term aquatic monitoring systems has limited our understanding of aquatic environments. Non-invasive aquatic ecosystem surveillance provides valuable data essential to our thorough understanding of ecology and biodiversity in marine and freshwater environments. Camera traps have become established in terrestrial systems as a key tool in research, leading to determination of species richness, distribution and abundance [1]. Terrestrial mammals have been estimated to account for 95 % of camera trapping research [2], indicating a severe lack of application in aquatic systems primarily due to the many challenges faced by underwater camera trap systems. Aside from the difficulty of water- and pressure-proofing the electronics, the biggest constraint in aquatic use of camera traps is the attenuation of infrared (IR) light underwater, which is essential to the triggering mechanisms of terrestrial systems. Often easily affordable and attainable action cameras such as GoPro’s are placed in waterproof housings and used for underwater visual studies and used as time-lapse camera systems in which a image is captured at regular intervals. However these systems face limitations in battery capacity and often results in partial or missing data since they rely on a timed trigger instead of a motion trigger. Many different vision-based systems have been developed in prior years [3], [4], [5], [6], [7], but are often limited by constraints such as deployment duration, photo quality, depth rating, motion sensitivity, etc. The proposed system attempts to address the limitations to these systems while remaining affordable and open-source. While this system cannot attain durations that are possible for terrestrial systems, which can often operate for months without battery recharging, the multi-day operation of this system represents a large improvement for underwater applications over other low-cost underwater camera options. This system uses a motion-trigger function which detects motion using computer vision algorithms implemented by the OpenCV library to capture images of organisms. A motion-trigger system extends battery life and storage capacity by only capturing potentially useful data, instead of running continuously. During data analysis a trigger system further reduces workload by reducing the number of “empty” photos captured which still require human analysis. This system is controlled using a Raspberry Pi, which in turn runs far-red (FR) and ultraviolet (UV) LED strobes and a camera, three large and easily upgraded battery packs allows this system to run continuously for ∼72 h durations. Nickel-metal hydride (NiMH) battery chemistry was used for this project, as they were lower in cost and a safer choice when working in wet environments than lithium batteries despite being lower in energy density. An advantage to using a less detectable light frequency to detect motion is similar in purpose to the use of IR in terrestrial camera traps, as it reduces the potential of detectable artificial lighting to influence animal behavior. For example, the consistent periodic white light strobing of a time-lapse system could induce avoidance behaviors in certain animals, thereby biasing observations [8]. The FR strobes are used to illuminate the scene and are invisible to most aquatic organisms, while the UV strobes allow illumination of UV fluorescent tags in tag-recapture studies. However, other color LEDs, such as white, can easily be substituted in the strobes for other applications. The low-cost Schedule 80 PVC housing used for this system can withstand pressures up to 370 psi, allowing deployment depths in excess of 800 ft. This system has a variety of applications within benthic ecology and marine population ecology, as the unique combination of low-cost, multi-day duration, and motion detection enable efficient field work and observations of infrequent organisms to be made unobtrusively. The proposed system is also fairly simplistic, allowing for smaller scientific programs with modest technical resources to construct in-house. To our knowledge, there are currently no commercially available camera systems that are able to meet these specifications. Requirements for the camera system were (1) affordable (∼1000USD), (2) motion-detecting and autonomous, (3) long duration deployments (∼3 days)), (4) capable of detecting UV florescent markers on organisms for distinguishing individuals, (5) capable of observing organisms during day and night, and (6) be easily constructed using open-source components.

## 2 Hardware description and application

• Offers an inexpensive platform for observation of benthic organisms
• Easily customizable due to consumer electronics and open-source distribution, allowing diverse applications
• Programmable settings
• Utilizes LED arrays to illuminate the sea floor and capture nocturnal or diurnal species
• Minimizes observer bias often associated with SCUBA diving

### 2.1 Electronics

This system relies on a Raspberry Pi 3B+ (small single board computer) which controls a variety of low-cost electronics attached to a custom PCB hat (a printed circuit board attached on top of Raspberry Pi 3B+ ) (Fig. 13). The Raspberry Pi 3B+ (RPI) contains an impressive 64-bit quad core processor capable of running wireless LAN and Bluetooth. An extended 40-pin GPIO (noncommitted digital signal pin) header can be used for connection to a custom PCB, while an HDMI connection allows a monitor, keyboard and mouse to be connected, for easy access to the files, code and settings. A 64 GB microSD card holds the operating system and stores all acquired images. The PCB hat houses a Adafruit Pro Trinket microcontroller (3 V version) which operates at 12 MHz (PTM), a Adafruit PCF8523 Real Time Clock integrated circuit (RTC), a INA 219 Voltage and Current Sensor (VCS), a Pololu Electronic power switch (EPS), a 5 V power voltage regulator for powering the RPI, a Adafruit PiOLED – 128 × 32 Monochrome OLED (OLED), and a SparkFun PicoBuck LED Driver (PLD).

### 2.2 Basic system operation

The battery bank is plugged into the PCB and is routed through a fuse and connected to the EPS on the VIN and ground pins. The battery power is also connected directly to the PTM, which is powered at all times when the battery is connected to the PCB. The PTM is connected to the Blue Robotics underwater switch. When the switch is activated, the PTM sends a logic signal to the EPS to provide power to the RPI and PLD. The RPI then boots up and activates the OLED, starting the image acquisition process. The PTM also serves as the strobe channel selector as the strobe output pin from the RPI is separate from the strobe channel pin. The OLED, RTC, and VCS) are all connected to the RPI i2c interface for communication.

## 3 Design files

**Table 1:** Design Files Summary, DF1-DF9 are 3D mesh files intended for direct 3D printing of each component. Software source code DF10-DF11 compose the operating system and can be downloaded or flashed onto a 64 GB microSD card for use with the Raspberry Pi 3B+. Kicad file DF12, acts as a template for printing of custom PCB’s and is based on a free software suite KiCad for PCB manufacturing and layout.

Design file ID	Design file name	File type	Open source license	Location of the file
DF1	port_mount_spreader.stl	3D mesh	CC BY-SA 4.0	Zenodo
DF2	port_mount.stl	3D mesh	CC BY-SA 4.0	Zenodo
DF3	camera_mount.stl	3D mesh	CC BY-SA 4.0	Zenodo
DF4	led_holder_base.stl	3D mesh	CC BY-SA 4.0	Zenodo
DF5	led_holder.stl	3D mesh	CC BY-SA 4.0	Zenodo
DF6	battery_holder_cap.stl	3D mesh	CC BY-SA 4.0	Zenodo
DF7	battery_holder_middle.stl	3D mesh	CC BY-SA 4.0	Zenodo
DF8	pi_bulkhead.stl	3D mesh	CC BY-SA 4.0	Zenodo
DF9	front_bulkhead.stl	3D mesh	CC BY-SA 4.0	Zenodo
DF10	trigger_camera_disk.img	Software source code	CC BY-SA 4.0	Zenodo
DF11	Pro_trinket_code.ino	Software source code	CC BY-SA 4.0	Zenodo
DF12	Trigcam_hat_Final.kicad	Kicad file	CC BY-SA 4.0	Zenodo

## 4 Bill of Materials

**Table 2:** Bill of Materials.

Part #	Component	Mfr. Model #	Number Used	Cost per Unit (USD$)	Total Cost (USD$)	Component Source	Material Type
**P1**	Custom PCB hat	NA	1	∼20.00	∼20.00	JLCPCB	Other
**P2**	10 K ohm resistors (0.5 Watt)	611355173112	4	5.99 (100)	5.99 (100)	Amazon	Other
**P3**	5 Amp Fuse	0297005.WXNV	1	0.29	0.29	Digi-Key	Other
**P4**	Fuse clip	3544-2	1	0.95	0.95	Mouser	Other
**P5**	I2C bus (4 pin male header)	B09MYF8XPC	1	7.99	7.99	Amazon	Other
**P6**	Preci dip 2 pin angle header (female)	801-83-002-20-001101	1	0.52	0.52	Mouser	Other
**P7**	6 pin angle header (male)	90122-0123	1	1.94	1.94	Mouser	Other
**P8**	Dual Row Tin 6 pin Header	76825-0006	1	2.73	2.73	Mouser	Other
**P9**	Pololu Electronic power switch	2812	1	5.95	5.95	Pololu	Other
**P10**	Picobuck LED driver	COM-13705	1	17.50	17.50	Sparkfun	Other
**P11**	Screw Terminals 3.5 mm Pitch (2 pin)	1729,128	3	1.05	3.15	Sparkfun	Other
**P12**	Adafruit Pro Trinket 3 V 12 MHz	2010	1	9.95	9.95	Adafruit	Other
**P13**	INA 219 I2C Current Sensor	B01ICN5OAM	1	6.99	6.99	Amazon	Other
**P14**	5 V 3A Output Voltage Regulator	B0823QLMWC	1	12.99	12.99	Amazon	Other
**P15**	Adafruit PCF8523 Real Time Clock	3295	1	6.95	6.95	Adafruit	Other
**P16**	LiCB 3 V Clock Battery	B0797NRXZY	1	5.00	5.00	Amazon	Other
**P17**	Adafruit PiOLED 128x32 Monochrome OLED	3527	1	7.99	7.99	Adafruit	Other
**P18**	40 pin female header connector	PRT-16764	1	1.95	1.95	Digi-Key	Other
**P19**	Raspberry Pi 3B+	5,060,214,370,165	1	35.00	35.00	PiShop.us	Other
**P20**	10 cm Female to Female jumper	B07S2RH6Q4	1	5.49	5.49	Amazon	Other
**P21**	Arducam Wide-Angle CS-Mount lens	B088BLZKRG	1	15.99	15.99	Amazon	Other
**P22**	Flex Cable for Raspberry Pi Camera	A1 FFCs	1	13.99	13.99	Amazon	Other
**P23**	Raspberry Pi HQ Camera	0633696492738	1	50.00	50.00	PiShop.us	Other
**P24**	Molex 6 Circuit Wire Connector	39121400	1	10.99	10.99	Amazon	Other
**P25**	Wiring 20 AWG (100ft of red and black)	B07K9JKXM9	1	23.98	23.98	Amazon	Other
**P26**	Machine screw M5-0.8 nut	B07CDZMXYR	2	8.27	8.27	Amazon	Metal
**P27**	Machine screw pan head Philips M5-0.8 × 50 mm	B00918KNBI	2	8.27	8.27	Amazon	Metal
**P28**	Sheet metal screws #6 × ¾”	B08SJ11HG7	1	8.49	8.49	Amazon	Metal
**P29**	#2-56 UNC Machine Screws	NA	1	11.99	11.99	Amazon	Metal
**P30**	Sheet metal screws #4 × ½”	B08P2J19WM	1	6.98	6.98	Amazon	Metal
**P31**	530 nm Starboard UV LED	XPEBGR-L1-0000-00F03-SB01	2	6.65	13.3	Digi-Key	Other
**P32**	FR LED’s (INDUS STAR A008)	A008-CE20FAR27	1	9.99	9.99	Digi-Key	Other
**P33**	12 V 4500mAh Ni-Mh Battery (Pack of 2)	945-0129	3	49.99	149.97	Amazon	Other
**P34**	Molex Female freehang Mega-Fit plug	1716920106	3	1.11	3.33	Digi-Key	Other
**P35**	Molex Female plug tin crimp pin	0768230321	100	0.1611	16.11	Digi-Key	Metal
**P36**	#6–32 × 36in threaded rod	52102	4	4.99	19.96	ACE	Metal
**P37**	#6–32 Machine Hex Nut	B07ZWDDGPS	1	9.40	9.40	Amazon	Metal
**P38**	Molex Male freehang Mega-Fit plug	1054110106	4	1.19	4.76	Digi-Key	Other
**P39**	Molex Male plug tin crimp pins	1054170334	100	0.187	18.71	Digi-Key	Other
**P40**	Schedule 80 PVC 3 in. Pipe 10ft	H0800300PG1000	1	91.82	91.82	Grainger	Polymer
**P41**	Schedule 80 PVC Pipe cap	847-030	1	24.39	24.39	Grainger	Polymer
**P42**	Schedule 80 PVC *T*-joint	801-030	2	28.90	57.8	Grainger	Polymer
**P43**	Schedule 80 PVC Union joint	897-030	3	40.96	122.88	Grainger	Polymer
**P44**	½ in. Plexiglass	SL-AS13-12x12	1	22.95	22.95	Amazon	Polymer
**P45**	¼ in. annealed glass plate		1	NA	NA	Amazon	Glass
**P46**	BlueRobotics High Pressure Switch	SWITCH-M10-5A-R1-RP	1	20.00	20.00	Blue Robotics	Other
**P47**	Charger for 9.6 V-18 V NiMh/NiCd Battery Packs	H02400918-US-1	2	24.95	49.9	BatterySpace.com	Other
**P48**	Schedule 80 PVC cement grey	20603	1	8.01	8.01	Grainger	Other
**P49**	Silicone-Based vacuum grease	013161037532	1	32.95	32.95	Amazon	Other
**P50**	Rechargeable desiccant packets	634301317538	1	10.98	10.98	Amazon	Other
**P51**	SanDisk 64 GB MicroSD card	B08GYBBBBH	1	11.99	11.99	Amazon	Other
**P52**	0.6 mm Solder Wire	B07PBD71V2	1	15.99	15.99	Amazon	Metal
**P53**	Heat shrink tubing	B089D82FLG	1	13.99	13.99	Amazon	Polymer

## 5 Build instructions

Step-by-step instructions to assemble the PCB hat, batteries, wiring and 3d-printed structural components. Refer to parts list (Table 2), design files (Table 1), and visual aids (Fig. 1, Fig. 2, Fig. 3, Fig. 4, Fig. 5, Fig. 6, Fig. 7, Fig. 8, Fig. 9, Fig. 10, Fig. 11, Fig. 12, Fig. 13, Fig. 14, Fig. 15, Fig. 16, Fig. 17, Fig. 18, Fig. 19, Fig. 20, Fig. 21, Fig. 22, Fig. 23, Fig. 24, Fig. 25, Fig. 26, Fig. 27, Fig. 28, Fig. 29, Fig. 30, Fig. 31, Fig. 32, Fig. 33, Fig. 34, Fig. 35, Fig. 36, Fig. 37, Fig. 38, Fig. 39, Fig. 40, Fig. 41, Fig. 42, Fig. 43, Fig. 44, Fig. 45, Fig. 46, Fig. 47, Fig. 48, Fig. 49, Fig. 50, Fig. 51, Fig. 52, Fig. 53, Fig. 54, Fig. 55) for necessary components and assembly process.

**Fig. 1:**
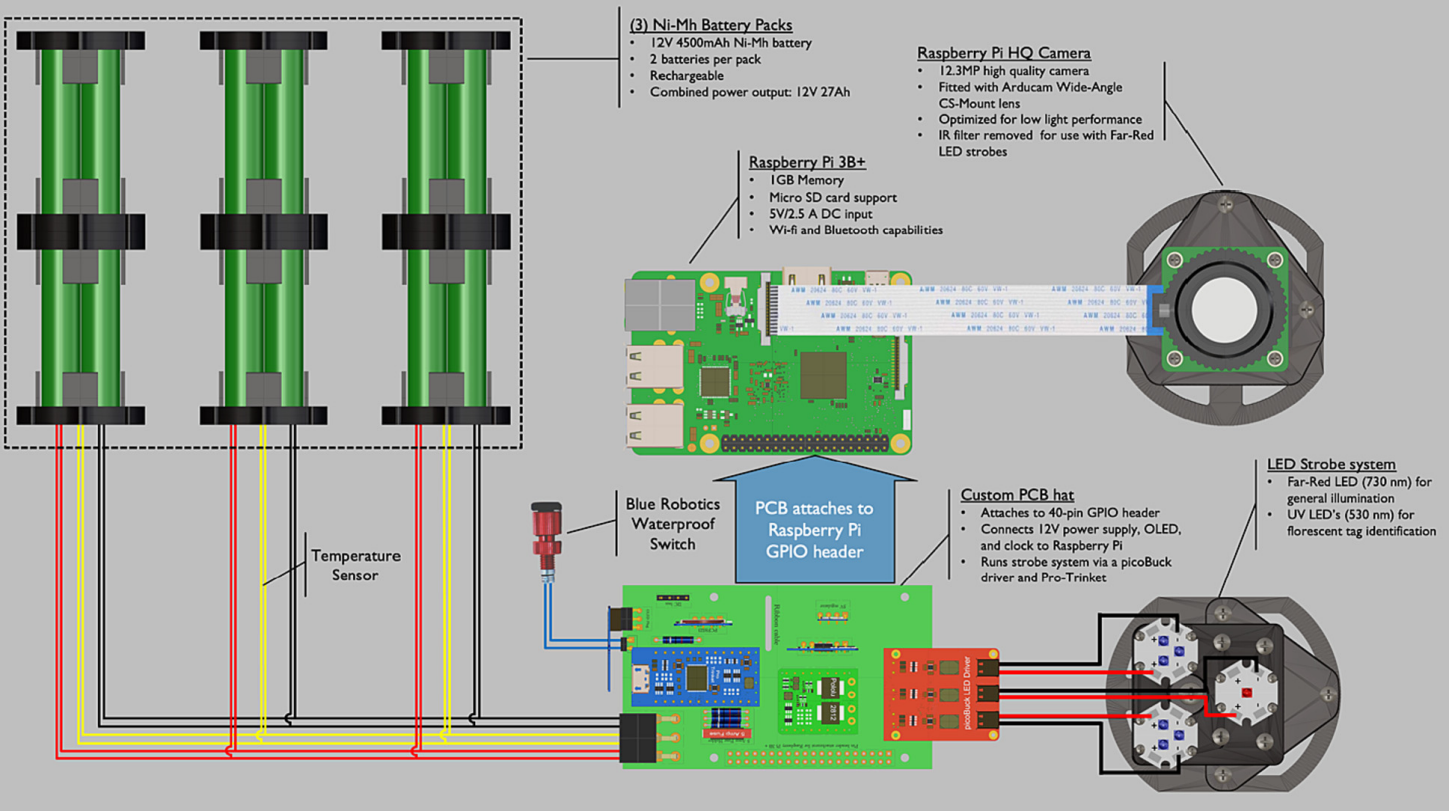
Overview of wiring diagram and key functional components.

**Fig. 2:**
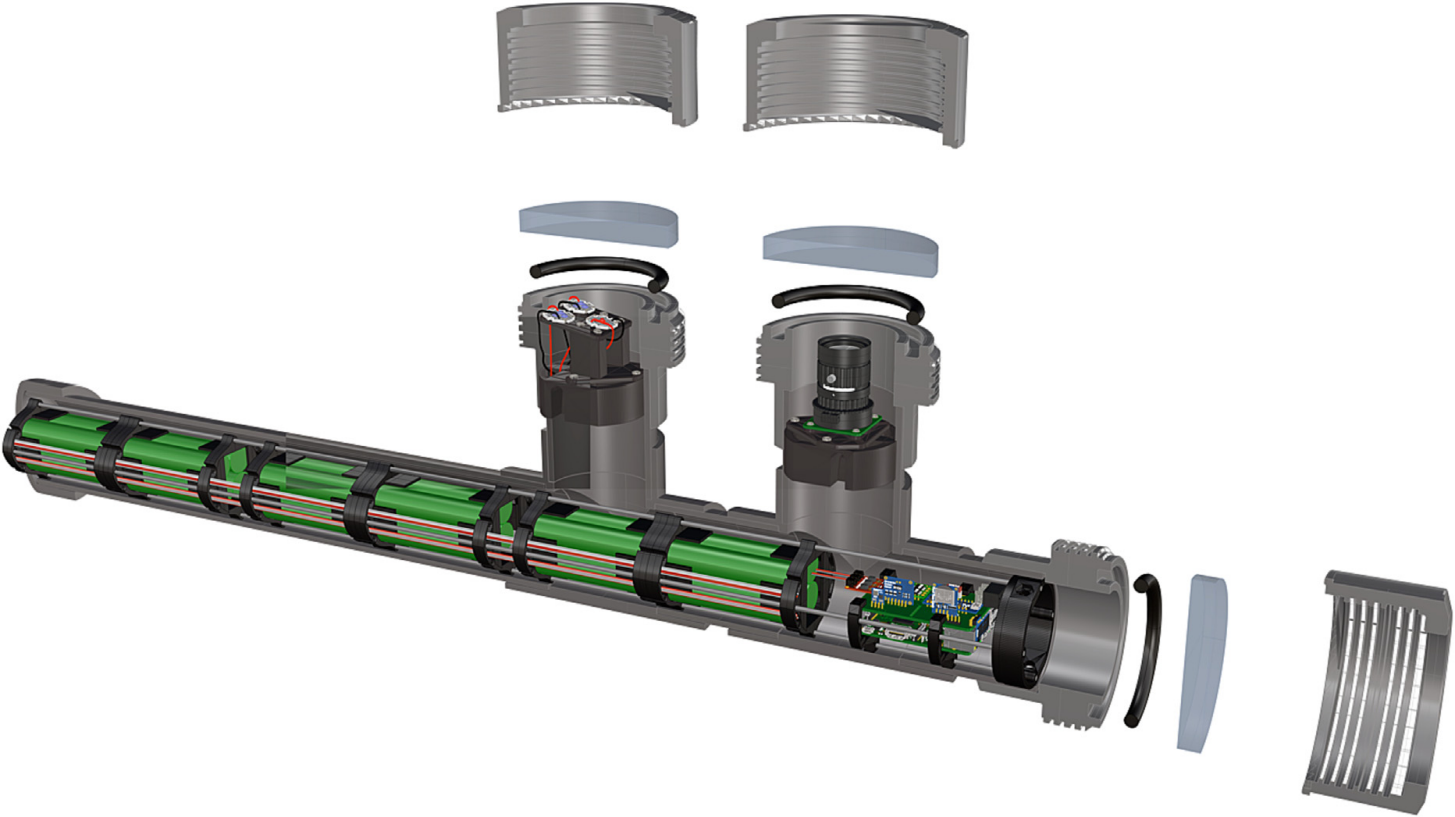
Overview of key functional components within housing.

**Fig. 3:**
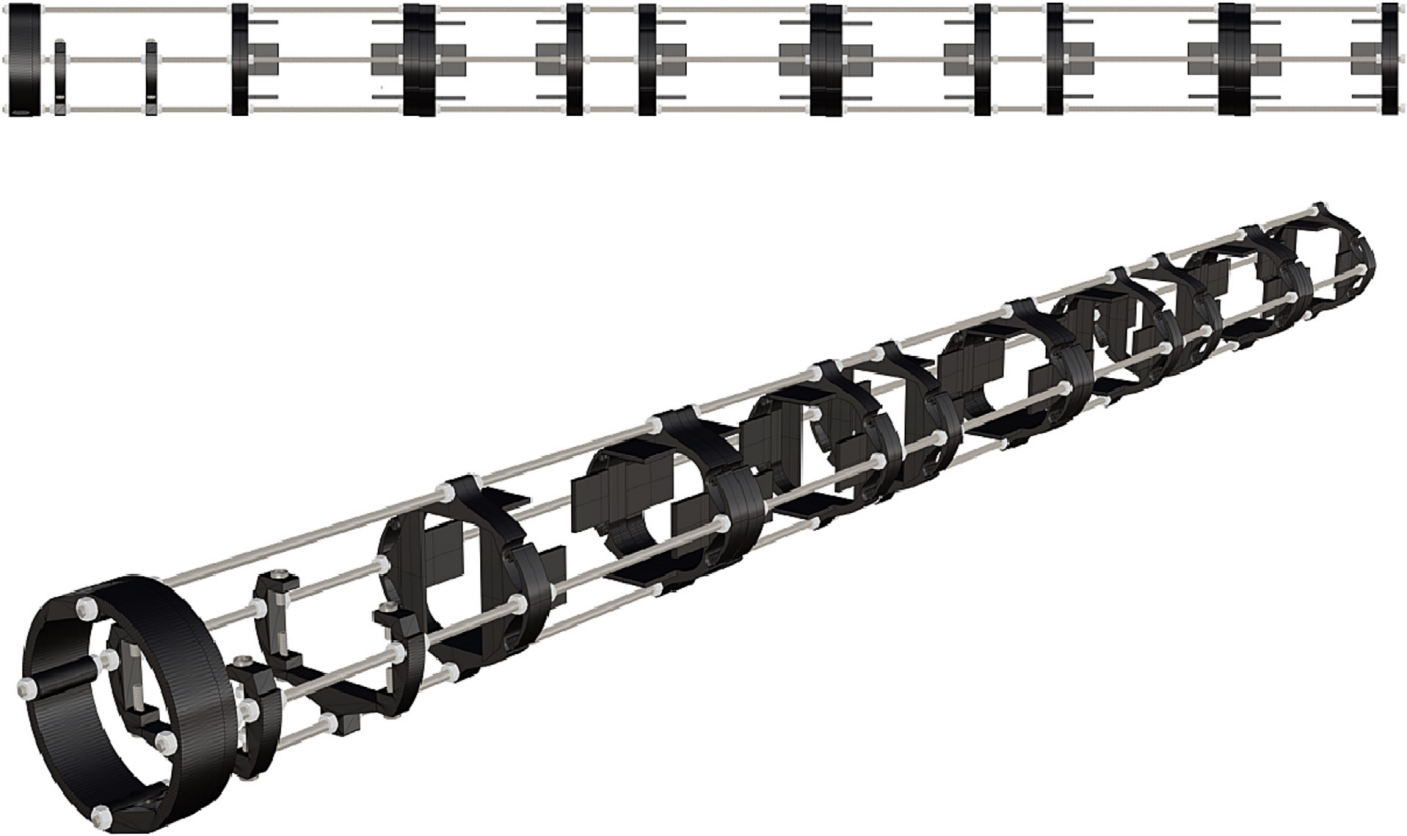
Overview of key structural components; battery and PCB mounts.

**Fig. 4:**
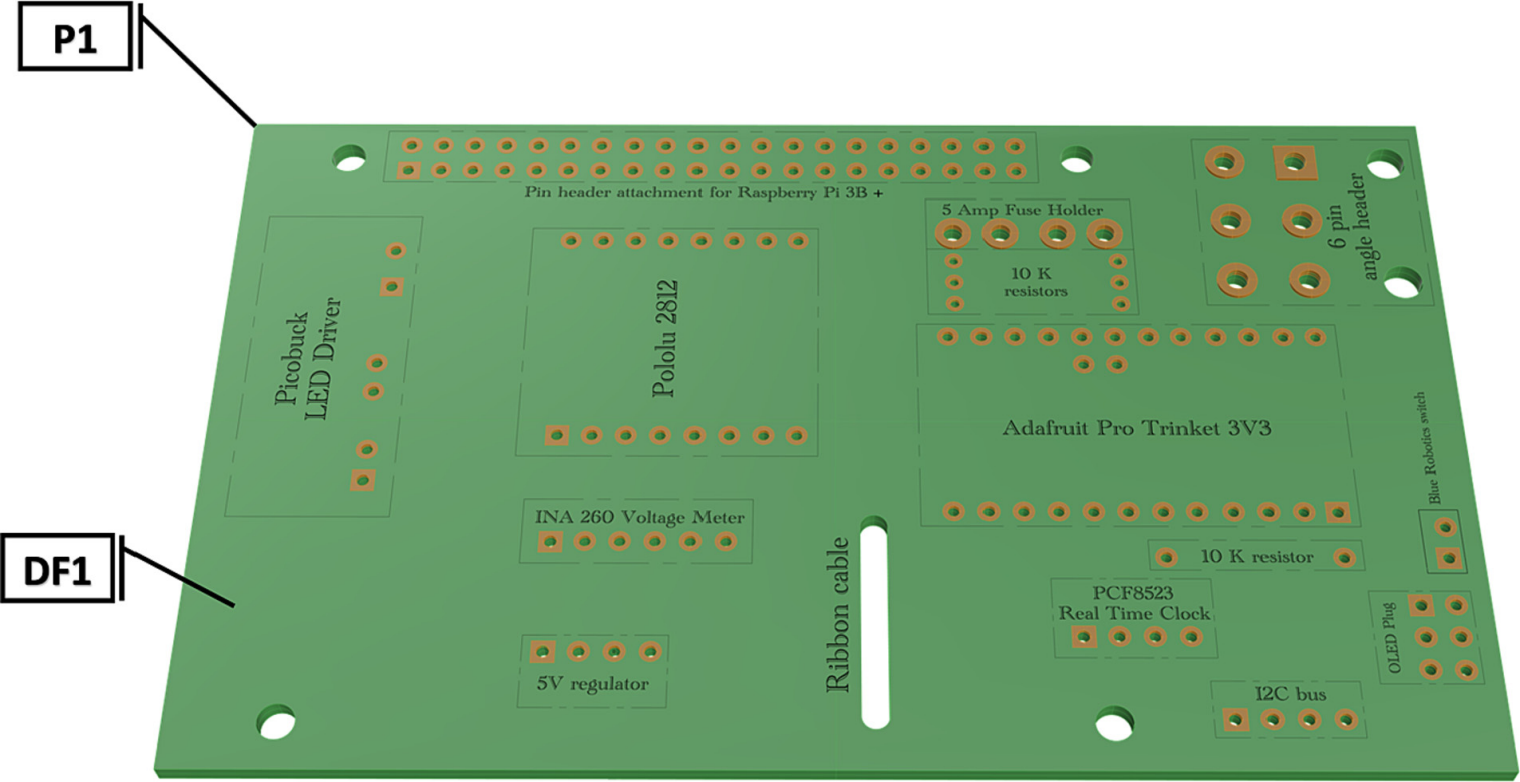
Bare PCB with labeled component placement locations.

**Fig. 5:**
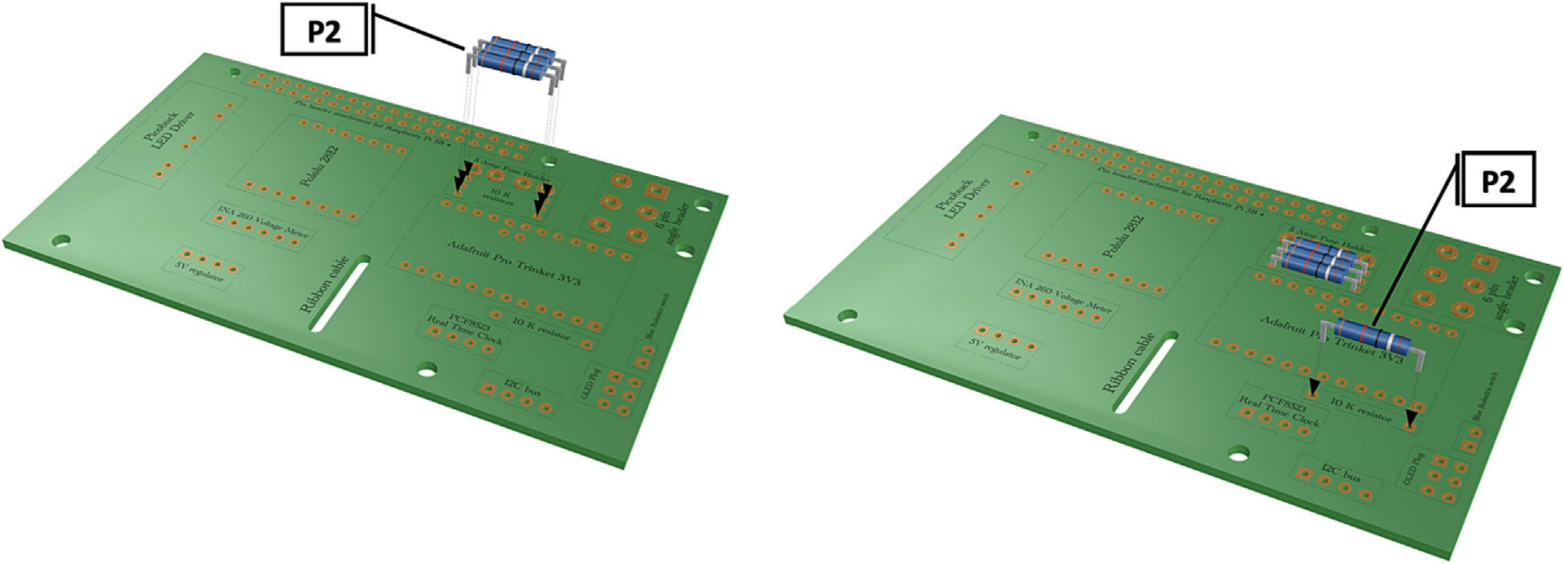
Installation of 10 K resistors.

**Fig. 6:**
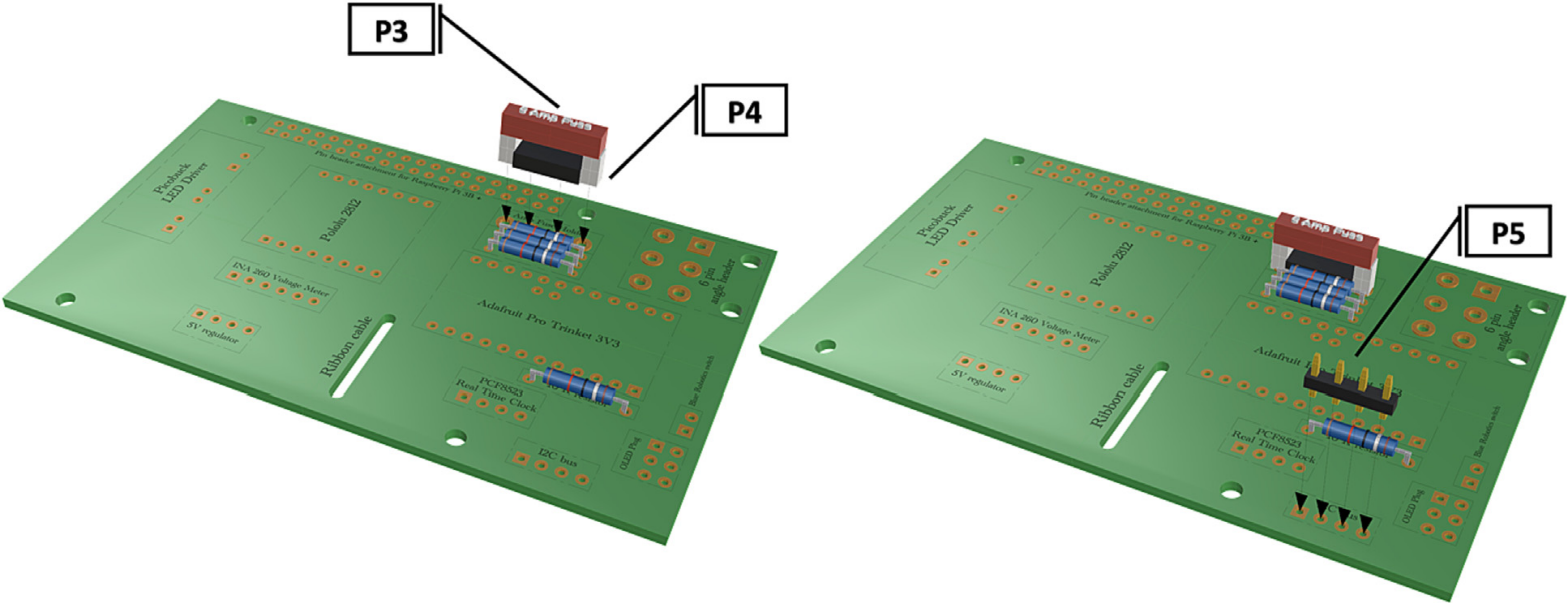
Installation of 5Amp fuse and fuse clip, installation of I2C bus for later connection to Raspberry Pi pins via a female-to-female 10 cm jumper wire (**P20**).

**Fig. 7:**
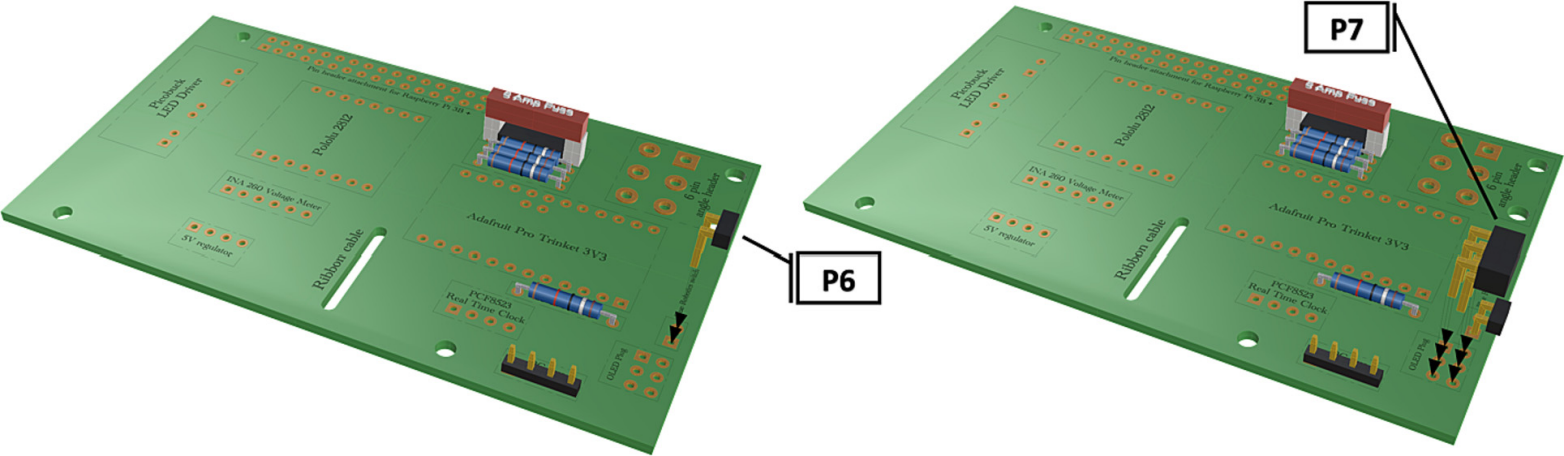
Installation of two pin header for connection to Blue Robotics switch (**P46**), installation of 6 pin angled header for later attachment of PiOLED (**P17**). (For interpretation of the references to color in this figure legend, the reader is referred to the web version of this article.)

**Fig. 8:**
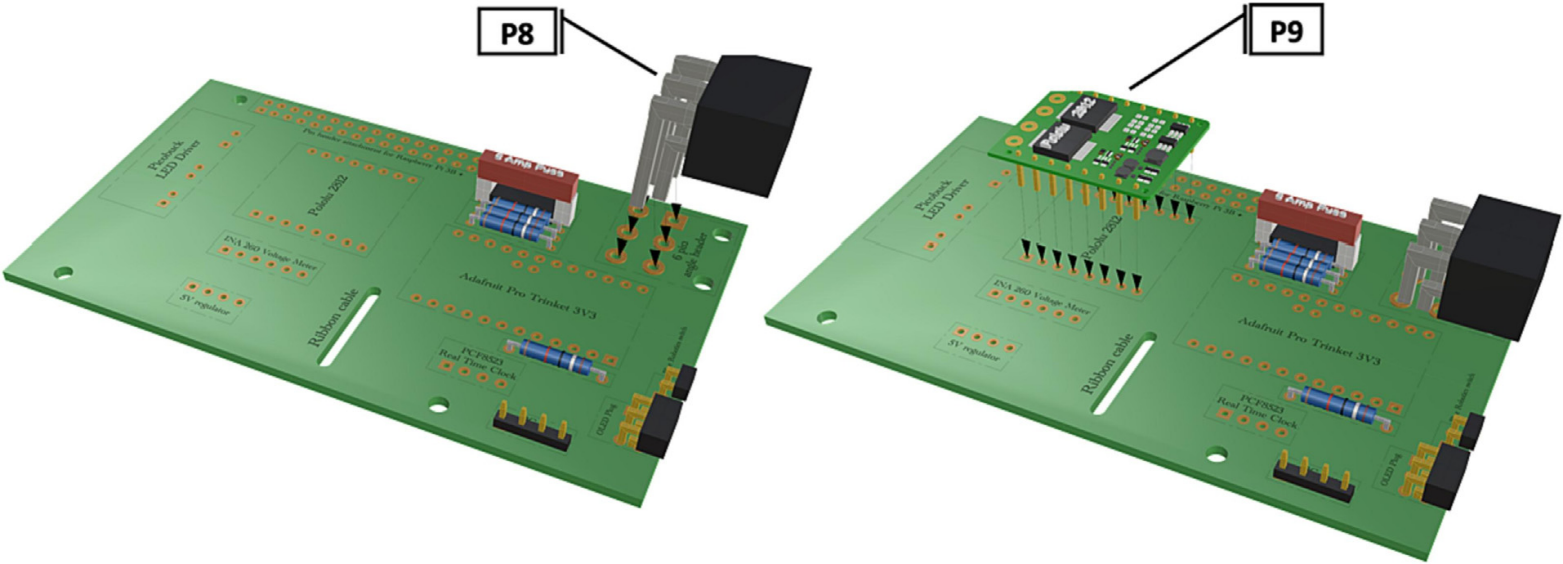
Installation of 6 pin header, power input from battery pack wiring harness, installation of Pololu power switch.

**Fig. 9:**
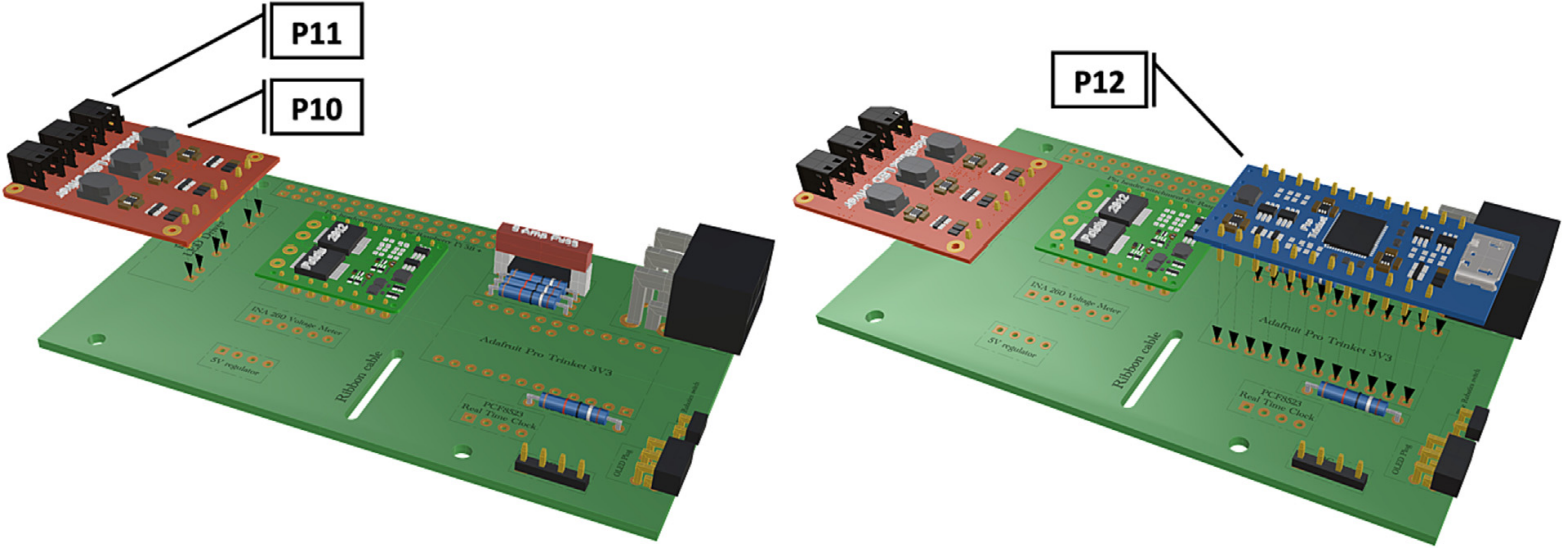
Installation of Picobuck LED driver with attached screw terminals, installation of Pro Trinket.

**Fig. 10:**
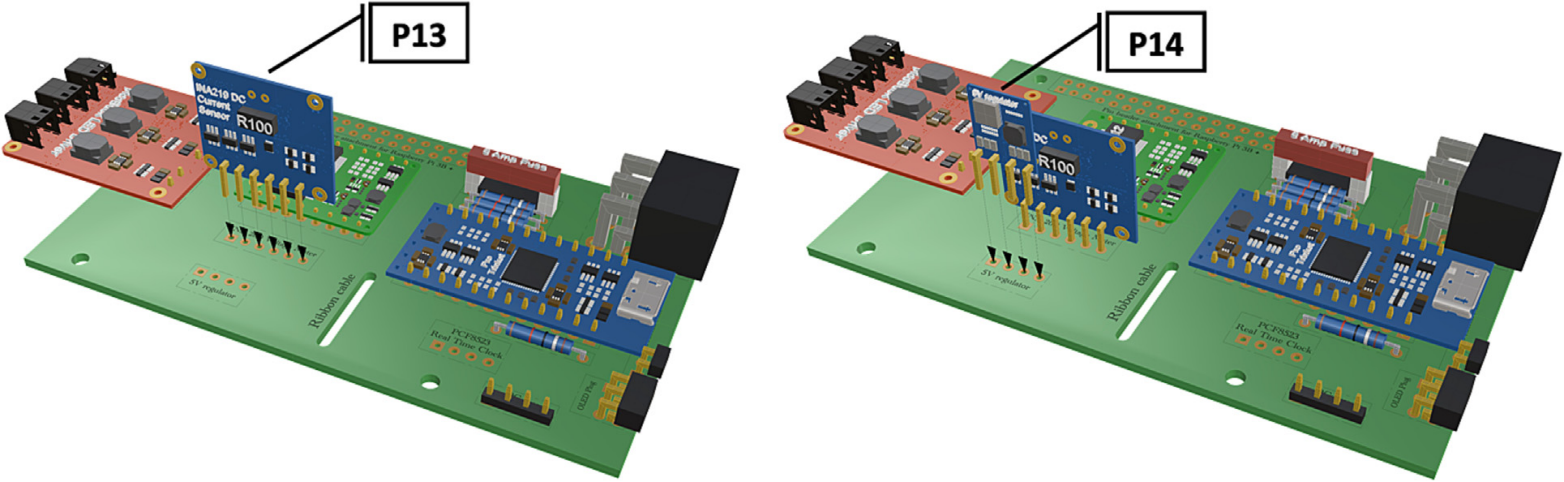
Installation of current sensor and 5 V regulator.

**Fig. 11:**
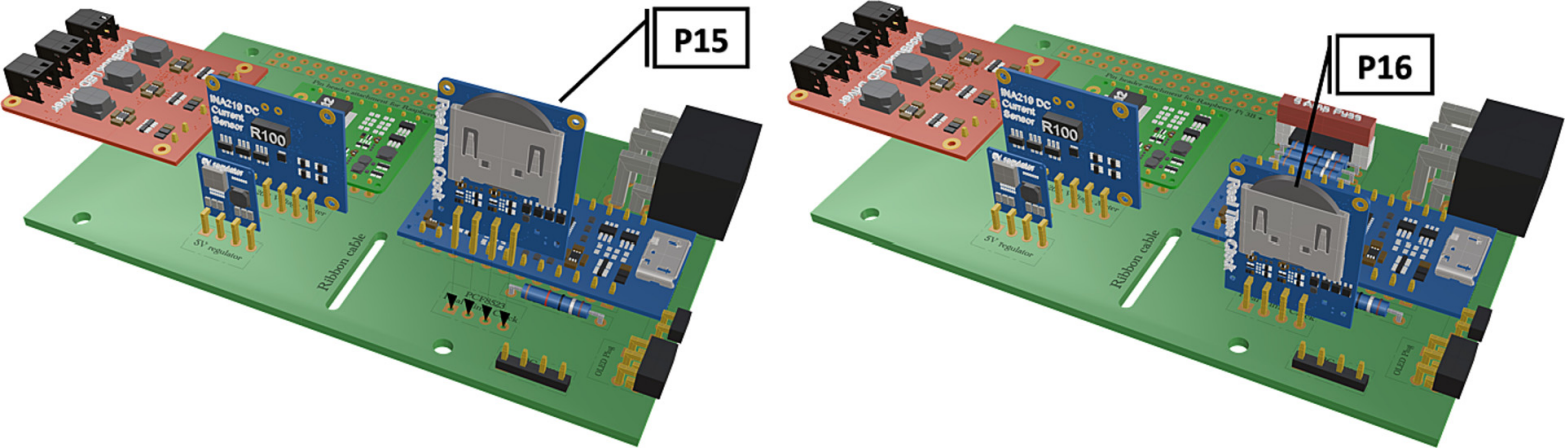
Installation of real time clock with inserted LiCB 3 V clock battery.

**Fig. 12:**
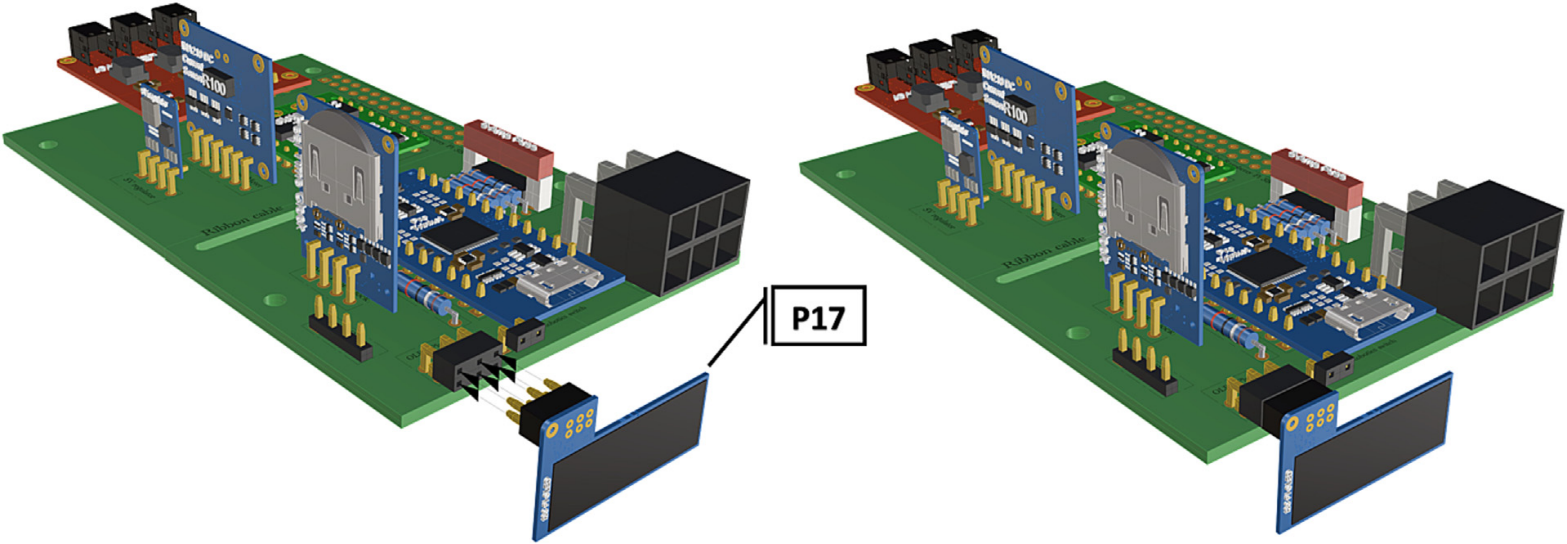
Insertion of PiOLED into 6 pin header (**P7**).

**Fig. 13:**
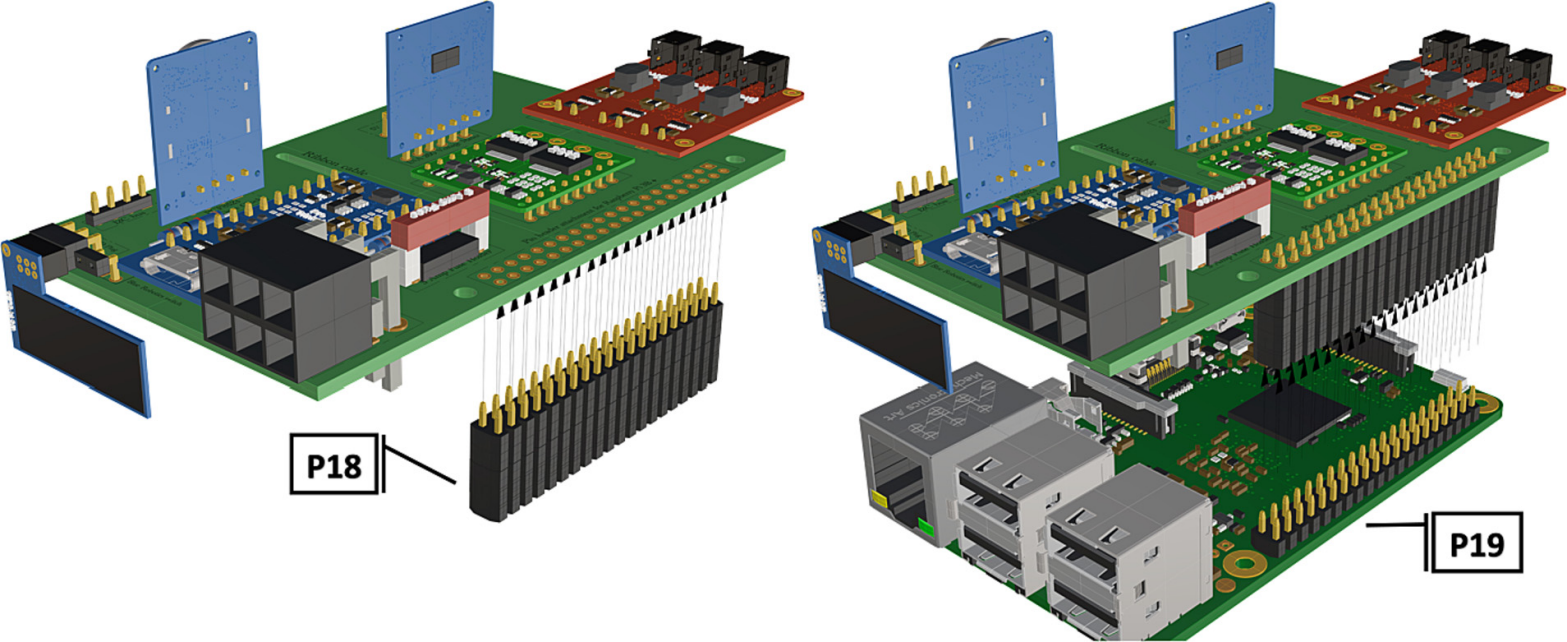
40 pin female header connector soldered to PCB header, Raspberry Pi 3B+  inserted into 40 pin female header.

**Fig. 14:**
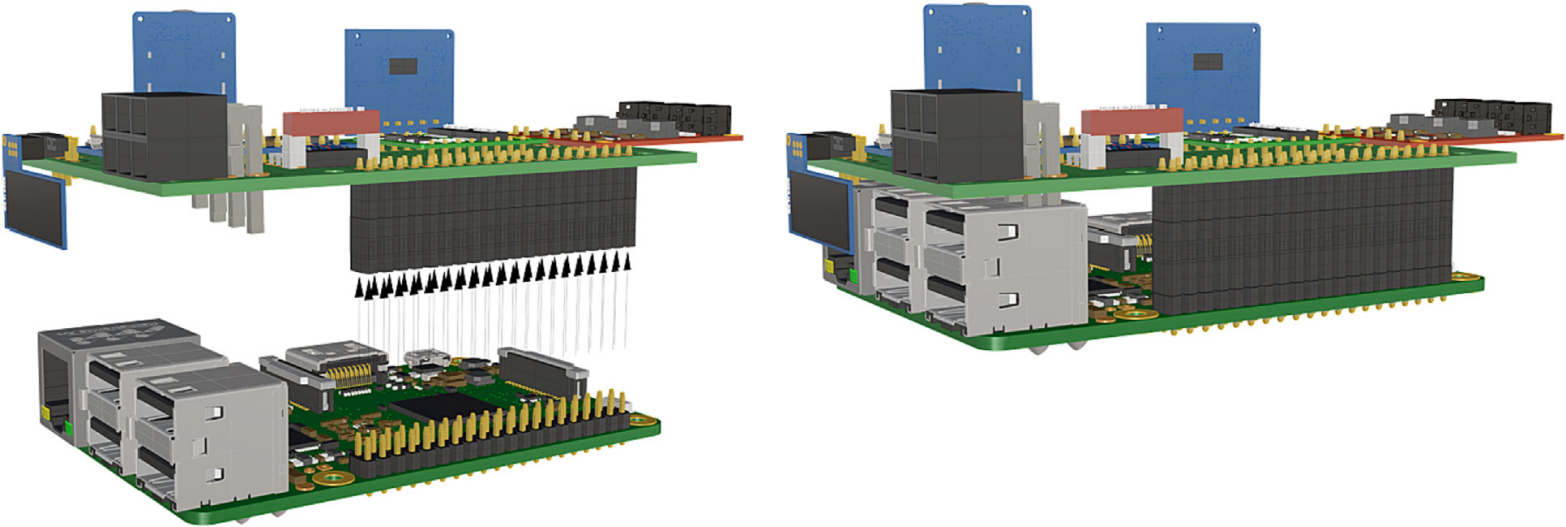
Alternate view of completed PCB ready for attachment, with side view of connected components.

**Fig. 15:**
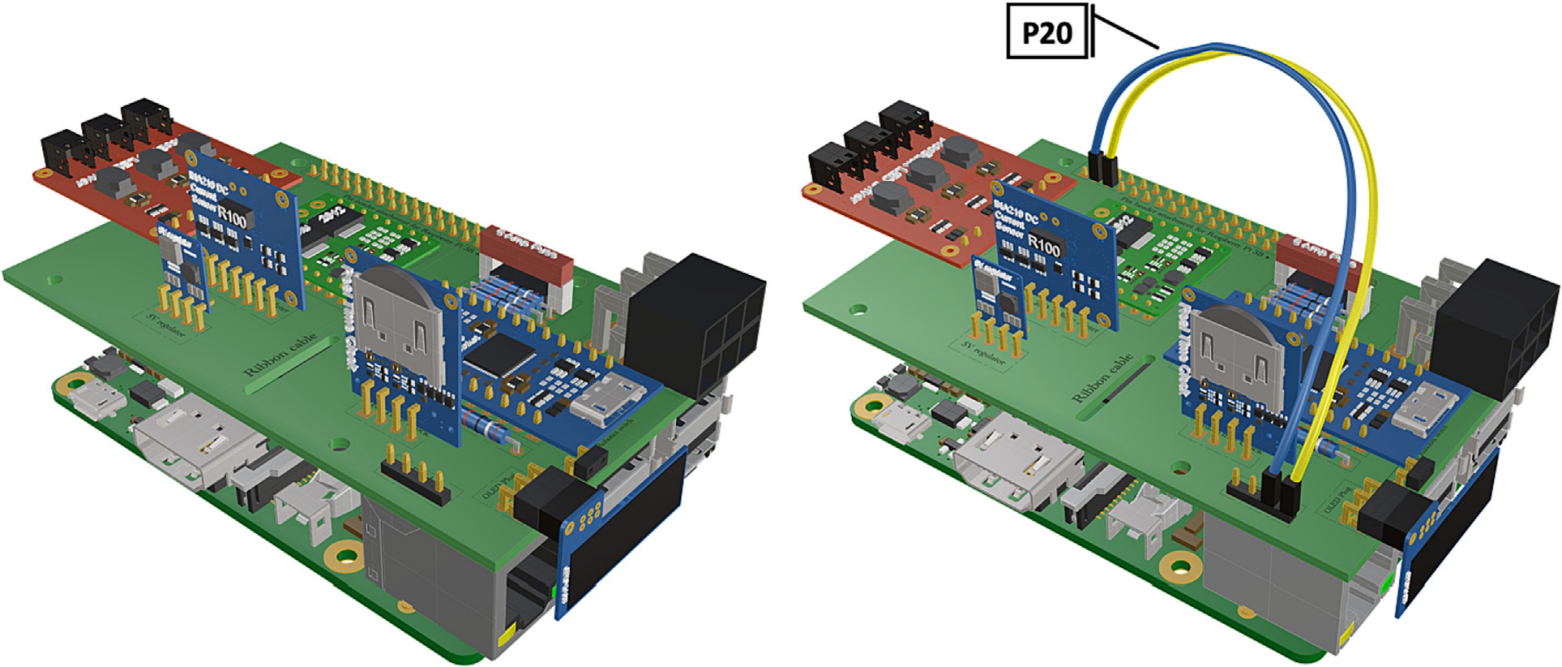
Close up of PCB with attached Raspberry Pi 3B+*,* female to female jumpers connecting PiOLED with Rasbperry Pi pins GPIO2 & 3.

**Fig. 16:**
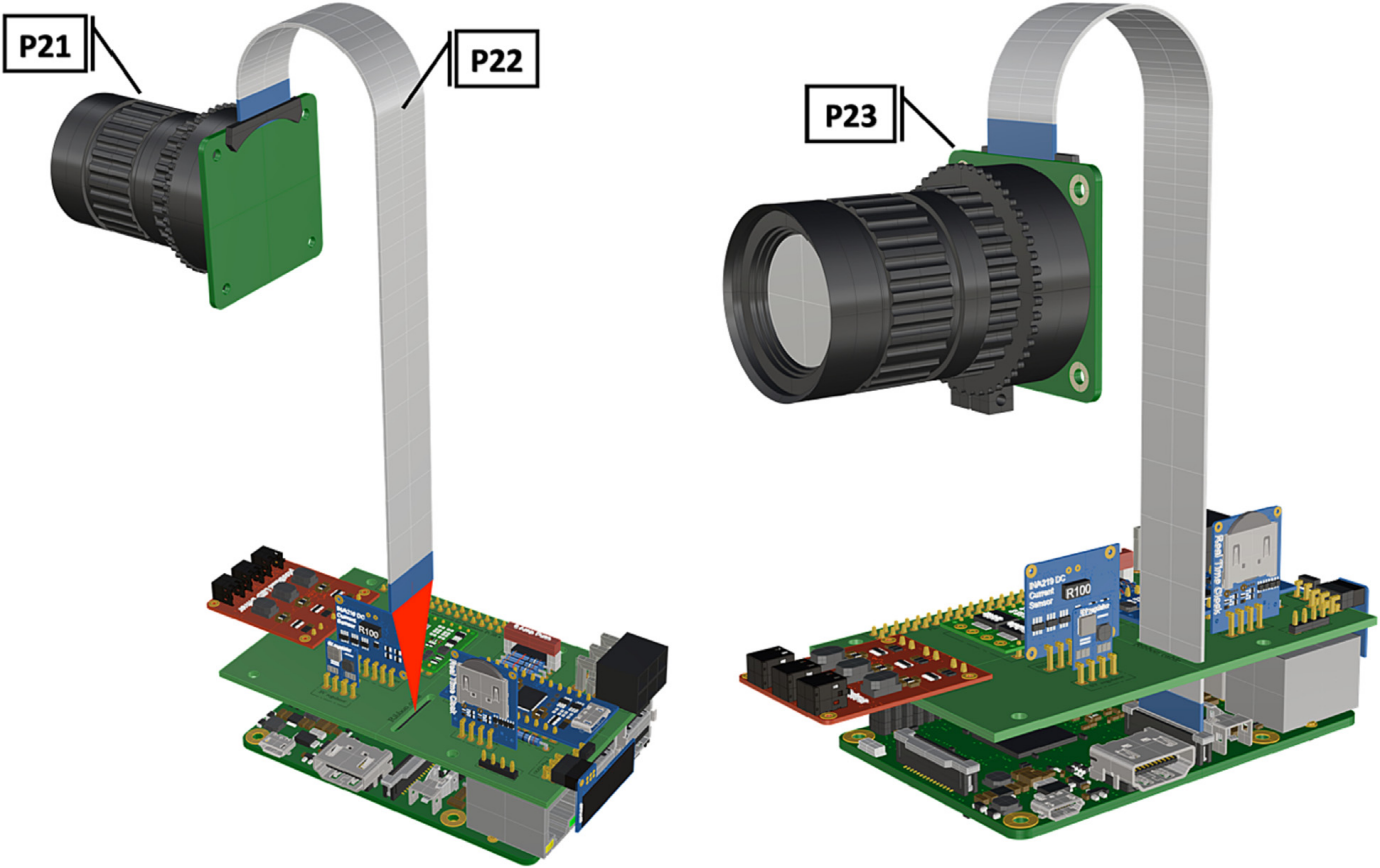
Attachment of Arducam ribbon cable to Raspberry Pi CSI port.

**Fig. 17:**
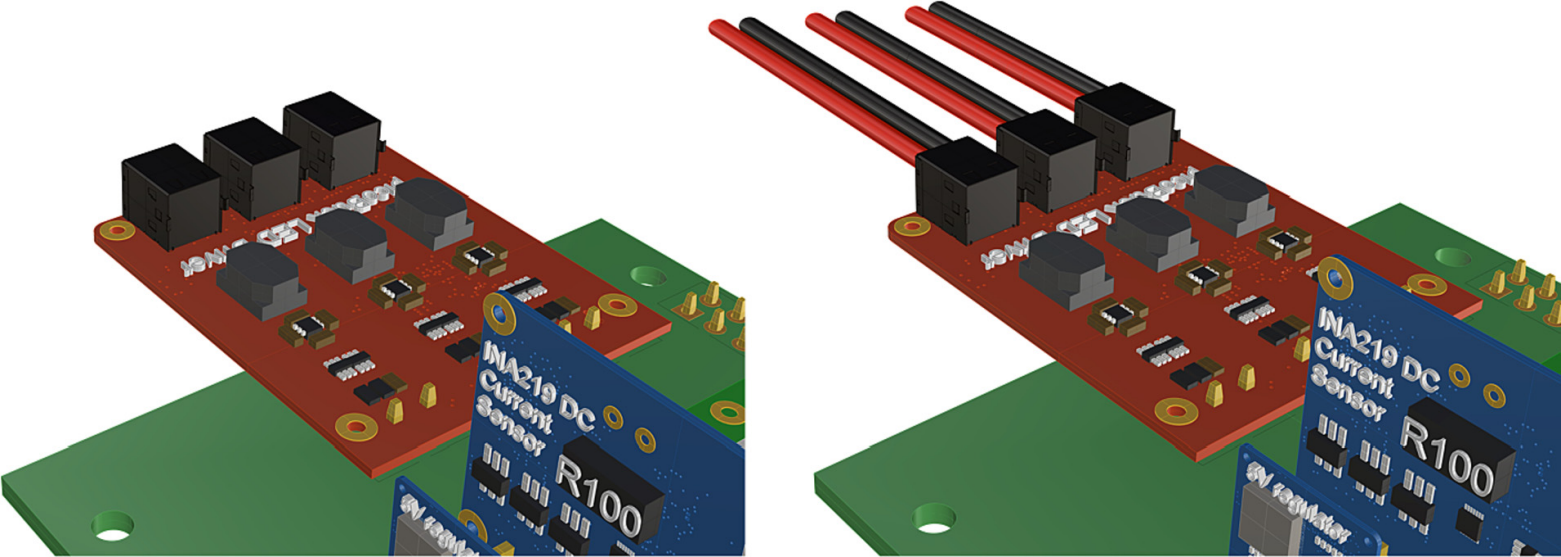
Wiring connected to picoBuck LED driver allowing LED control during deployment.

**Fig. 18:**
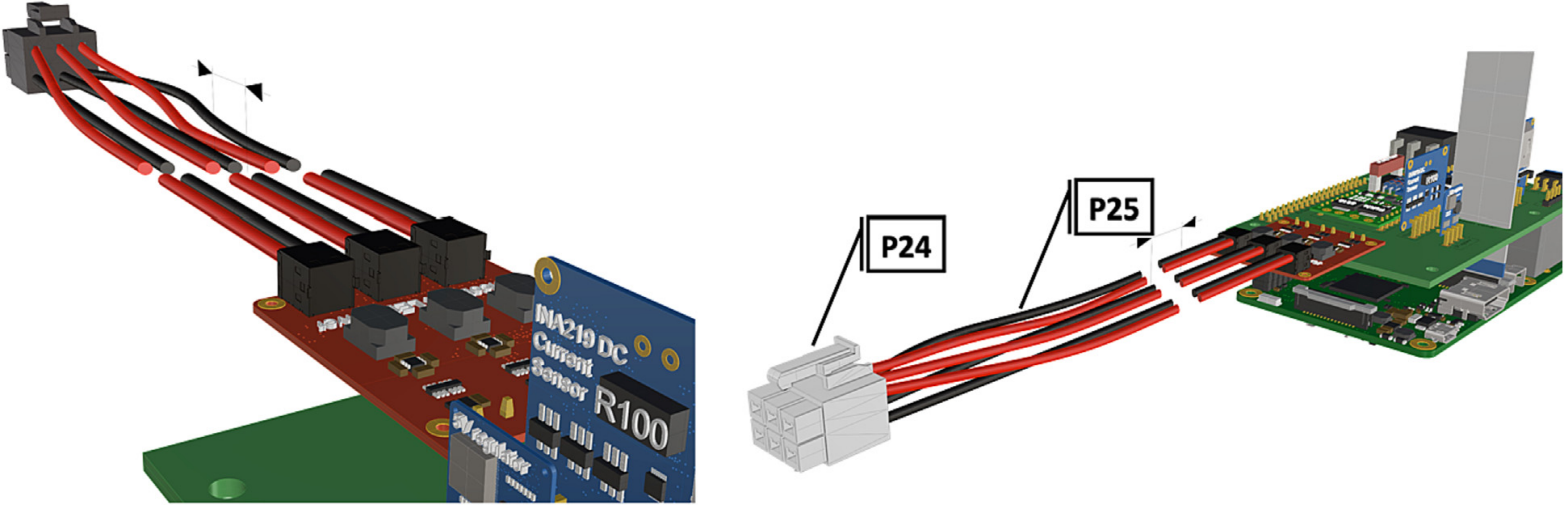
Wiring connected to male Molex 6 pin connector, allowing the disconnection of strobe system during maintenance and charging. Break in wiring represents 10–12 in. depending on desired length of LED connection.

**Fig. 19:**
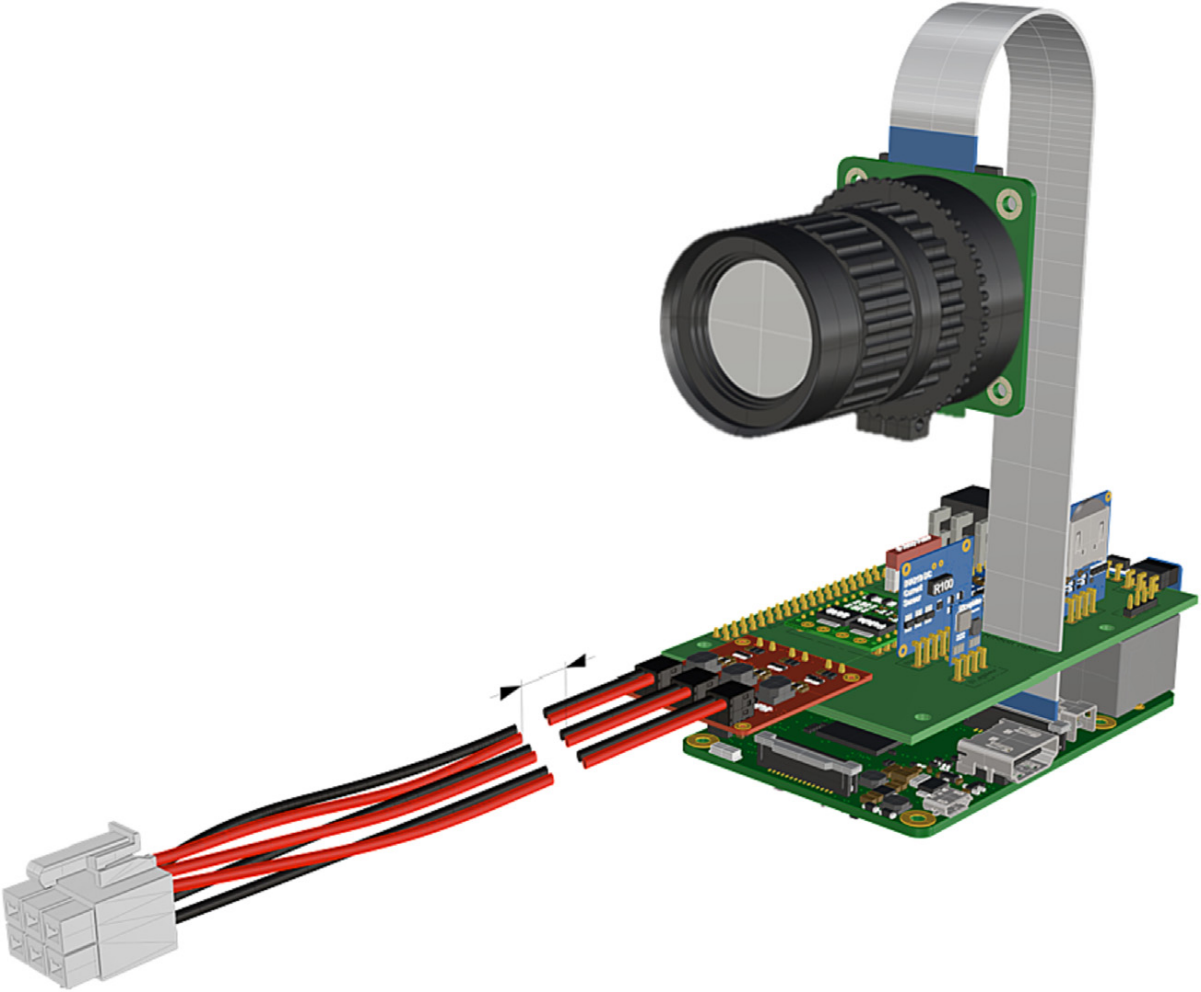
Assembled computer components with attachments of strobe and camera.

**Fig. 20:**
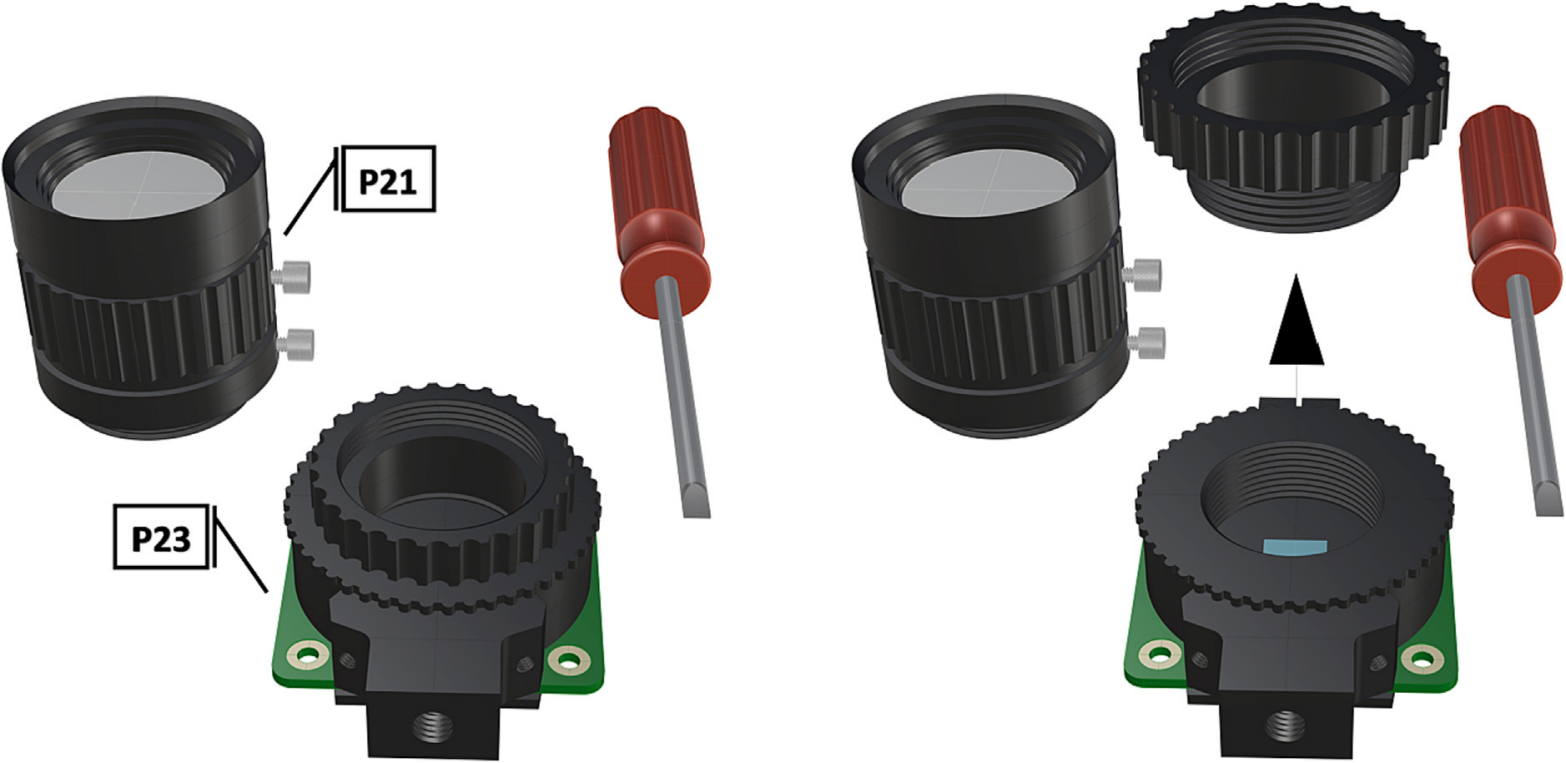
Unaltered camera components, followed by removal of CS-mount adapter attachment ring.

**Fig. 21:**
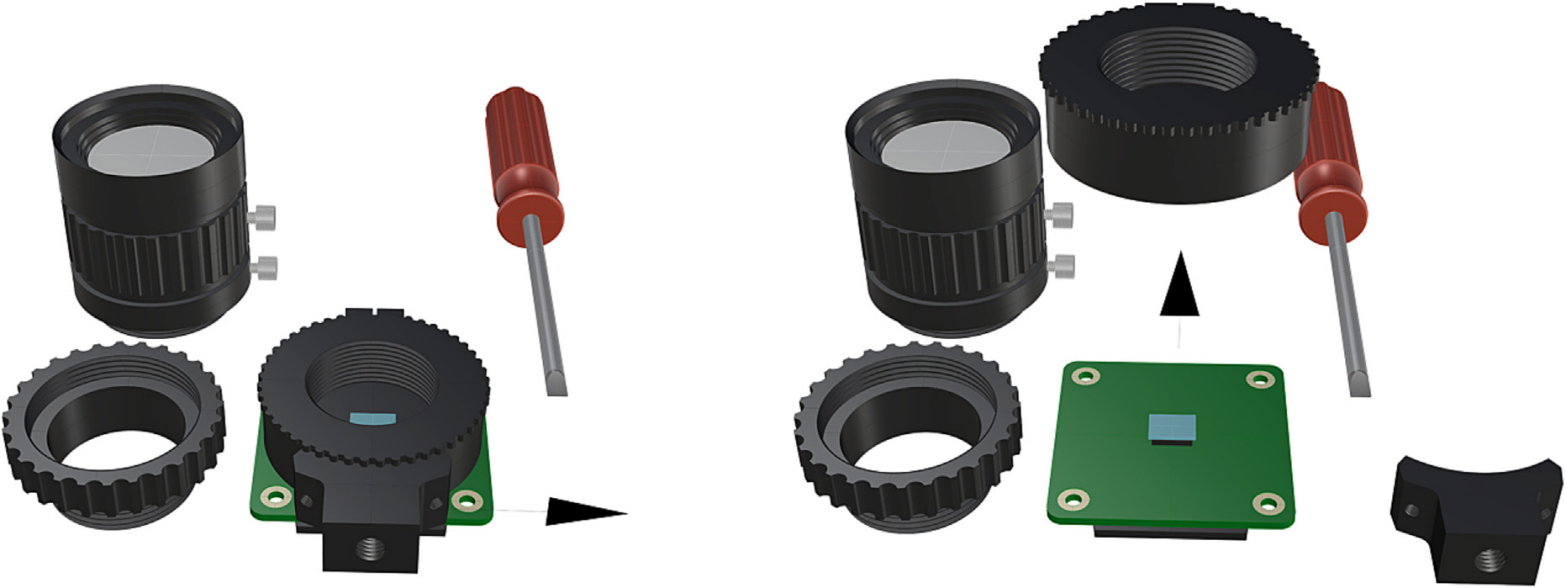
Permanent removal of integrated 1/4″-20 tripod mount followed by removal of the two 1.5 mm hex lock keys on the underside of the main circuit board in order to remove the lens mount and expose the FR filter.

**Fig. 22:**
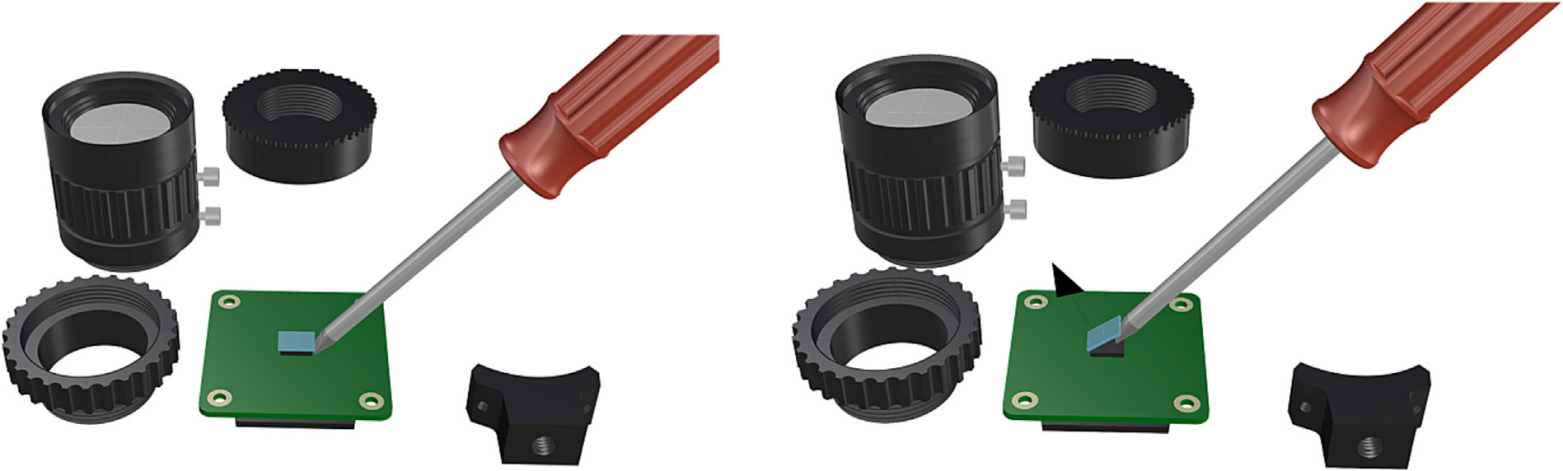
A flathead screwdriver can be used to remove the FR filter from the Sony IMX477 sensor, removal of the IR filter exposing the sensor.

**Fig. 23:**
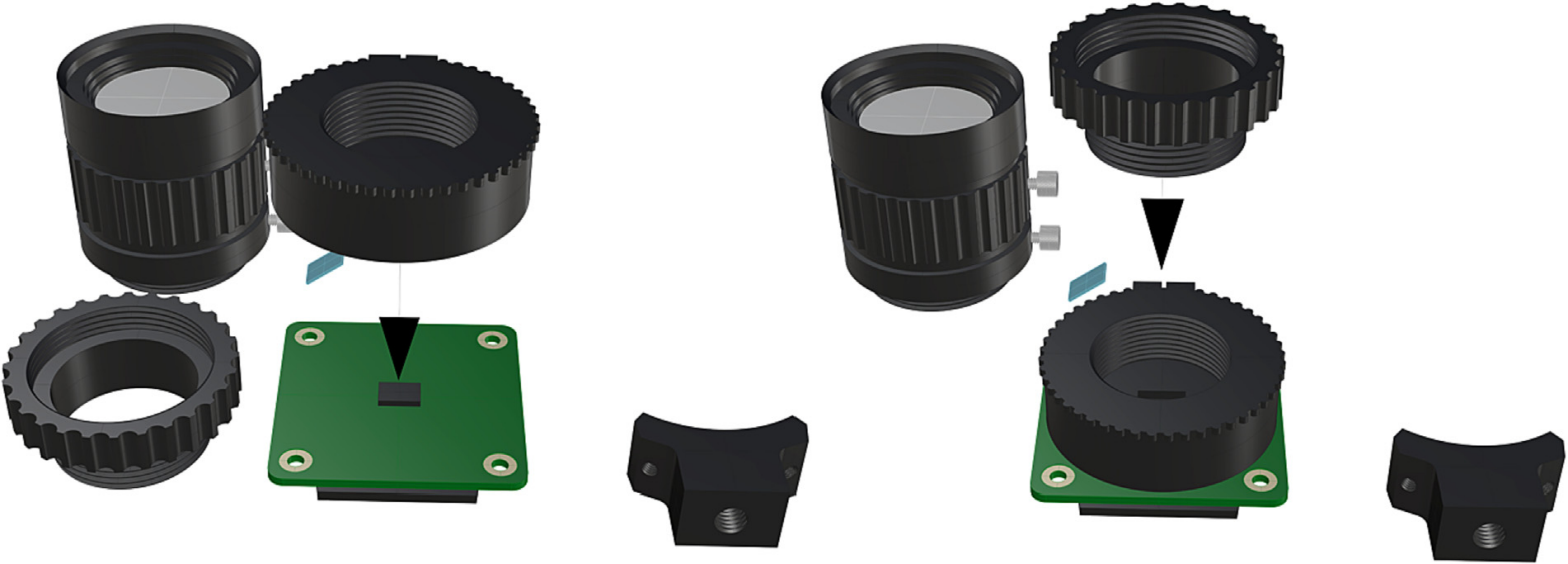
Reinstallation of lens mount by reattaching hew lock keys to underside of the main circuit board, replacement of CS-mount adapter ring for use with wide angle lens (**P21**).

**Fig. 24:**
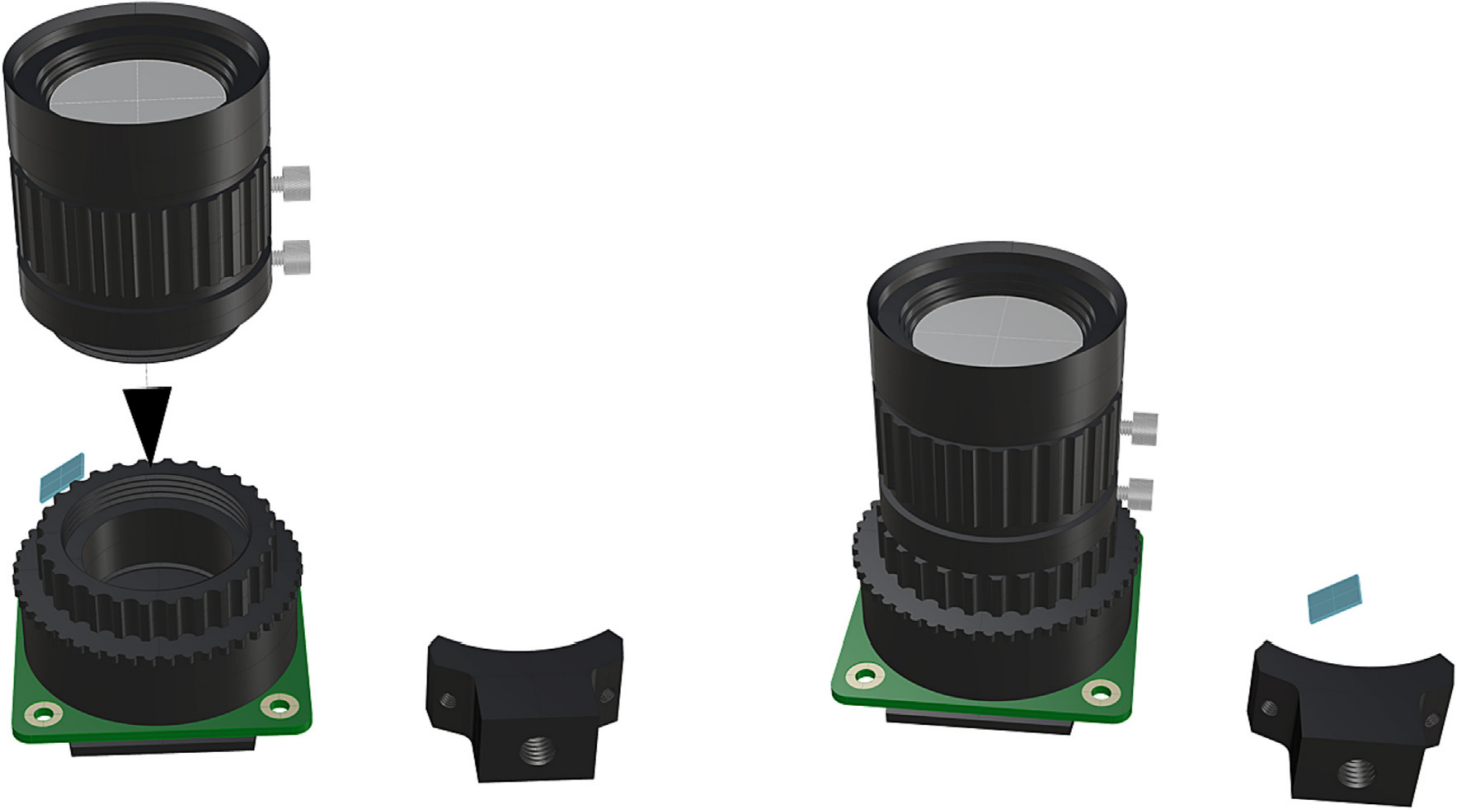
Installation of camera lense (**P21**) and fully assembled camera with removed components.

**Fig. 25:**
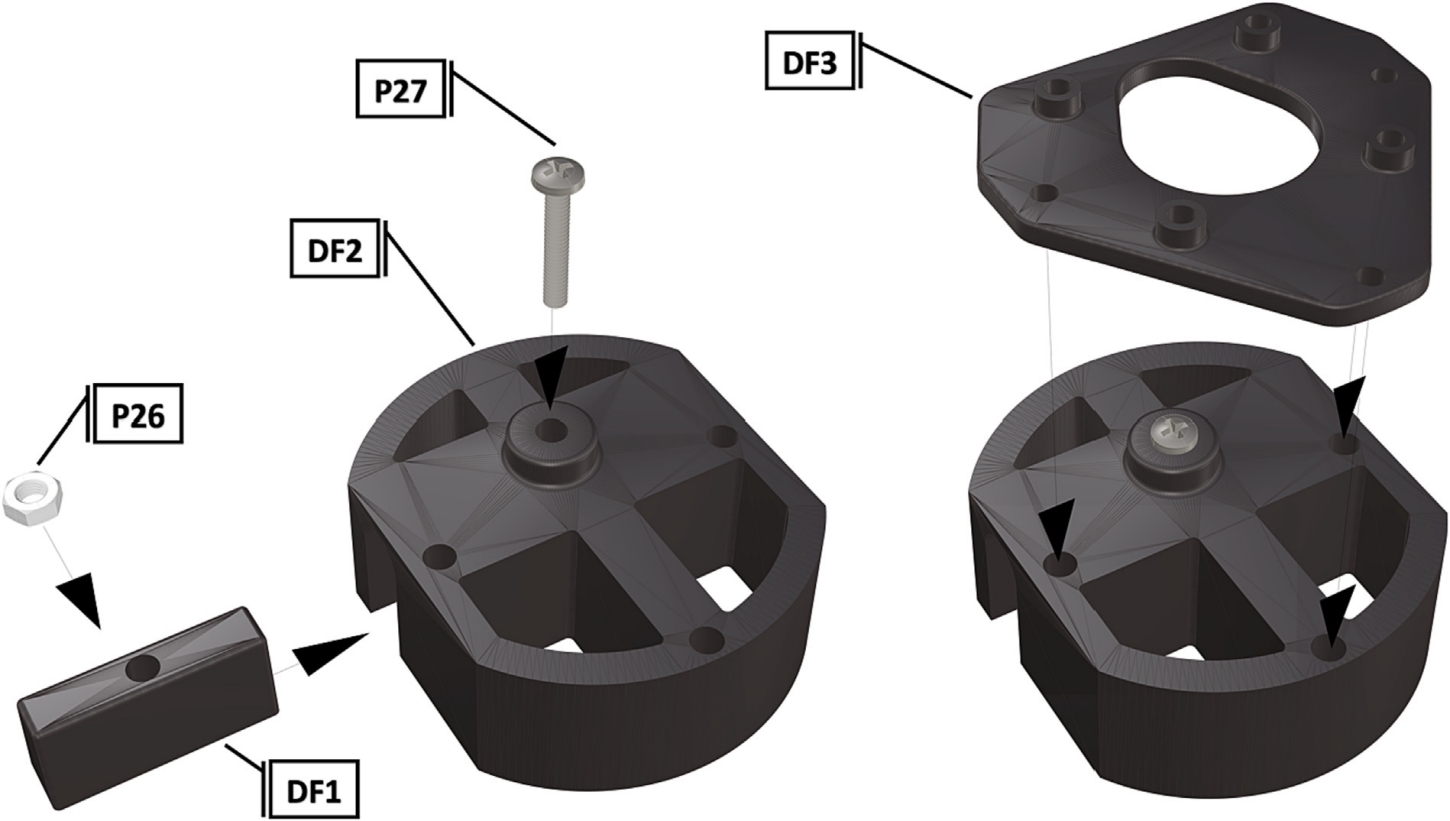
Port mount assembly and placement of camera mount attachment*.*

**Fig. 26:**
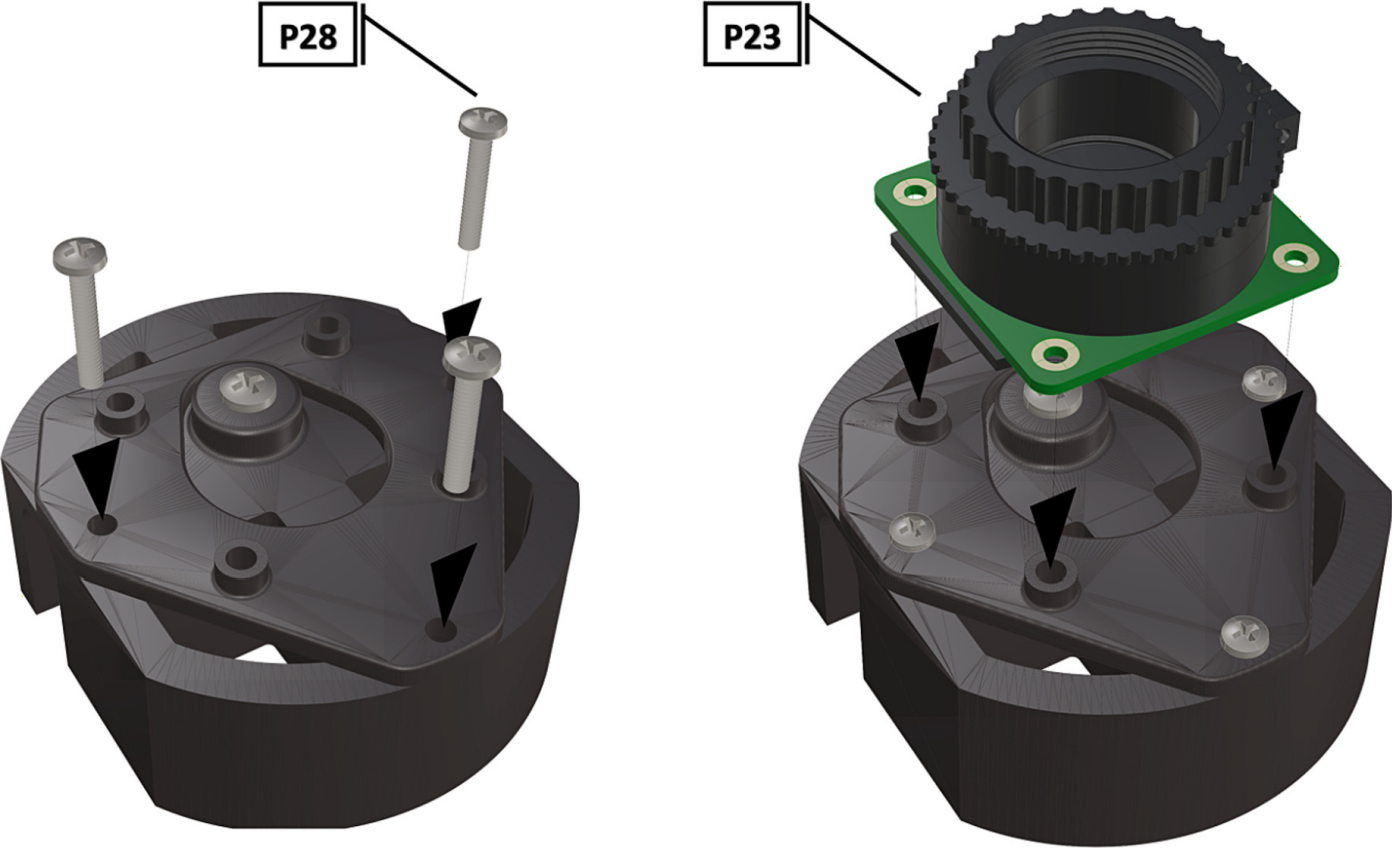
Bolts installed to hold camera mount in place, camera circuit board installation on manufactured camera mount.

**Fig. 27:**
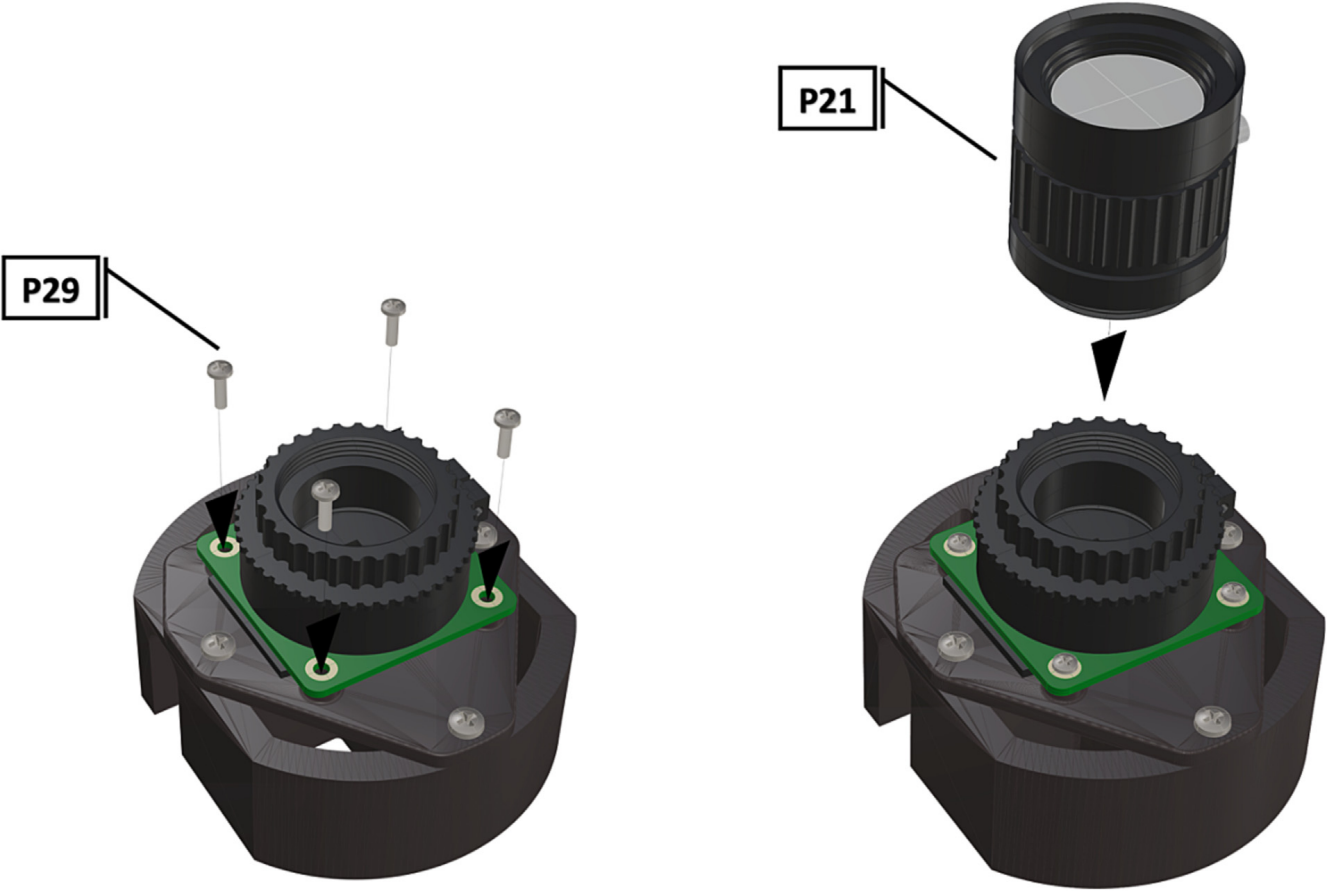
Bolts used to hold camera circuit board in place, wide angle CS-mount lens attached to Arducam base.

**Fig. 28:**
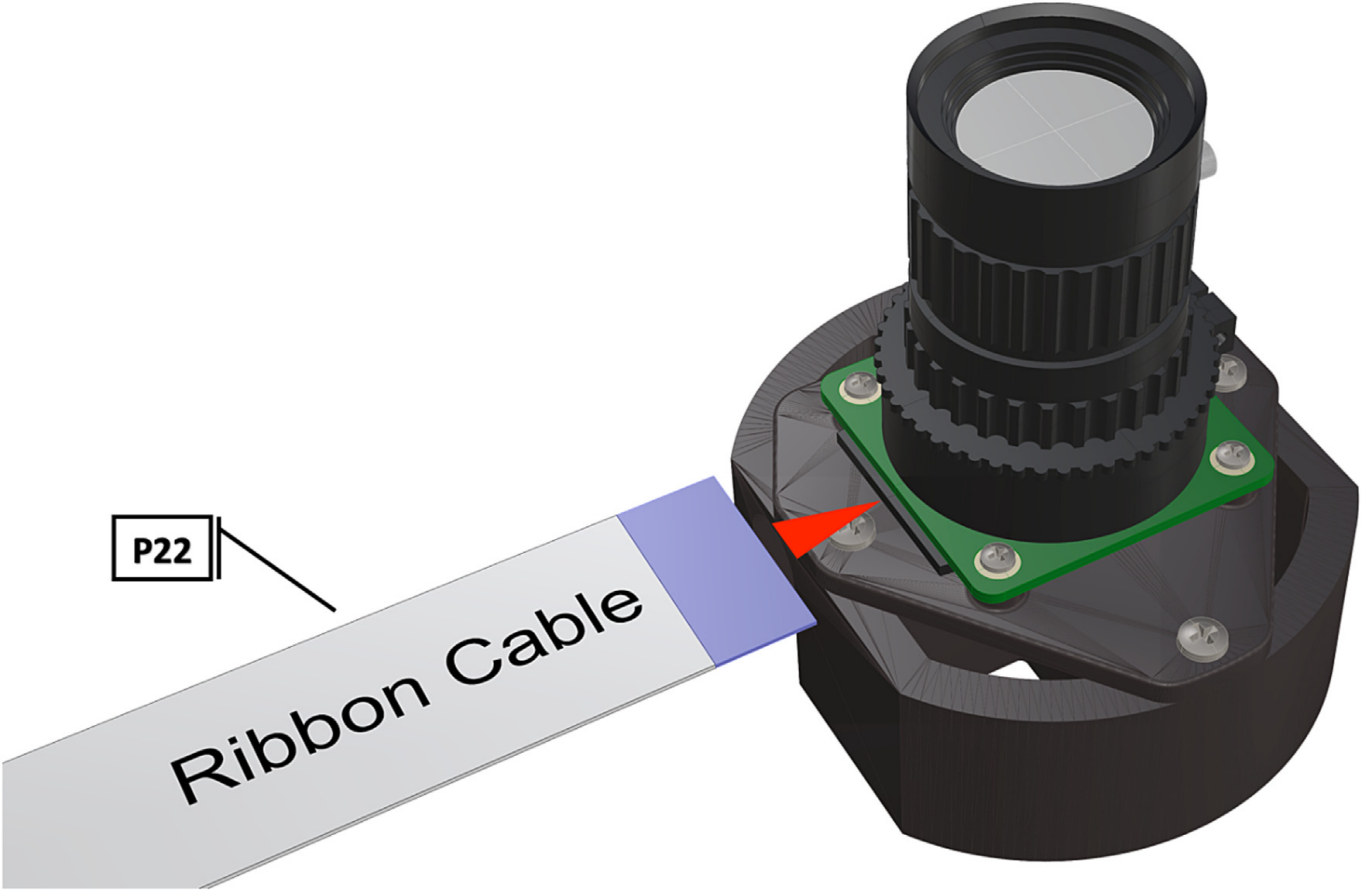
Installation of ribbon cable to CSI/DSI connector.

**Fig. 29:**
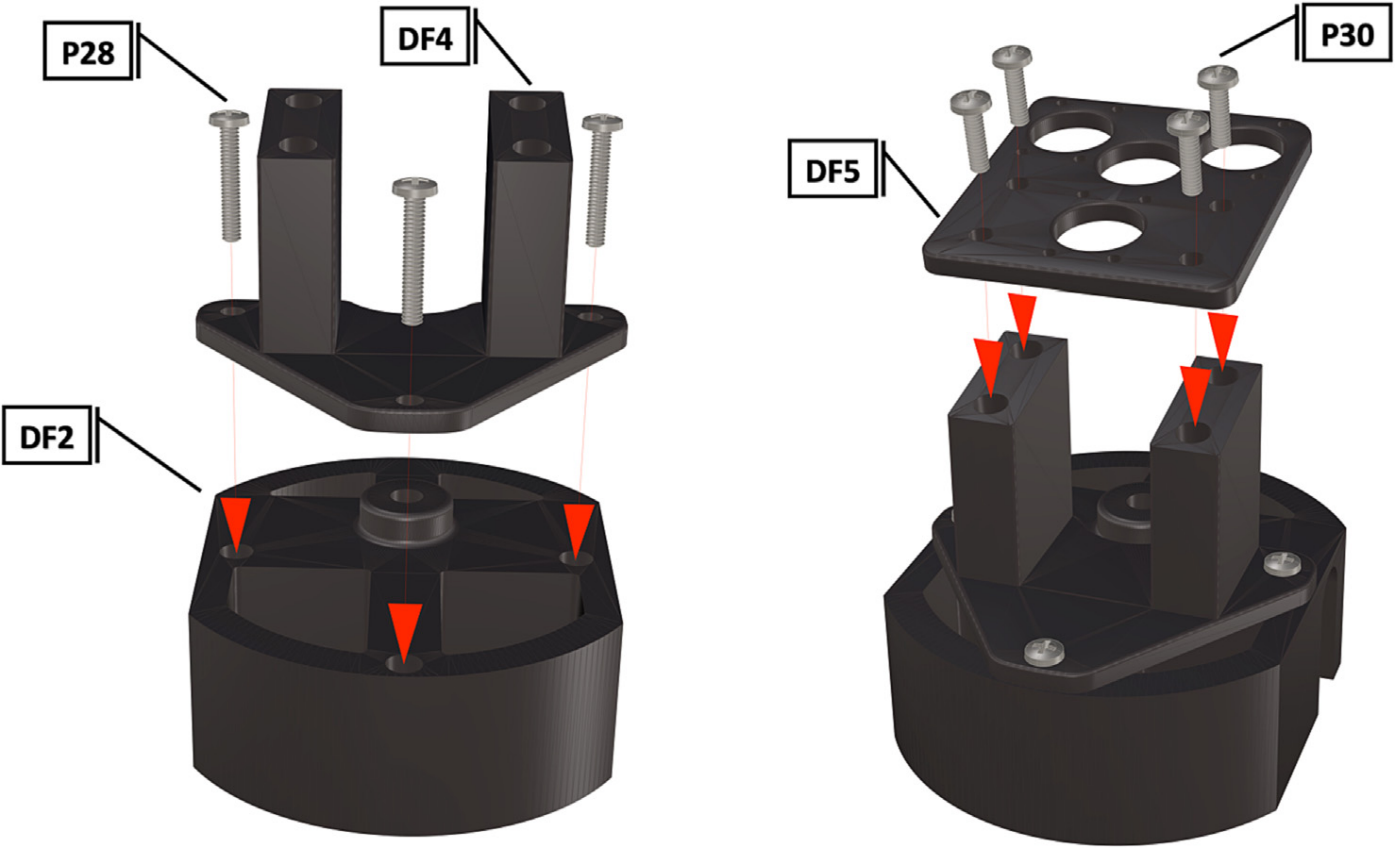
Installation of LED support frame on port mount, installation of LED starboard mounting plate.

**Fig. 30:**
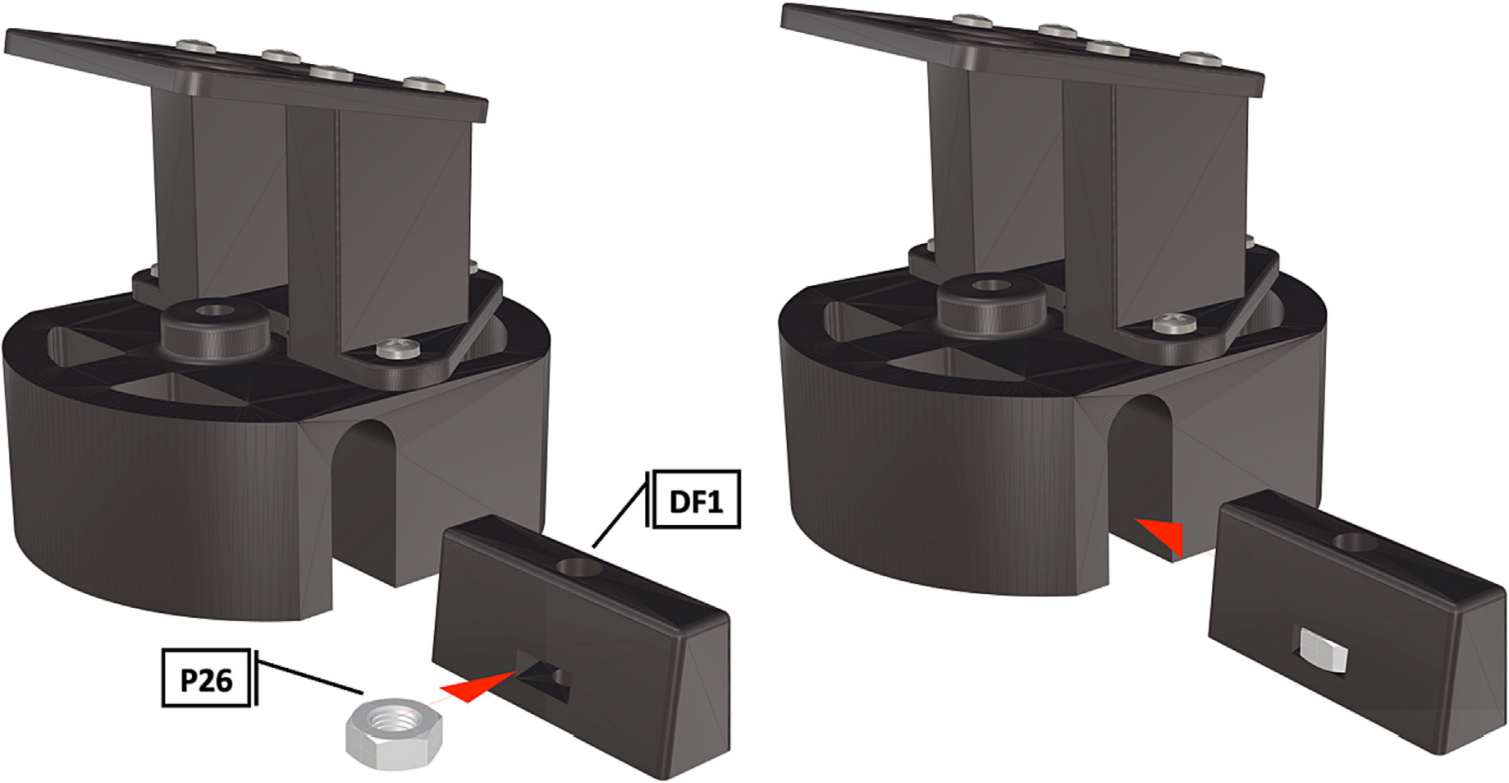
Insertion of nut into spreading wedge, spreading wedge inserted inside port mount.

**Fig. 31:**
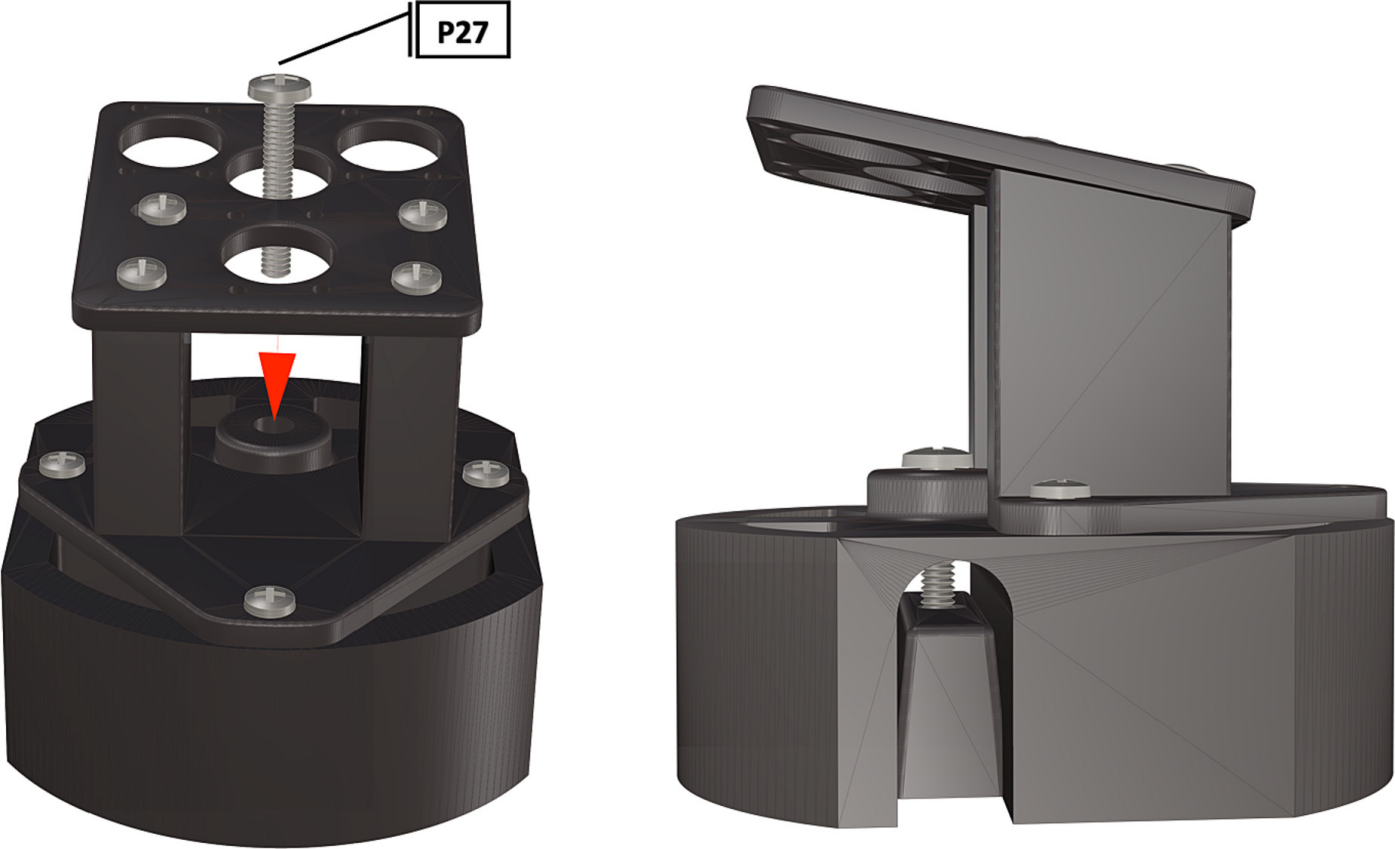
Bolt is threaded into spreading wedge, tightening of bolt draws wedge into opening and expands port mount diameter, creating a tight fit within a 3″ pipe.

**Fig. 32:**
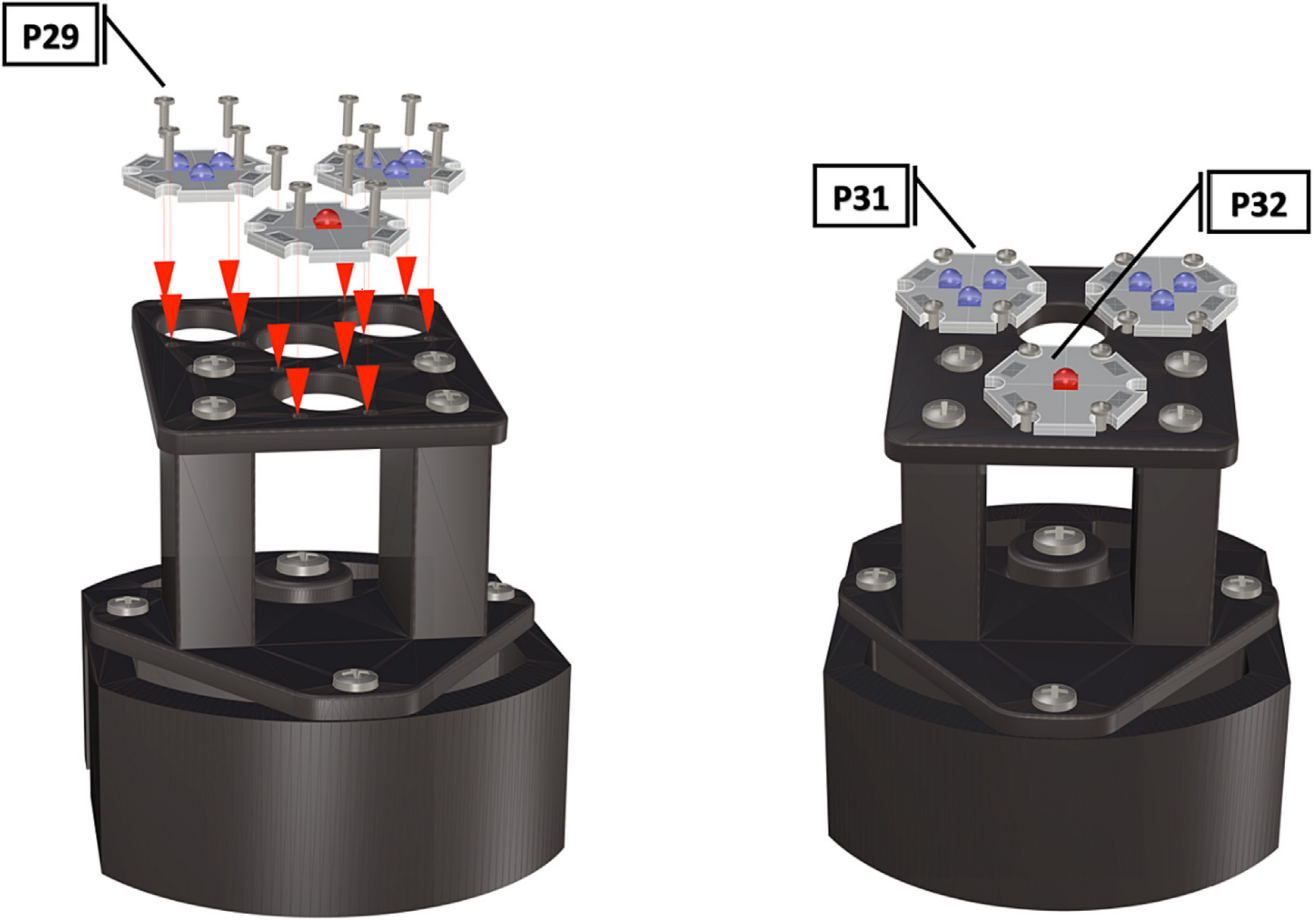
LED starboards secured with bolts and final placement of LED starboards.

**Fig. 33:**
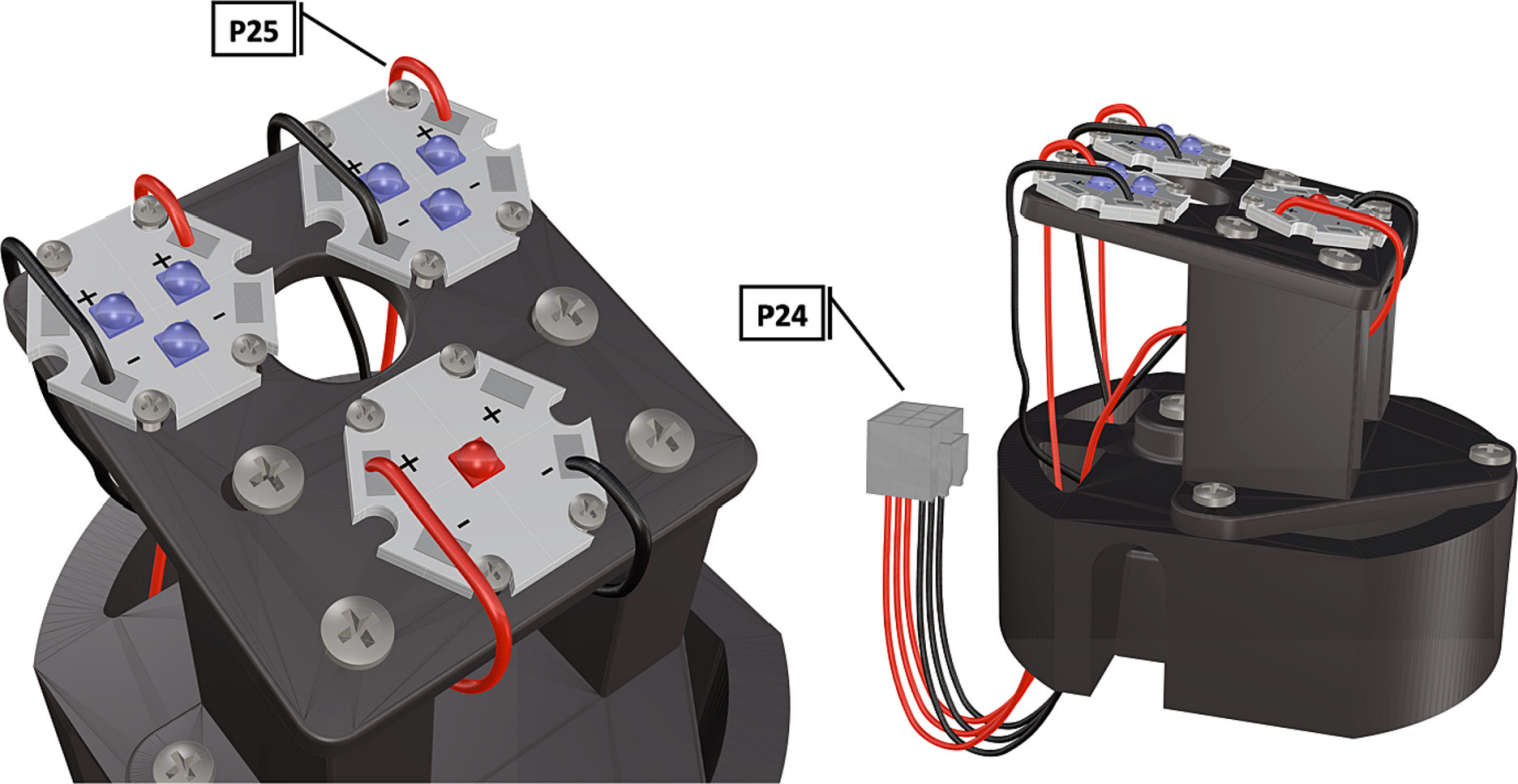
Wiring diagram for LED starboards, wires are soldered in place. Wiring is installed in a female Molex connector for direct attachment to camera strobe system (Fig. 18).

**Fig. 34:**
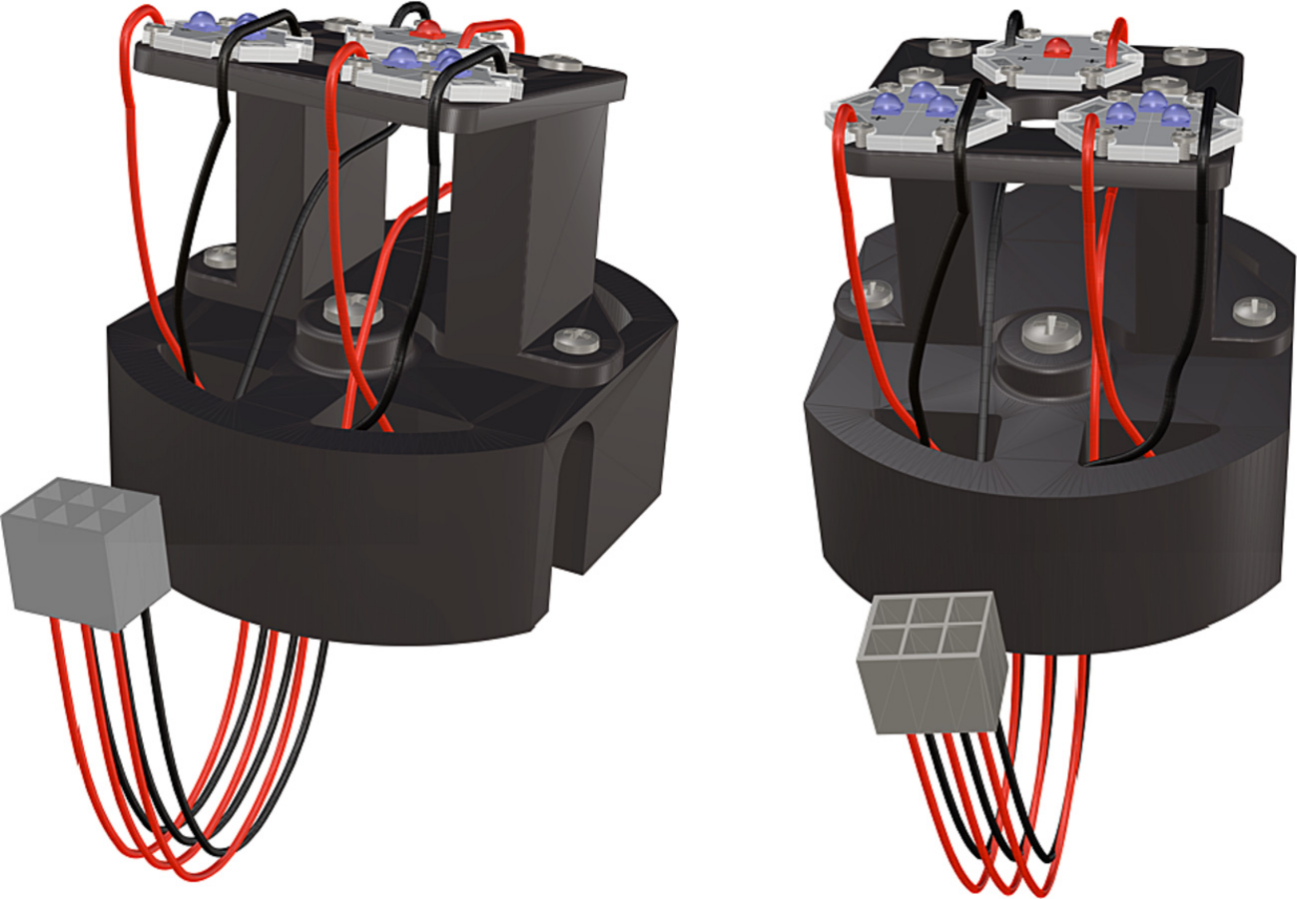
Completed LED system with wiring harness and attached LED starboards.

**Fig. 35:**
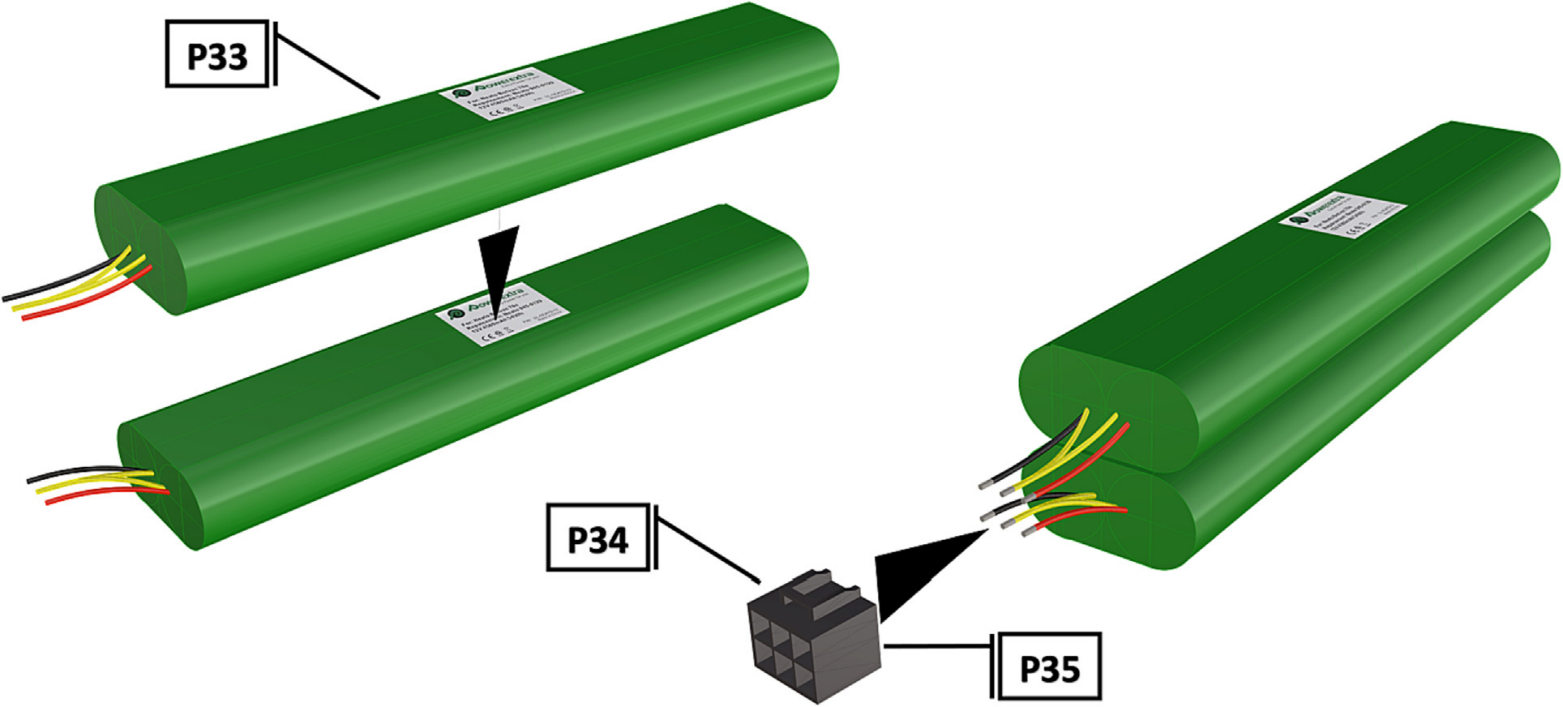
Battery packs oriented above each other; battery array is wired together into a female Molex connector.

**Fig. 36:**
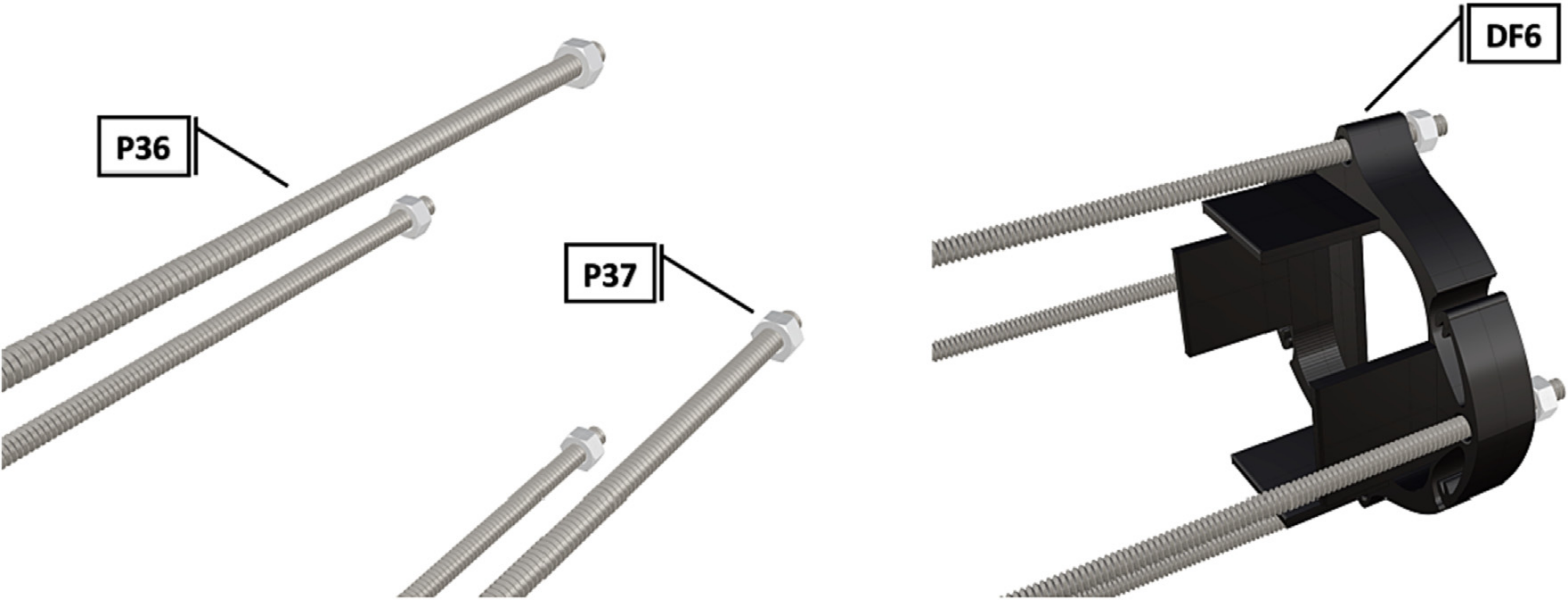
Threaded rods with attached nuts, battery holder end cap placed against bolt nuts.

**Fig. 37:**
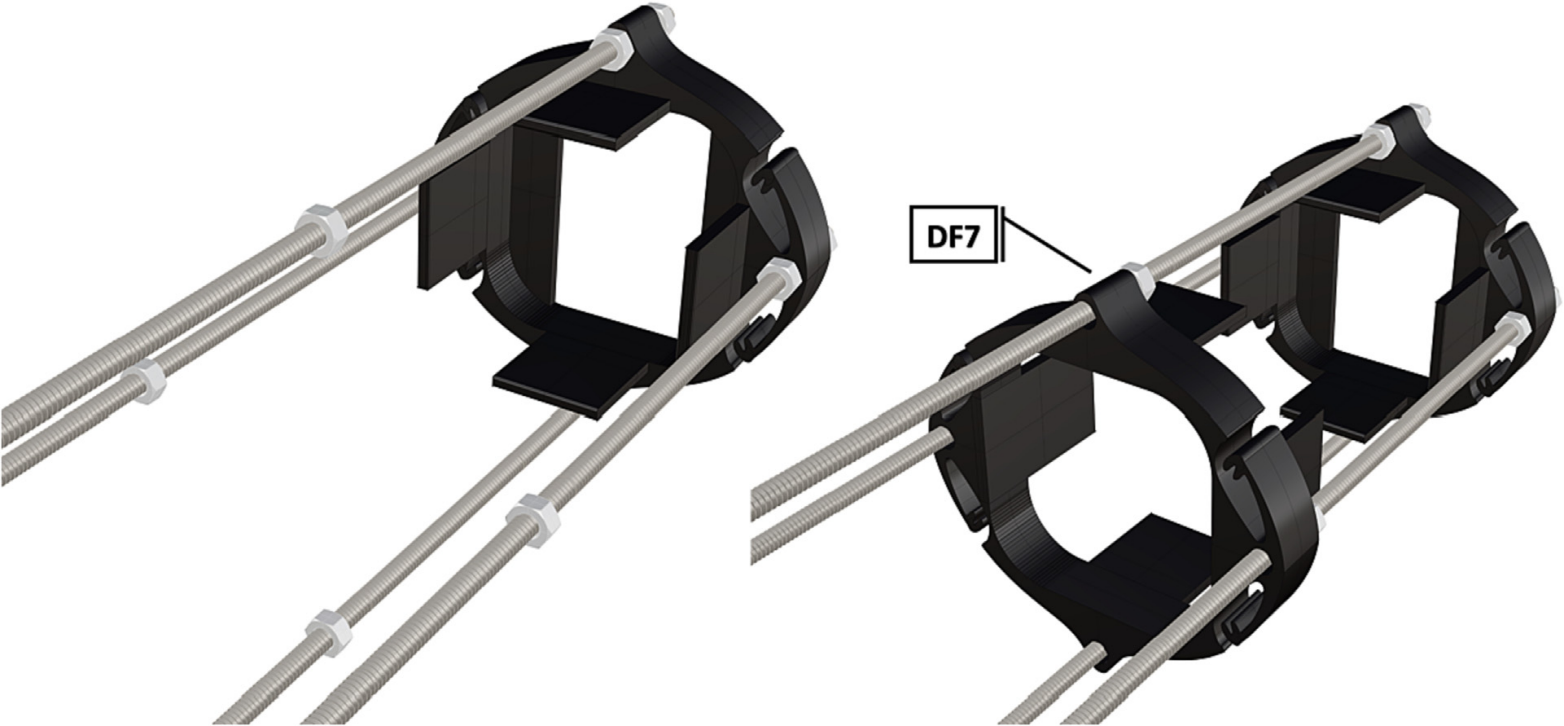
Attached nuts are used to hold a battery support in place.

**Fig. 38:**
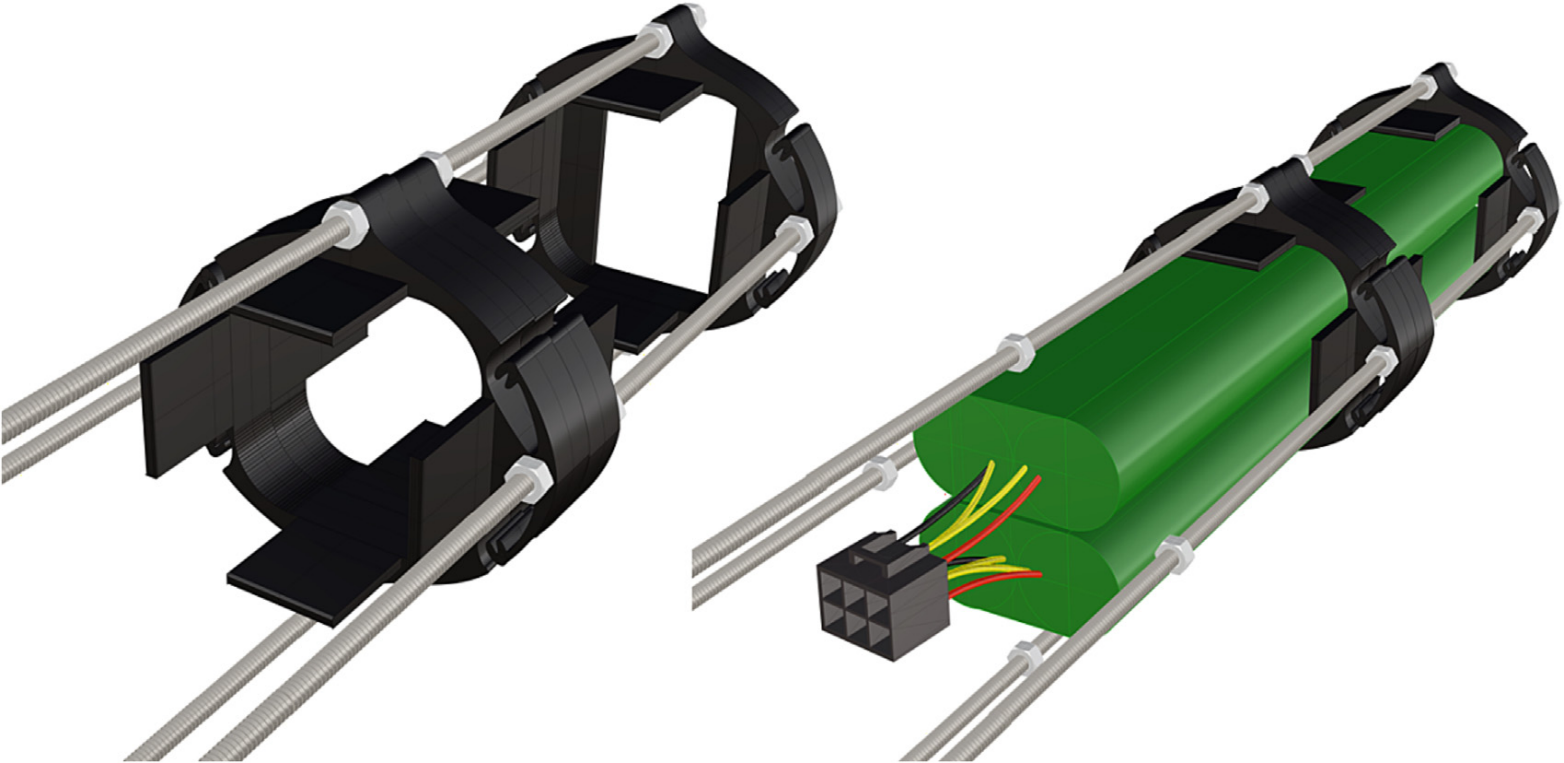
The addition of a second opposing battery holder adds additional support, followed by the tightening of attached nuts.

**Fig. 39:**
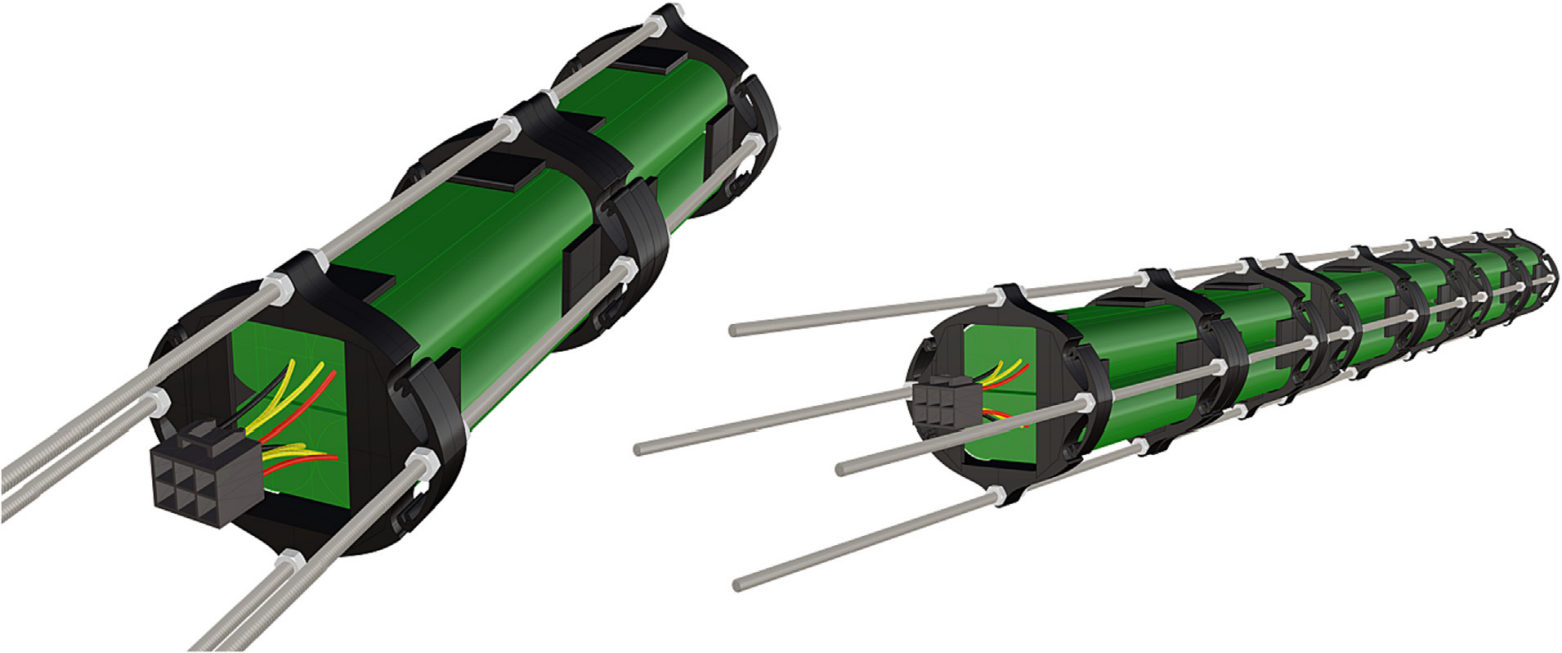
Complete assembly of one battery unit, followed by 2 more identical battery packs following the same assembly instructions and attached together on threaded metal rods (**P36**).

**Fig. 40:**
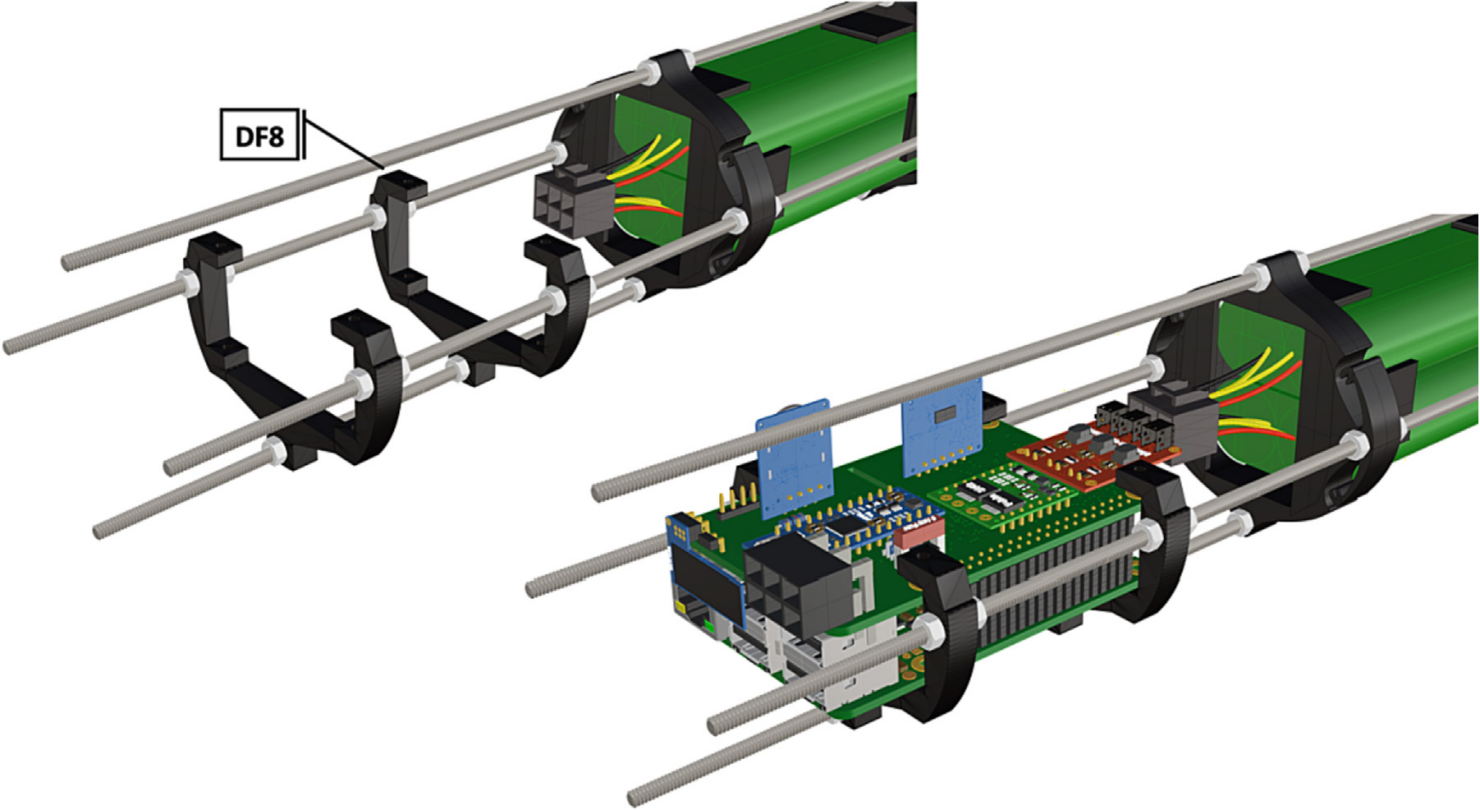
PCB mounts attached in the same manner as battery holders.

**Fig. 41:**
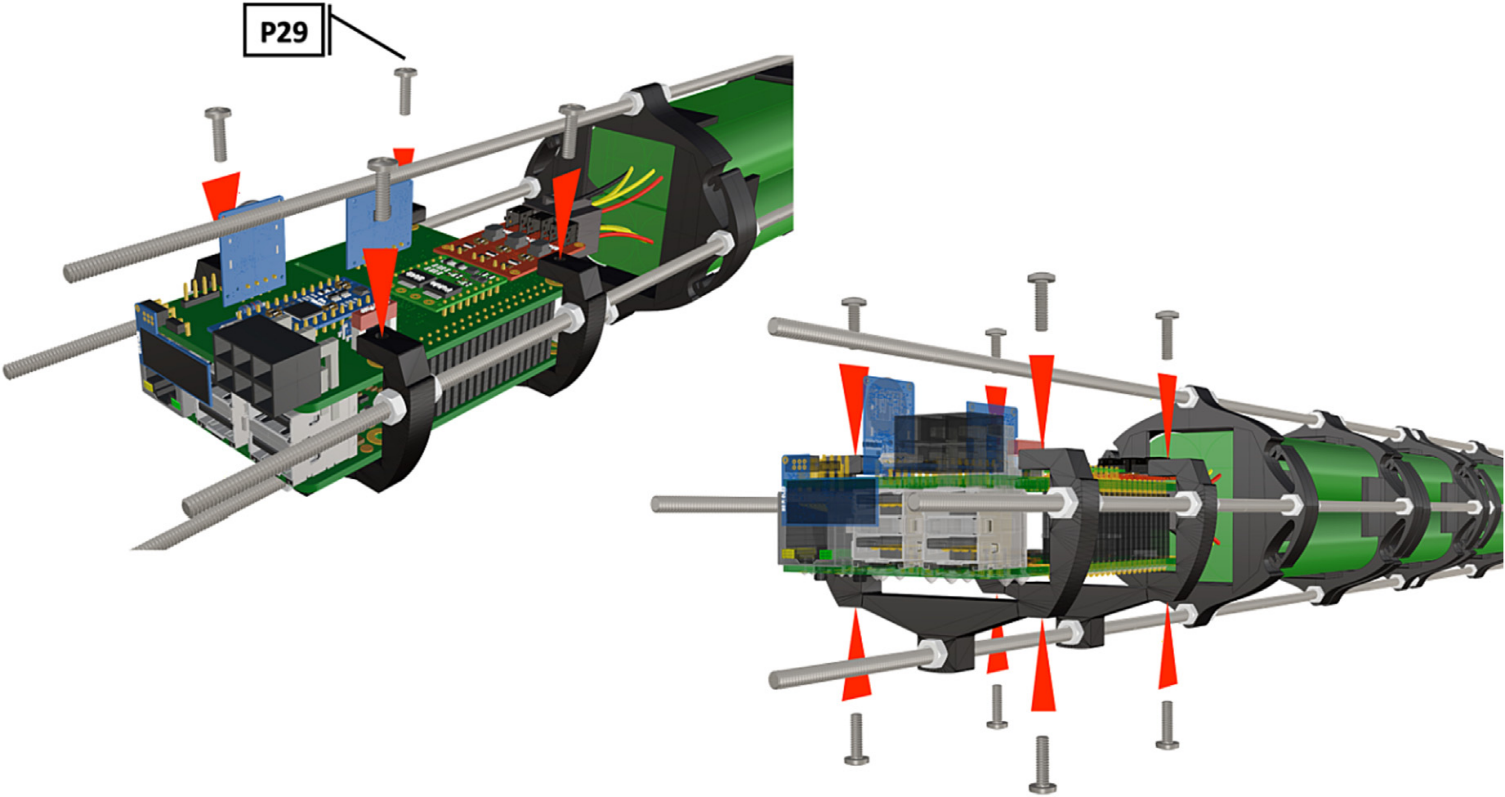
Bolts are inserted through attachment points to keep PCB in position.

**Fig. 42:**
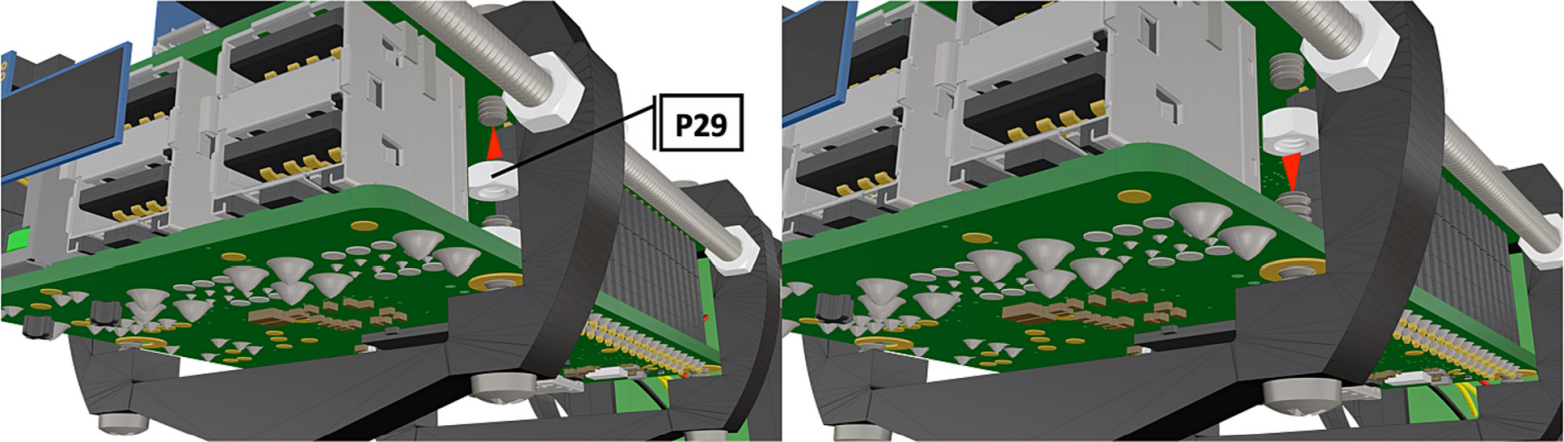
Nuts are attached to all 8 bolts inserted into PCB mount (Fig. 41) and tightened to secure PCB hat and Raspberry Pi3B+  in place.

**Fig. 43:**
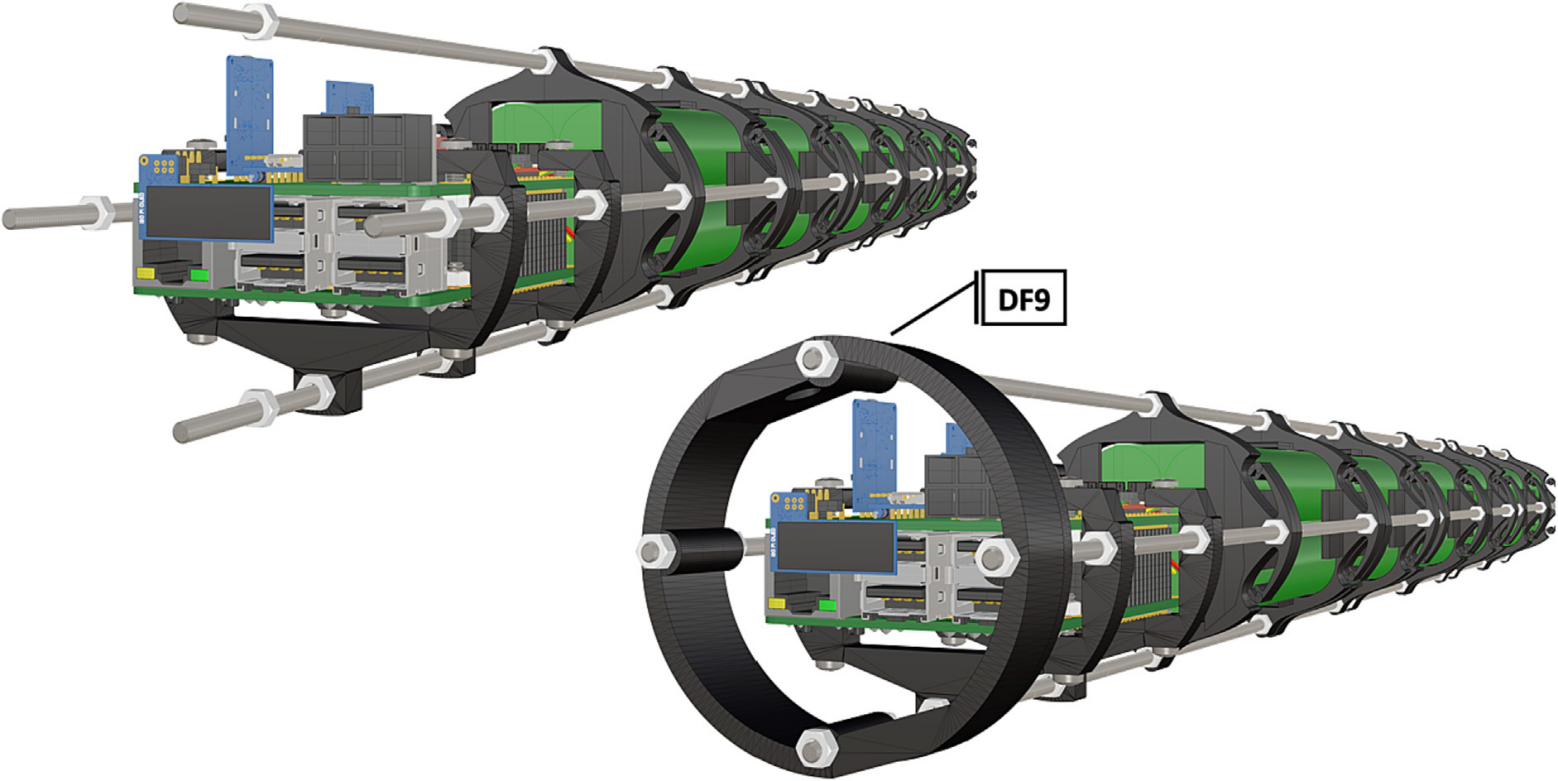
After computer components and battery packs are assembled, a front bulkhead is attached to aid in structural support.

**Fig. 44:**
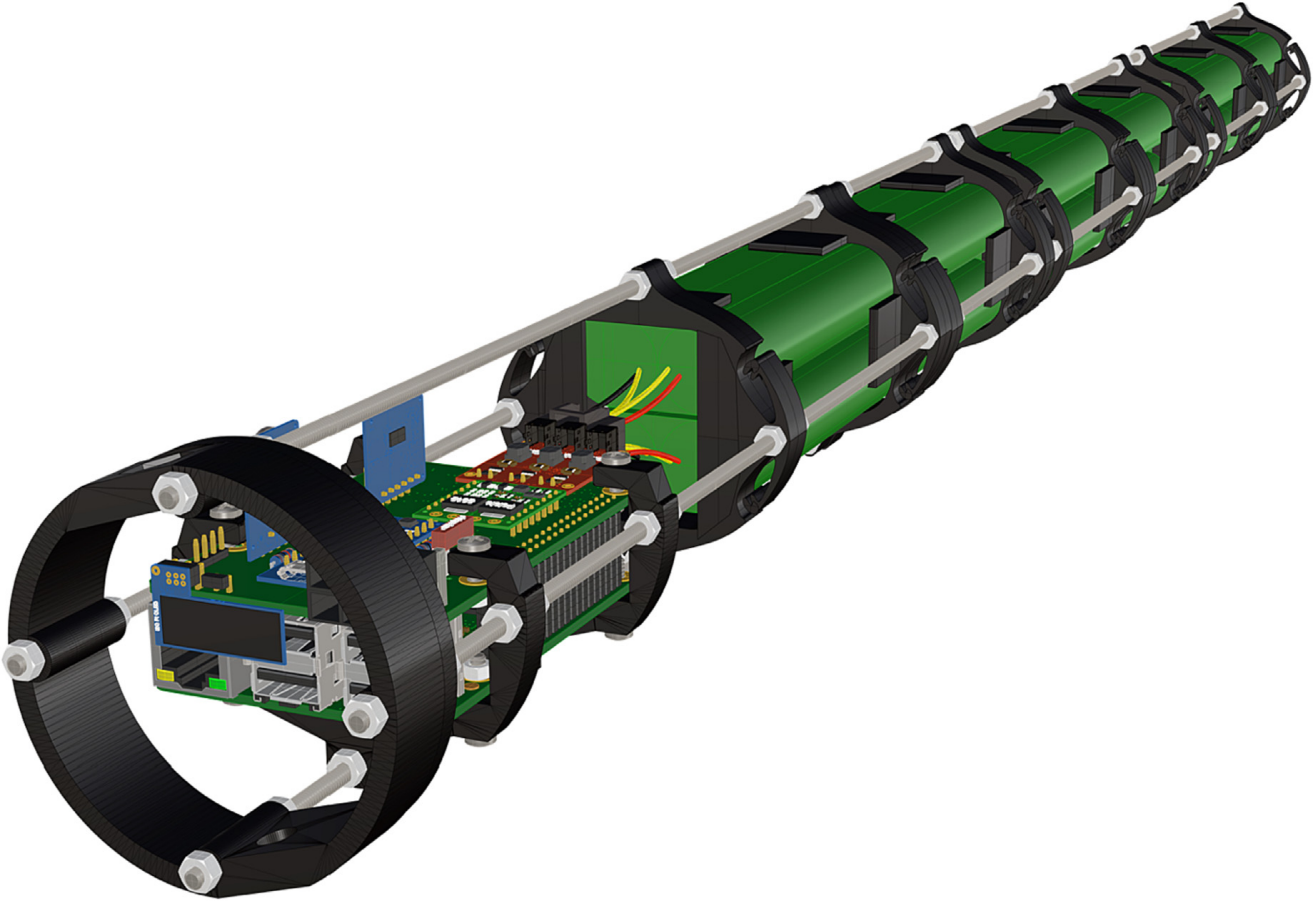
Completed structural assembly.

**Fig. 45:**
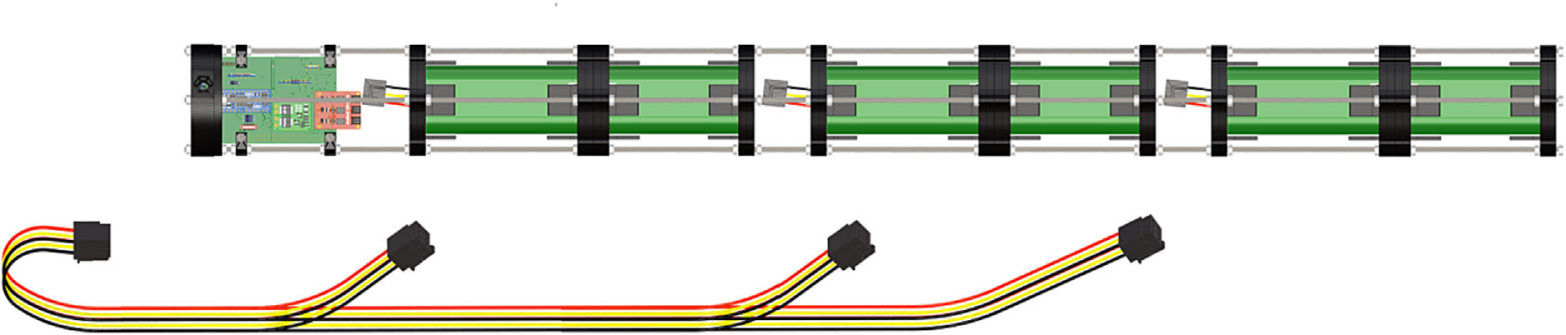
Diagram of wiring harness, adjacent to designated connectors.

**Fig. 46:**
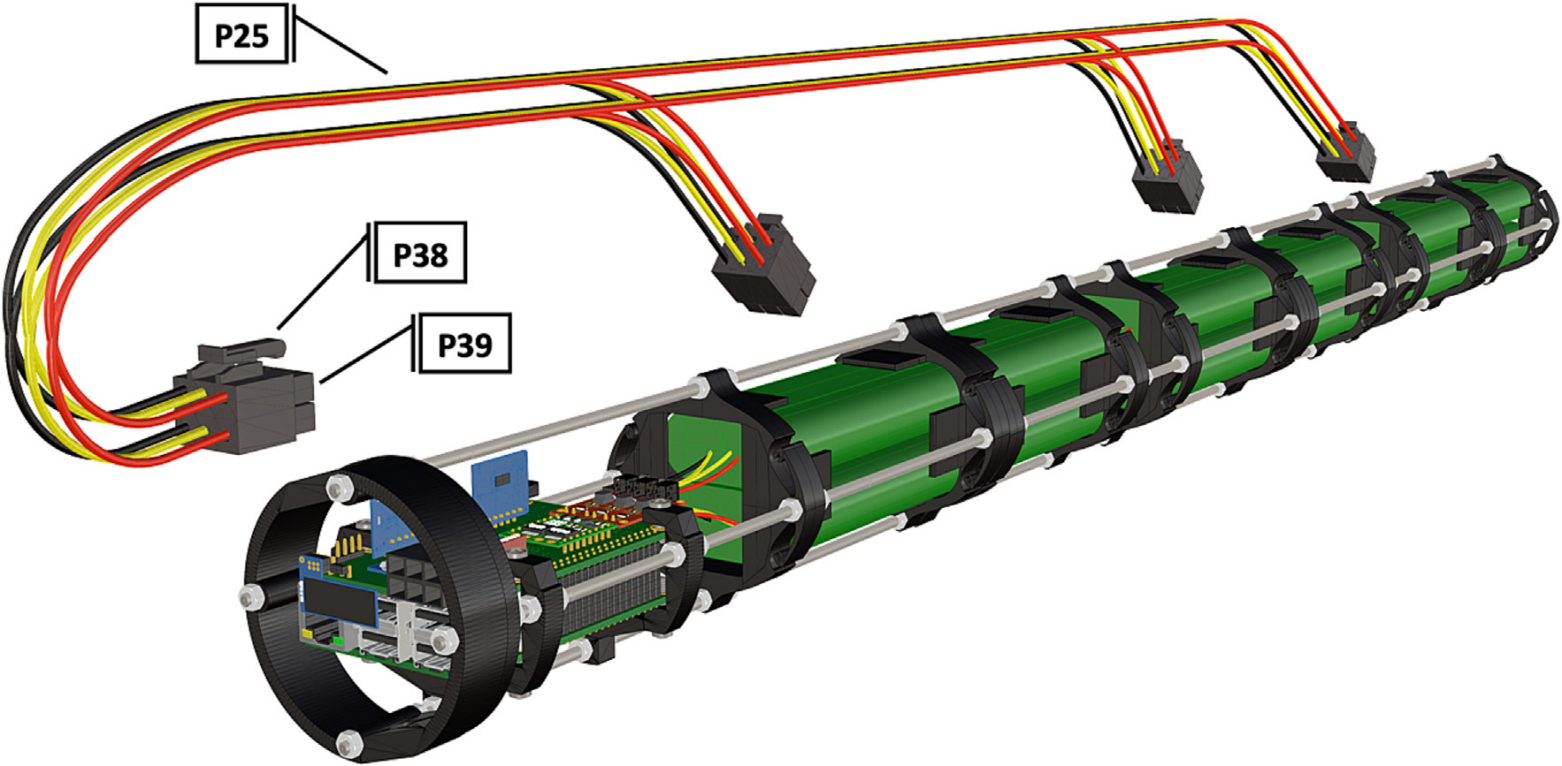
Wiring harness, plugs connect to female receptacles on battery packs and PCB.

**Fig. 47:**
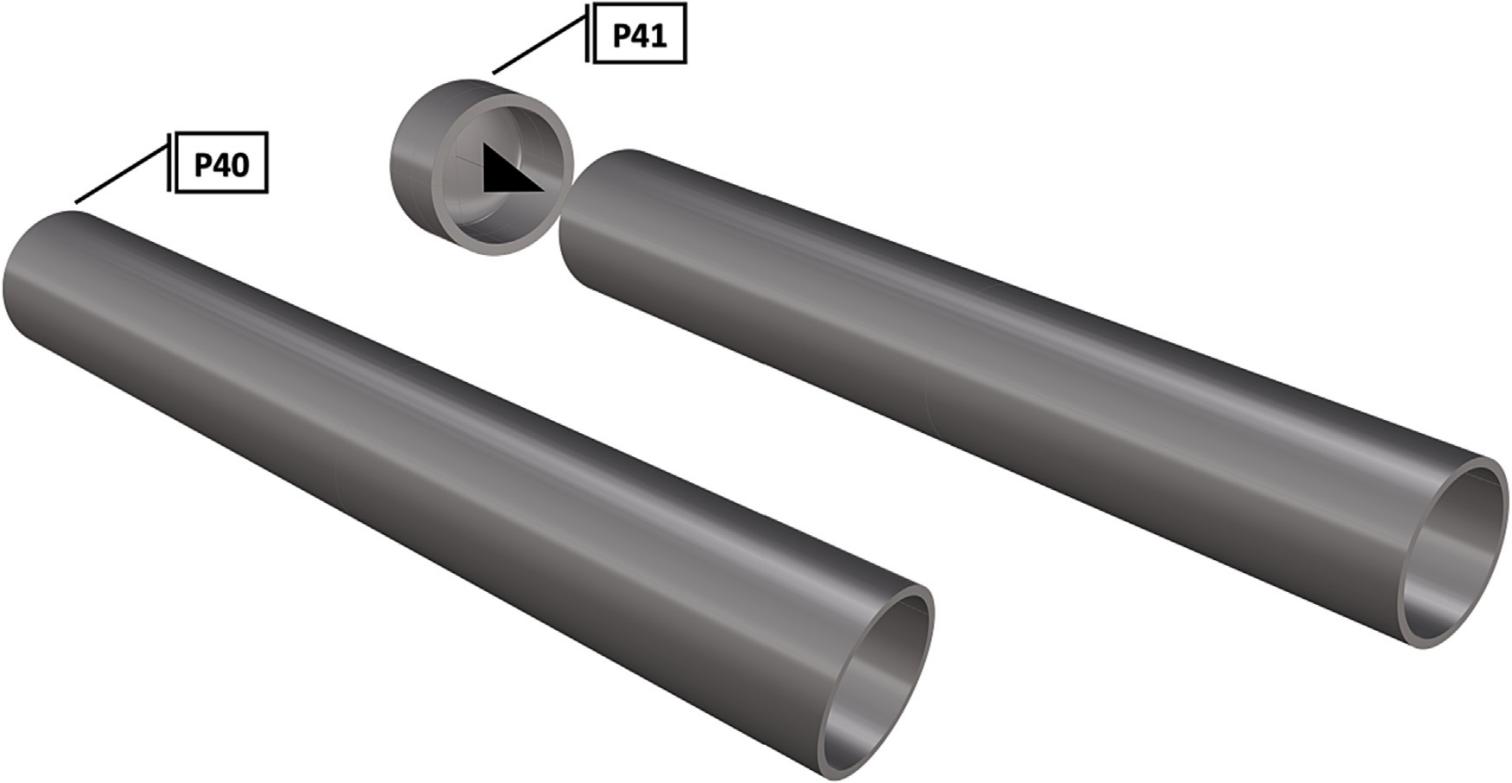
Cut to size schedule 80 PVC pipe with attached pipe cap.

**Fig. 48:**
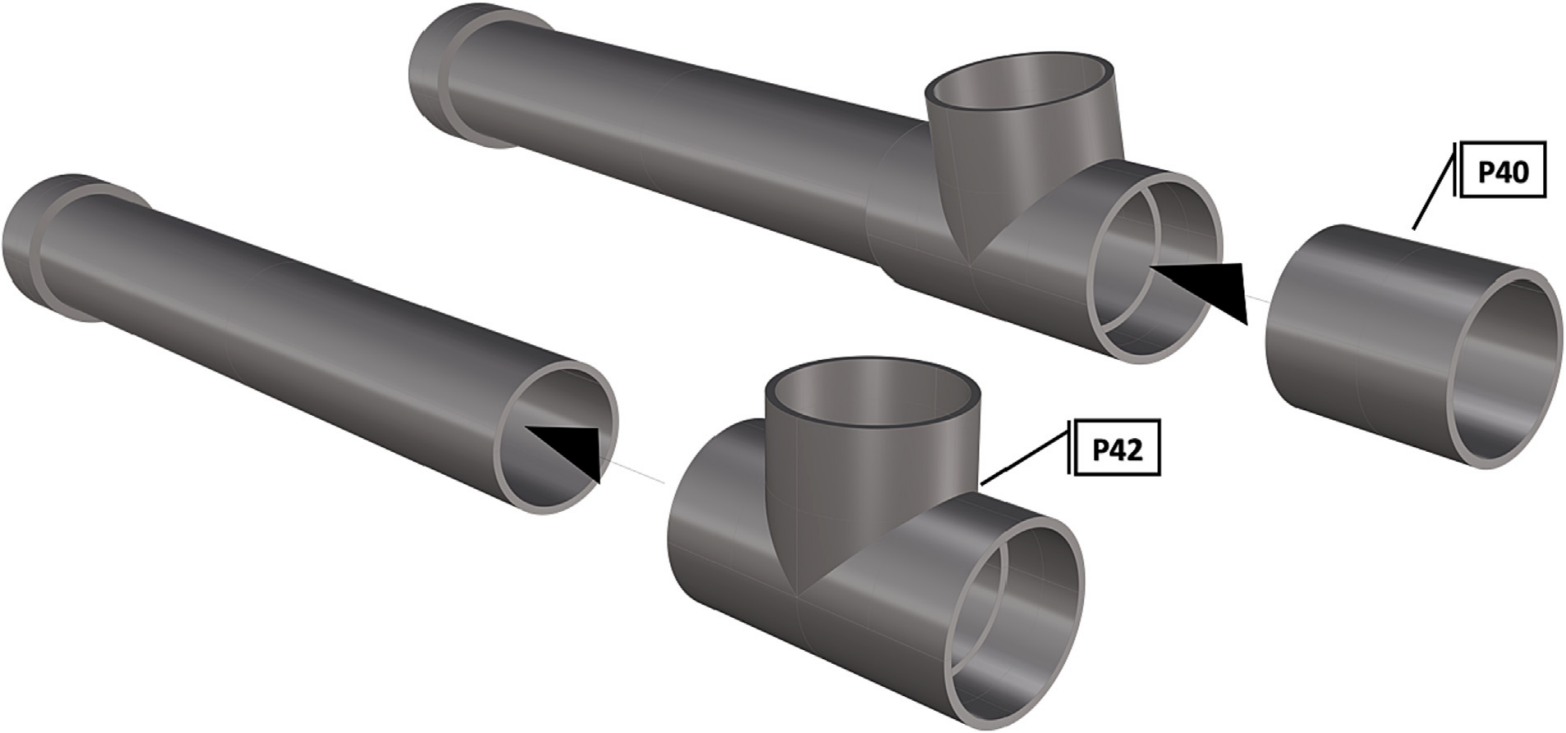
Installation of *T*-joint, followed by a 6 in. section of pipe (**P40**).

**Fig. 49:**
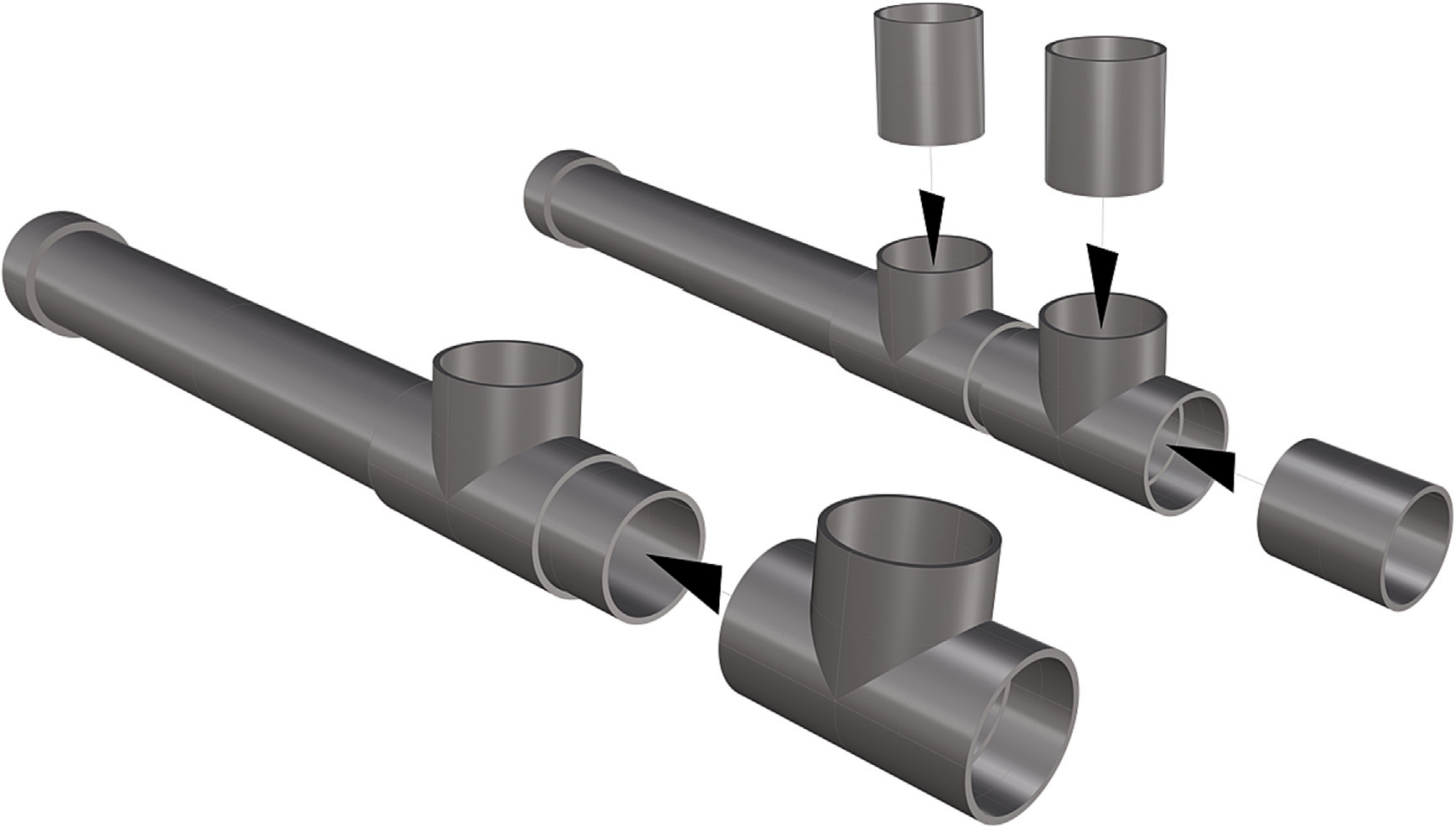
Attachment of second *T*-joint and three cut sections of 6 in. pipe.

**Fig. 50:**
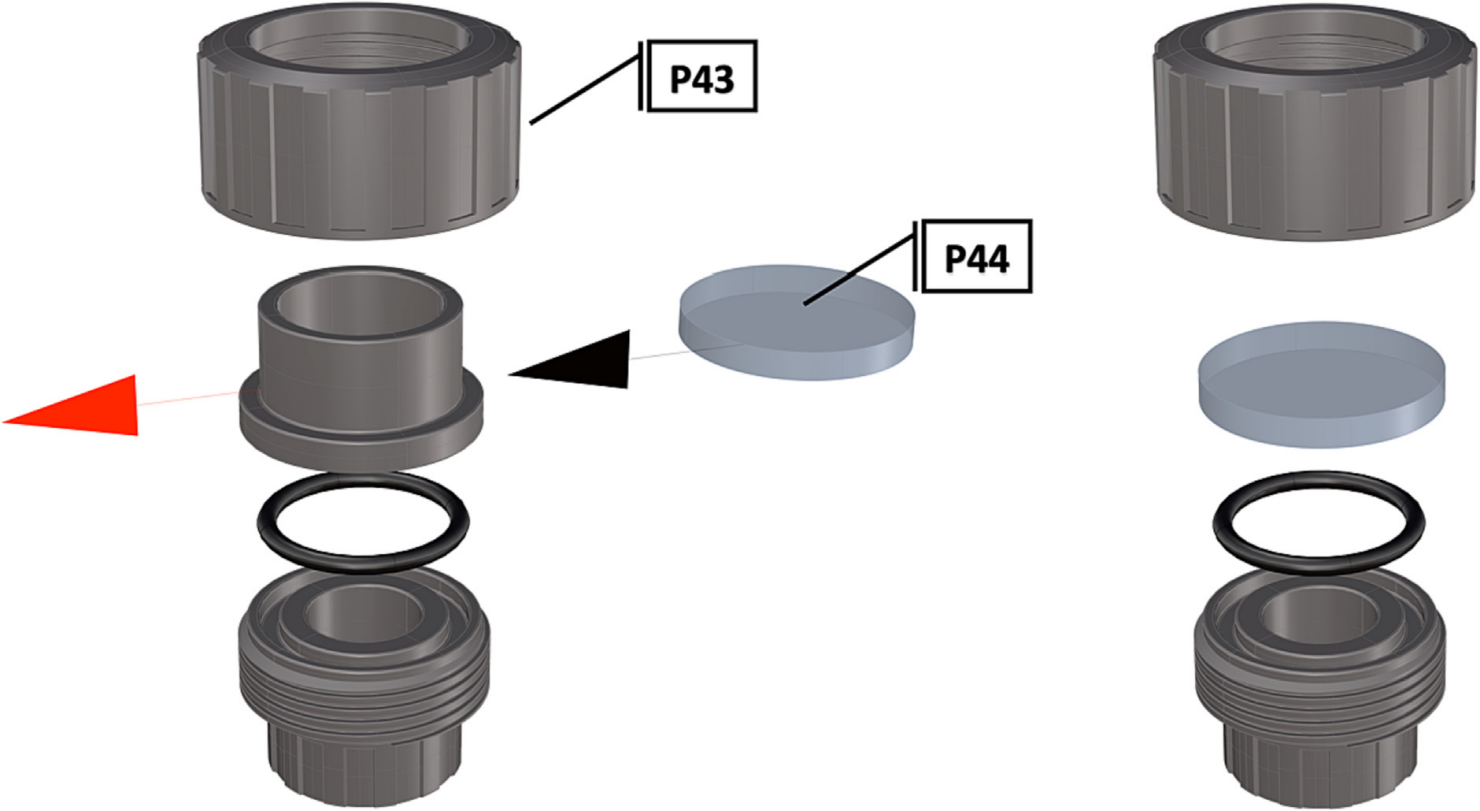
Removal of female Union sleeve to allow installation of Plexiglass or glass port cover.

**Fig. 51:**
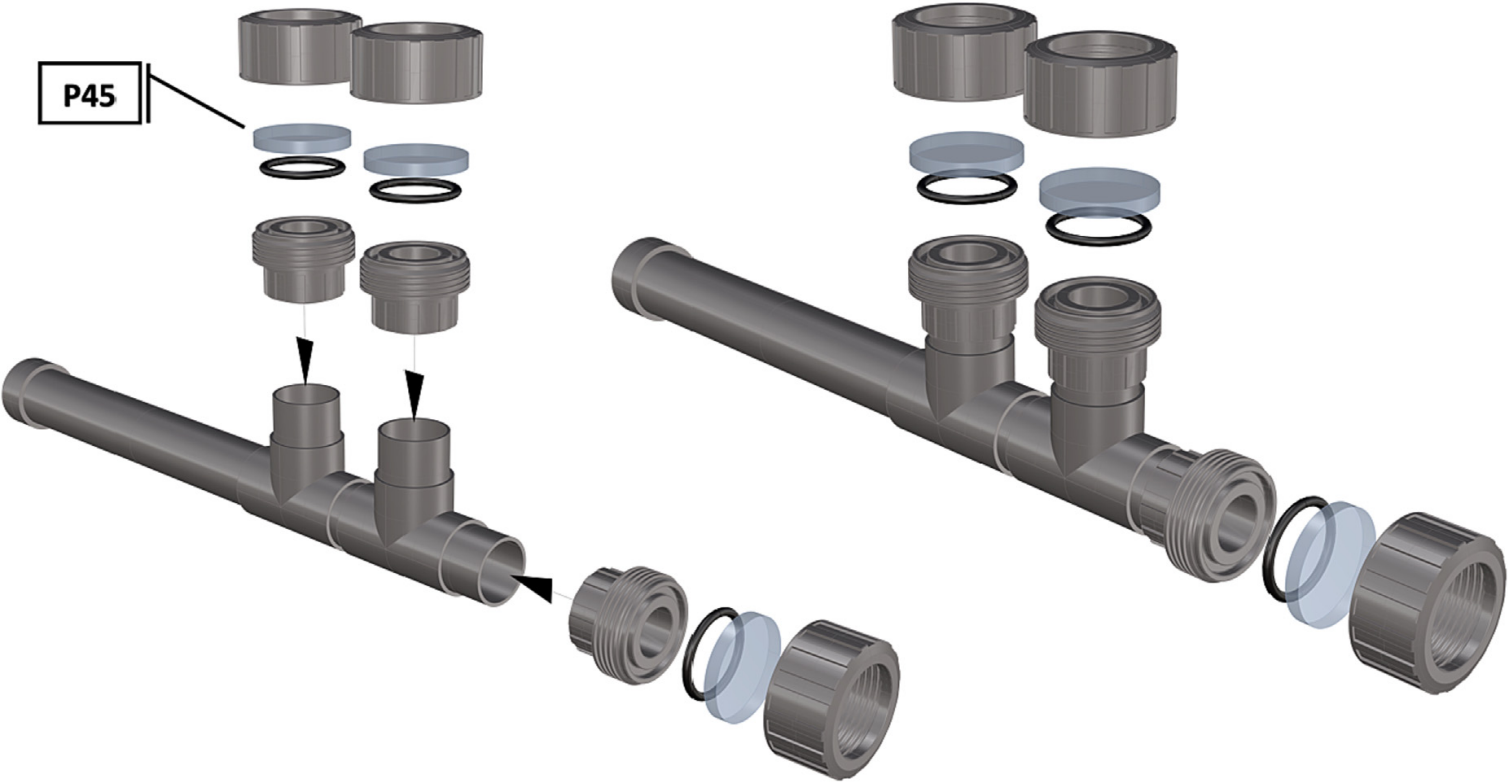
Installation of Union joints to 6 in. pipe sections, forming the ports and port covers.

**Fig. 52:**
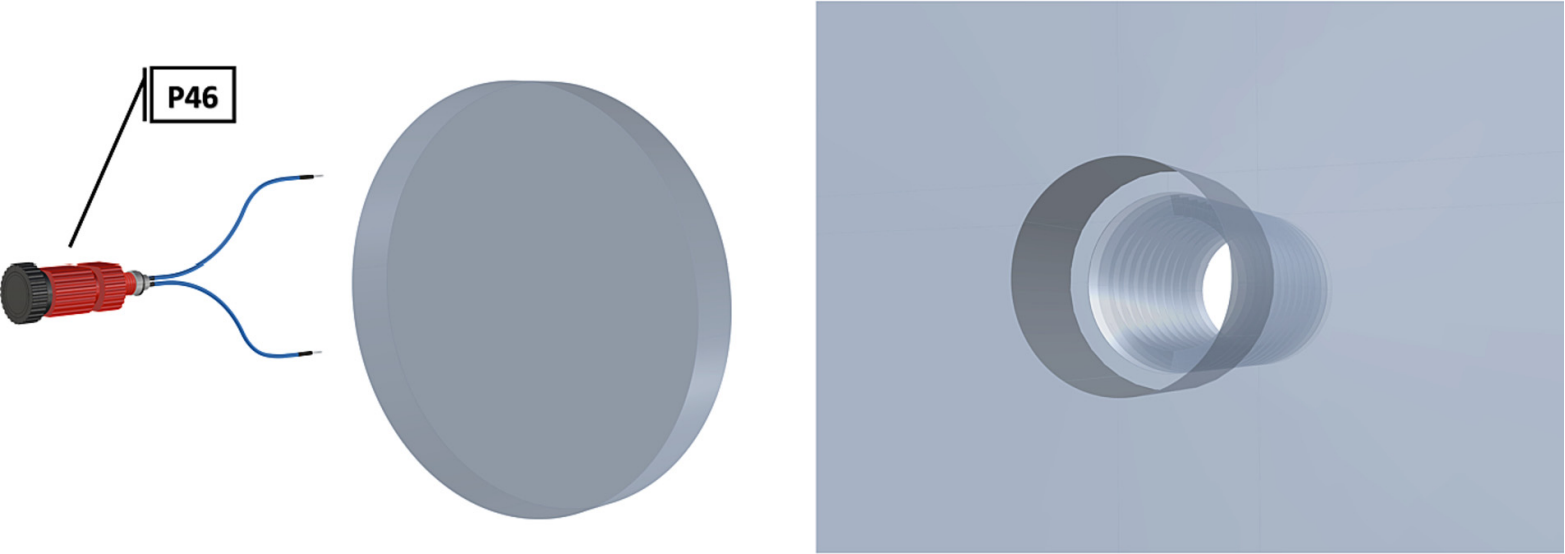
Drilling and tapping of ½” Plexiglass hole for installation of Blue Robotics switch. (For interpretation of the references to color in this figure legend, the reader is referred to the web version of this article.)

**Fig. 53:**
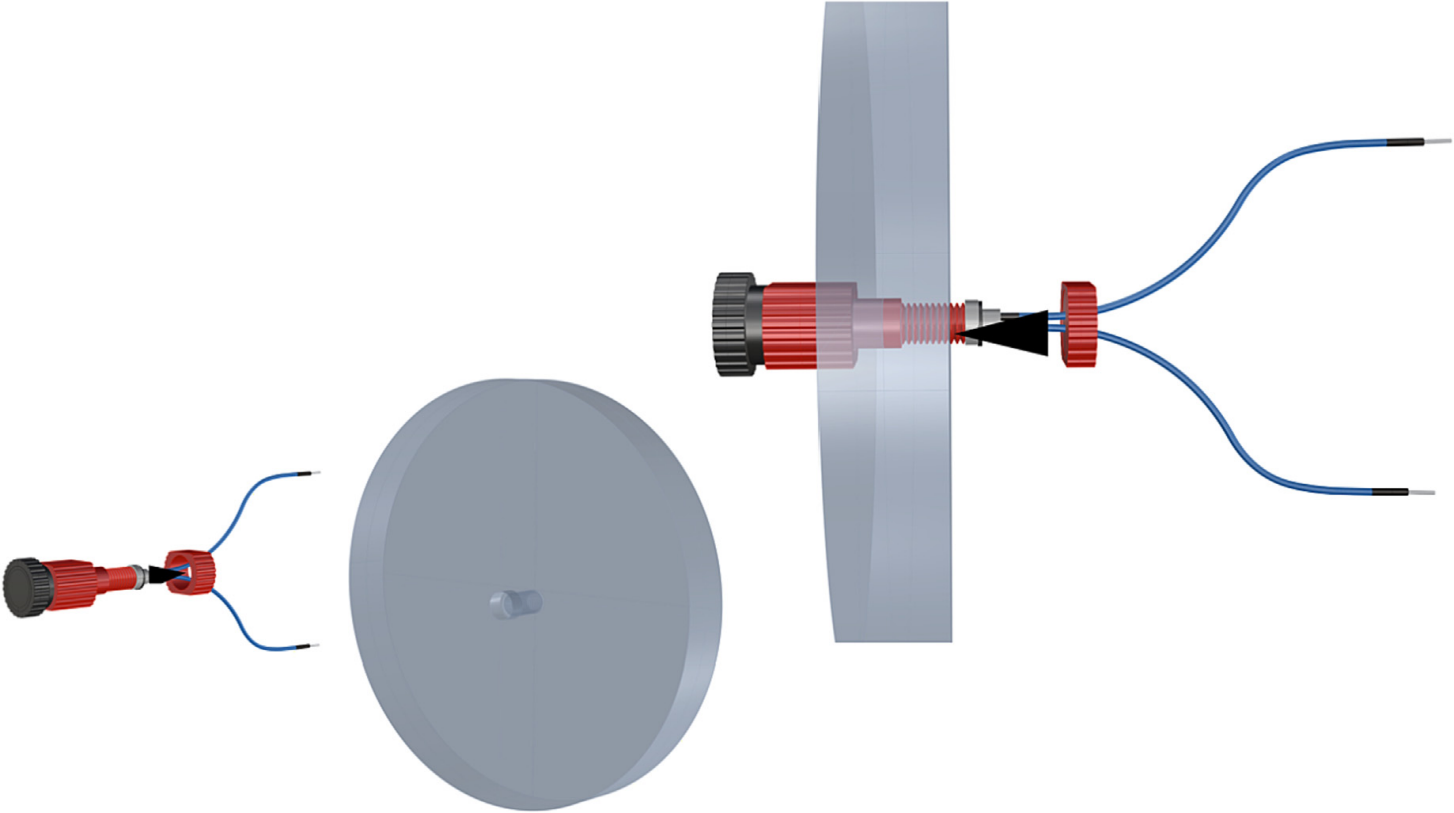
Mounting of Blue robotics switch in prepared hole. (For interpretation of the references to color in this figure legend, the reader is referred to the web version of this article.)

**Fig. 54:**
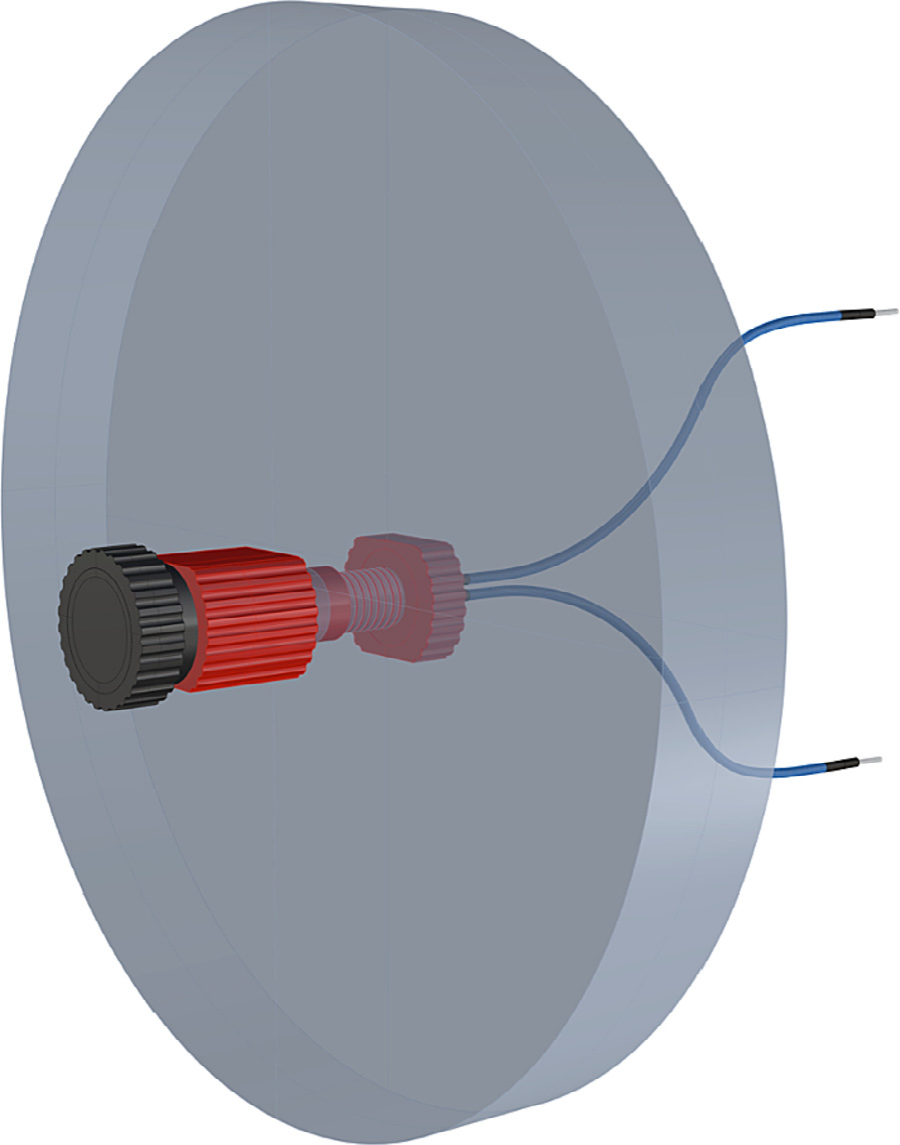
Completed switch installation in plexiglass port cover, allowing system to be powered on at depth.

**Fig. 55:**
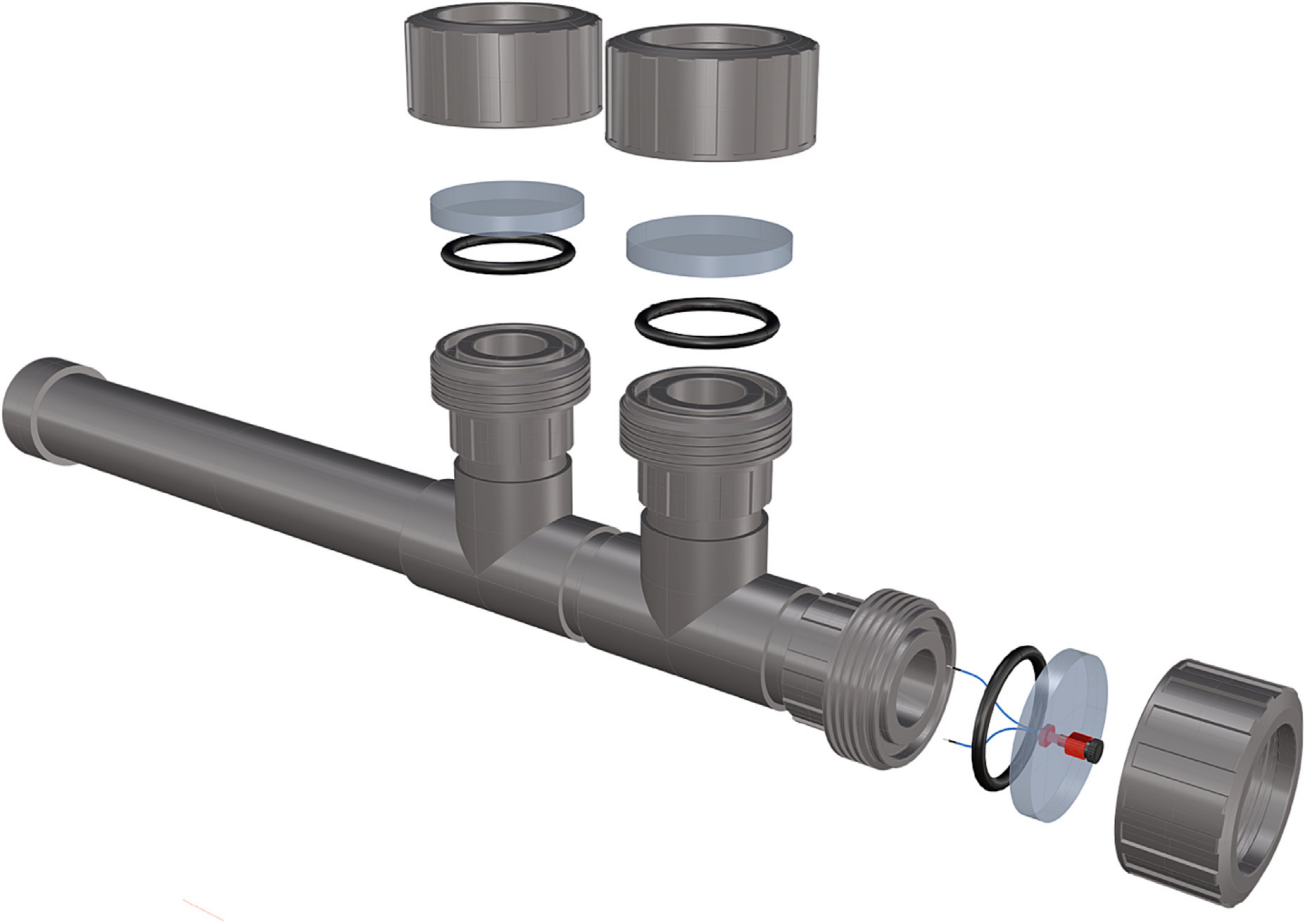
Completed schedule 80 PVC housing with exploded ports.

### 5.1 PCB assembly instructions

1. Insert 10 K resistors (**P2**) into through hole on PCB and solder to board (Fig. 5), trim any excess resistor leads after soldering. All soldering done during construction was performed using 0.6 mm solder wire (**P52**).
2. Solder fuse clip (**P4**) pins to the board before inserting the 5 Amp fuse (**P3**), Fig. 6.
3. Solder 4 pin male header (**P5**) to board for use as I2C bus Fig. 6.
4. Solder two pin header (**P6**) to board for attachment of Blue Robotics switch terminals (**P46**) Fig. 7.
5. Solder 6 pin angled header to board (**P7**) for PiOLED (**P17**) attachment Fig. 7.
6. Solder female 6 pin plug header (**P8**) to board for power input from wiring harness (Fig. 8).
7. Solder Pololu power switch (**P9)** directly to board Fig. 8.
8. Solder screw terminals (**P11**) to picobuck driver (**P10**) (if not previously installed). Solder the complete picobuck to board, Fig. 9.
9. Solder Pro Trinket (**P12**) to board (Fig. 9) and upload Arduino code (**DF11**) to Trinket.
10. Solder current sensor (**P13,** Fig. 10), 5 V regulator (**P14,** Fig. 10), and real time clock (**P15**, Fig. 11) to board. Insert LiCB 3 V battery (**P16**) into the real time clock (Fig. 11).
11. Insert the PiOLED (**P17**) into 6 pin header (**P7**), Fig. 12.
12. Solder 40 pin female connector (**P18**) to PCB header Fig. 13, followed by insertion of Raspberry Pi 3B+ (**P19**) to base of attached 40 pin female header Fig. 13.
13. Attach a Female to Female jumper (**P20**) from I2C bus (**P5**) to GPIO2 (Pin# 3) and GPIO3 (Pin# 5) on Raspberry Pi 3B+ (**P19**) (Fig. 15).
14. If the female to female jumper is too long or obstructing board access, a small zip tie can be used to secure the jumper to the top of the real time clock (**P15**) and/or current sensor (**P13**) using the attachment points at the top corner of each board.
15. Camera and attached ribbon cable (**P22**) can be inserted into Raspberry Pi CSI port Fig. 16**,** following assembly of camera components and removal of IR filter (See sections 5.1.2 and 5.1.3.)
16. To operate the strobe system (assembly instructions in 5.1.4) a wiring harness must be connected to picoBuck LED driver (**P10**) to allow LED control. Using screw mount terminals (**P11**), connect 10–12 in. of wire (P25) to each terminal following the colored diagram shown in Fig. 17. Attach wires to tin crimp pins and insert into 6 circuit Molex plug (**P24**) in accordance with the diagram found in Fig. 18.
17. After uploading trigger_camera_disk.img (**DF10**) onto a 64 GB MicroSD card (**P51**), insert the card into the receptacle at the base of the Raspberry Pi.

### 5.2 Camera FR filter removal

Before the camera components can be used in tandem with an FR illumination system, the factory installed FR filter must be removed, for removal instructions beyond this manual refer to HQ camera filter removal .

1. Begin by cleaning the project area in an effort to minimize any particulates which may fall in the exposed camera sensor during filter removal.
2. Remove the CS-mount adapter attachment ring Fig. 20, followed by the ¼” tripod mount (requiring hex lock keys) which is unnecessary and will be permanently removed (Fig. 21).
3. Remove the two 1.5 mm hex lock keys from the base of the main circuit board and gently lift the lens mount to expose the IR filter (Fig. 21).
4. Using a sharp blade or fine tipped flathead screwdriver, carefully loosen the edges of the filter from the top of the Sony IMX477 sensor and remove the FR filter without breaking it (Fig. 22).
5. Reinstall the lens mount and replace the CS-mount adapter ring before attaching the wide angle lens (**P21**), refer to Fig. 23, Fig. 24.

### 5.3 Camera assembly

1. To create the port mount assembly for camera and LED attachment, insert a nut (**P26**) into the fitted hole in the center of **DF1**. Slide the resulting part beneath **DF2** and thread the bolt (P27) into its corresponding nut Fig. 25. By tightening this bolt
  (**P27**) the **DF1** wedge will spread the footprint of **DF2** allowing it to mount within a 3″ pipe without moving.
2. The camera mount attachment (**DF3**) will be mounted on top of the port mount and secured with bolts (**P28**), Fig. 26. Next the camera circuit board (**P23**) is installed above the camera mount (**DF3**) using bolts (**P29**), Fig. 26, Fig. 27.
3. Screw the CS-mount lens (**P21**) onto the Arducam base and insert the ribbon cable (**P22**) into the CSI/DSI connector, Fig. 27, Fig. 28.

### 5.4 LED assembly

1. Attach the LED support base (**DF4**) to the port mount (**DF2**) and secure with bolts (**P28**) (Fig. 29).
2. Secure the LED mounting plate (**DF5**) to the LED assembly base and secure with bolts (**P30**) (Fig. 29).
3. Install the wedge (**DF1**) used for expanding the port mount by inserting a nut (**P26**) into the wedge and threading the bolt (**P27**) through the port mount and into the nut (Fig. 30, Fig. 31).
4. Mount LED starboards (**P31, P32**) onto LED mounting plate (**DF5**) using screws (**P29**).
5. Solder wiring onto LED starboard terminals, run wire through port mount and out, to prevent wires from getting pinched during LED port mounting (Fig. 33). Install wire terminals in a female molex connector (**P24**) (Fig. 33, Fig. 34), for later connection to the male molex connector, attached to the strobe system seen in Fig. 19.

### 5.5 Battery and structural assembly

1. Battery pack (**P33**) wires are cut from the original 6 pin plug and mounted in a female 6 pin molex plug (**P34**) using tin crimp pins (**P35**) (Fig. 35). Yellow wires are combined into one crimp pin.
2. Threaded rods (**P36**) are fitted with nuts (**P37**) in order to mount battery holders in place, along the length of the rods (Fig. 36). After installing all the components along the length of the rods the tightening of the nuts will form a rigid structural design. To
  efficiently move nuts along the threaded rod a dremel with a soft polishing tip was used to spin nuts quickly along the length of the rods.
3. Two battery holders are mounted back to back (**DF7**), to support the middle of the battery (Fig. 37, Fig. 38).
4. Before installing the final battery mount end cap the wired battery pack is placed inside the battery holders before being fixed in place with the last mount (Fig. 38, Fig. 39), two more battery packs are installed in a similar fashion (Fig. 39).
5. Install mounts for the computer components (**DF8**), Fig. 40.
6. Mount the components using bolts and nuts (**P29**) placed according to the diagram in Fig. 41, Fig. 42.
7. Install the front bulkhead (**DF9**) by loosening all the nuts along the length of each threaded rod and use a powered drill to turn the rod through the bulkhead by tightening the drill head on the far end of the rod and slowly spinning it as it feeds into the mounting holes in the bulkhead (Fig. 43, Fig. 44).
8. A wiring harness to connect all three battery packs and provide power input (**P8**), can be fabricated by wiring (**P25**) 4 male molex connectors (**P38**) in parallel (Fig. 45, Fig. 46). The wiring harness clips to the sides of the battery holders (**DF6,7**) to prevent damage during loading and unloading from the housing. At each wire intersection along the wiring harness heat shrink tubing (**P53**) was used to maintain a strong waterproof connection.
9. After all components are in place and securely fastened trim off excess threaded rod length using an angle grinder.
10. Battery packs can be charged by wiring a battery charger (**P47**) to a male molex connector in the same configuration as the wiring harness, to allow individual charging of each battery. Female molex plugs (**P34, P35**) will be wired to each battery charger.

### 5.6 Housing construction

1. Cut schedule 80 PVC pipe (**P40**) into a 3ft length (using a table or band saw) and cap the end (**P41**), Fig. 47 using schedule 80 PVC cement (**P48**).
2. Attach a *t*-joint (**P42**) to the PVC pipe with glue followed by a short 6in piece of pipe (**P40**), Fig. 48. Taper the insides of the 6in pipe segment using a sander or dremel to allow for easier loading and unloading of the camera system.
3. Attach a second *t*-joint to the 6in pipe segment and insert a 6in pipe into each of the 3 available female receptacles (Fig. 49).
4. Prepare 3 union joints (**P43**) for attachment by removing the union joint sleeve and replacing it with a plexiglass plate (**P44**) for the loading and camera ports and a glass plate (**P45)** for the strobe port (Fig. 50, Fig. 51).
5. The plexiglass plate used to cover the loading port requires the installation of a Blue Robotics high pressure switch (**P46**). Using the appropriate drill bit, make a larger hole ¼” through the plexiglass in the center of the plate. Create a second smaller hole continuing through the plate which will allow a taping set to thread the hole (Fig. 52).
6. Install the Blue Robotics switch by removing the nut and wrapping the threads with plumbers tape. Using a wrench screw the switch into the hole and replace the washer (Fig. 53, Fig. 54).
7. The completed housing should match the visualization shown in Fig. 55.
8. After camera and LED assembly install port mounts in designated ports (Fig. 2) by tightening center bolts until snug. Load cameras with desiccant packs (**P50**) to prevent moisture from corroding electronics.

### 5.7 Tools needed

● *3d printer*
● *Table saw*
● *Band Saw*
● *Belt sander*
● *Dremel with diamond bit head*
● *Power drill and screw taping set for installation of BR switch*
  o *Recommended through hole diameter 10.2 mm*
  o *Bulkhead thread M10 × 1.5*
● *Molex connector crimping tool*
● *Soldering station*

## 6 Operation instructions

### 6.1 Software

OCTOPUS software is written in the Python programming language (Version 3.5.3) using only open-source libraries. For ease of use, the main operating software is separated into two python scripts. The first, triggercam_main.py, contains the main operating code while the second, triggercam_functions.py, contains accessory functions required during runtime. The following table lists the additional required libraries (see Table 3):

**Table 3:** Software library.

Library	Description	Source
adafruit-circuitpython-ina219	Library for the INA219 voltage and current monitoring integrated circuit by adafruit	https://github.com/adafruit
adafruit_blinka	Support for i2c communication with adafruit products using CircuitPython	https://github.com/adafruit
adafruit_ssd1306	Library for operating the adafruit PiOLED − 128x32 miniature screen	https://github.com/adafruit
Pillow (Python Imaging Library)	Required for operating the miniature screen	https://pypi.org/project/Pillow/
PyOpenCV (Version 3.4)	Required for all image analysis operations	https://pypi.org/project/pyopencv/

### 6.2 Configuration

The main operating parameters are specified in the configuration file “settings.cfg”, which includes general program settings, image acquisition settings, and triggering thresholds. The table below lists all configurable parameters and default values (see Table 4).

**Table 4:** Operating settings.

Section	Parameter	Default	Data type	Valid options	Description
general_settings	collection_type	trigger_using_red	string	still_intervalometer, trigger_using_red, trigger_using_ambient	Main collection mode – intervalometer is a simple set of timed images, trigger modes rely either on red strobe or ambient light for illuminating scene for evaluation target presence
	system_id	pi_triggercam_001	string	user specified	Name of the camera system to be used for metadata (if several systems are used)
	initial_wait	1	integer	unlimited	Rest period before system starts acquisition. Can be used if deployment takes a while.
	low_voltage_cutoff	11	floating point	unlimited	Voltage value for shutting the system down, typically ∼11 V for 12 V battery systems
	shutdown_at_end	false	boolean	True/False	Flag for software power down pi at end of collection – false can be used for bench testing.
	shutdown_wifi_on_collection	true	boolean	True/False	Allows system to turn off wifi when collection starts – this can save battery life, wifi is available during initial wait to allow interaction for downloading or updates, etc.

intervalometer_settings	max_images	10	integer	unlimited	Allows for a fixed collection period
	image_interval	1	floating point	unlimited	Seconds between shots by intervalometer
	strobe_channel	red	string	red and white(UV in current implementation)	Strobe color, two possibilities – white and red

motion_detection_settings	motion_detect_interval	1	integer	unlimited	Seconds between evaluating for motion
	post_detection_rest	5	integer	unlimited	Minutes before starting motion sense again
	max_runtime	60	integer	unlimited	Can be used to limit trigger evaluation period
	foreground_threshold	8	integer	greyscale value (0–255)	Grayscale value for target detection threshold (MOG foreground mask)
	trigger_ROI	[20, 20, 280, 200]	integer	left bottom corner and width, height based on a 320X240 img	Only required if triggering is to be excluded from certain portions of the image – only targets inside this box will be evaluated, even if object is part way in
	frame_history	25	integer	20–100	Specifies how many frames to keep for background modeling.
	trigger_eval_method	object_size	string	object_size or pixel_total	Triggering mode: “object_size” method uses connected components to evaluate above threshold objects in image, and threshold is then used to decide if sufficient change has occurred to collect image. If “pixel_total” is used, all above threshold pixels are considered regardless of connected components.
	min_object_size	500	integer	unlimited	Size of object (connected components above threshold) to trigger (small objects ignored) – only relevant for “object_size” trigger mode
	min_pixel_count	10,000	integer	unlimited	Setting for how many pixels above threshold constitute a trigger event, valid only with “pixel_total” trigger mode

camera_configuration	image_resolution	M	string	L = (1024,768) ∼0.8 Mp, M = (2048,1520) ∼3 Mp H = (4056,3040) ∼12 Mp, default value is M	Image resolution for triggered images, specific to the Raspberry Pi HQ Camera
	auto_contrast	off	boolean	off/on- default setting is “off”	Flag to enable use of OpenCV clahe adaptive contrast on image to enhance performance
	exposure	0	integer	unlimited	Exposure duration in microseconds, if 0 then auto expose
	iso	800	integer	100, 200, 400, 800, 1600	Camera gain – higher values are more light sensitive but poorer quality (graininess)
	strobe	envelope	string	off, camera, envelope	Strobe mode – if not set to “off”, strobe signal can originate from the camera itself (“camera” – only works with certain camera settings), typically a double flash (the first to meter scene, then second for image exposure), or “envelope” where strobe is independently triggered around exposure envelope
	pre_strobe_fire	0.1	floating point	0–0.5	Value for how far ahead of starting image capture to turn on the strobe, only relevant for “envelope” mode
	strobe_duration	0.5	floating point	0.5–2	Maximum in seconds of how long to leave strobe light on – LED strobes can overheat if left on form more than a few seconds
	image_depth	monochrome	string	color, monochrome	Image depth – color image is 24 bit (8 *×* 3 color channels), monochrome is single 8 bit channel
	image_type	JPEG	string	JPEG, PNG, BMP	Common file formats available for opencv.
	image_quality	95	integer	70–100	Compression level for jpeg 70–100, default 95. If PNG format is used, than values are 0–9 with a default of 3

### 6.3 General operation

Each power up cycle is considered a “deployment”. The system is powered up by turning the Blue Robotics underwater switch knob (**P46**) to the “on” position (see Fig. 54). The system will then boot up the raspberry pi and automatically start the main program script using the command line call “python3 triggercam_main.py”. This program reads the “setup.cfg” file and stores the settings in a python dictionary to guide the main program execution. The initial action of the program is to display the IP address of the Wi-Fi interface for the raspberry pi, the system voltage and free disk space for 30 s. The program will then count down for the pre-determined number of minutes (as set by the user in the settings) before starting acquisition. Each new deployment is given a name based on the time using the format DMMDDYYYY-Thhmmss. A set of folders is created for storing images and log files related to each deployment, as well as a copy of the configuration file “settings.cfg” for reference. The voltage level is recorded in the deployment log file whenever an image is taken, enabling the voltage draw profile to be evaluated.

When conducting field deployments several factors should be accounted for to promote high quality pictures and prevent system failure. O-rings should be covered in a light coat of silicone-based vaccume grease (**P49**). All surfaces should be cleaned with alcohol and microfiber cloth prior to greasing to prevent contaminants ruining the waterproof seal. After assembling the system O-rings must form an smooth seal against the plexiglass without any visible bubbles or cataminants. Dessicant packets should be loaded with the system to prevent condensation on viewports and aid in maintaining system integrity. When deploying; systems weigh approximately 13 kg at the surface and are negatively boyant when submerged. This must be accounted for if manually deploying using SCUBA. Sidemounting cameras using boltsnaps proved effective but the use of liftbags would be recommended if more than a few systems are being deployed or housing size is increased to accomodate more batteries. Camera systems can be mounted in a number of orientations, however facing the seafloor proved most effective at limiting uneccesary triggering events and observing benthic species. Camera and LED ports act as boyant portions of the housing and tend to turn the ports upright, thus cameras should be secured to maintain the frame on the desired subject, zipties or bungeechords proved effective for this issue. When collecting camera systems avoid opening housings at dive site to prevent damage during transport, keep seals clean, and to prevent saltwater from entering housing. Before opening housing, dunk entire housing in clean freshwater to rinse off any saltwater, allow the housing to dry before opening for charging, data removal and maintence.

### 6.4 Operation modes

**Fig. 56:**
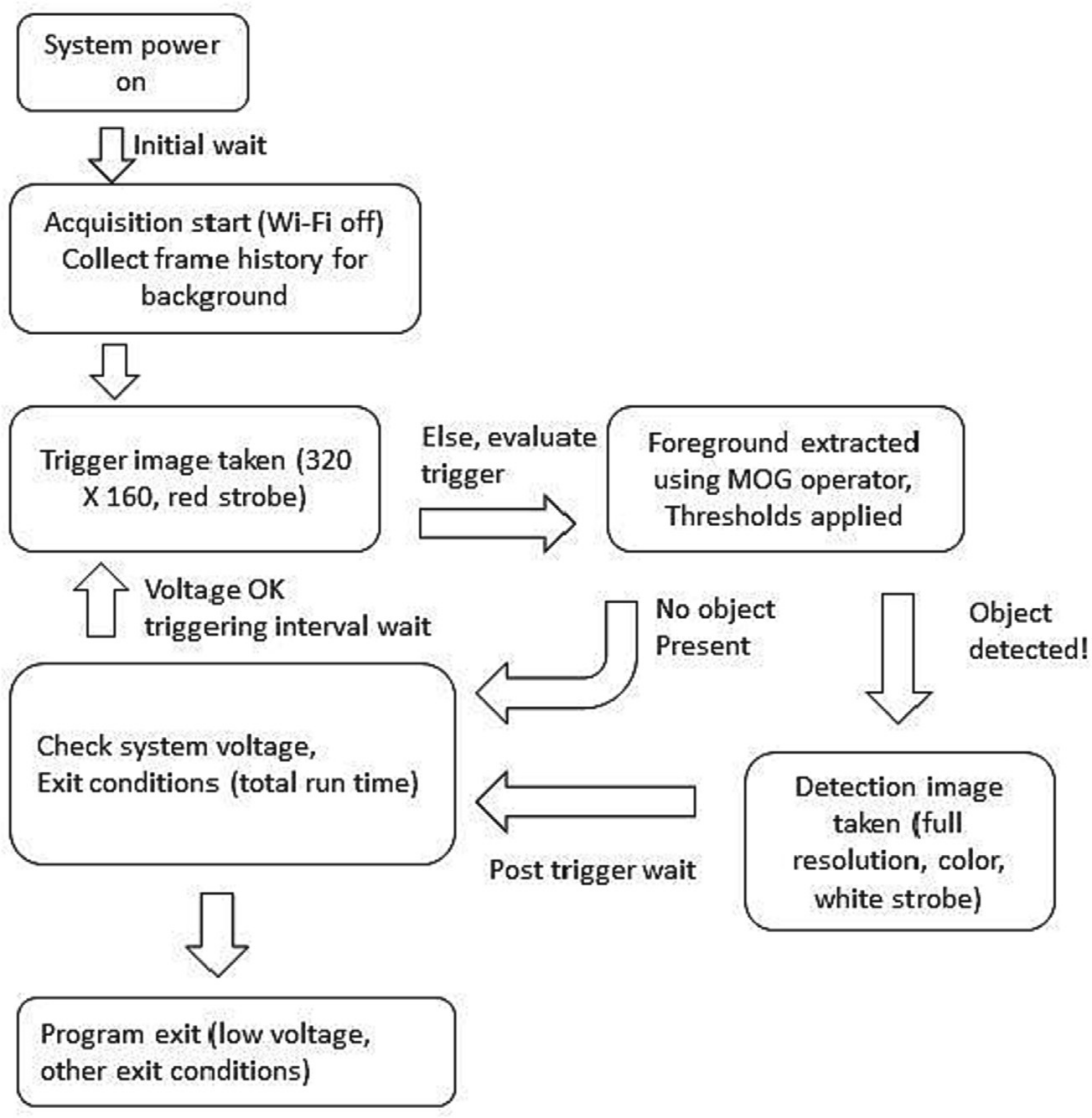
Flow diagram of triggering process starting with power up and ending with termination of the program due to low power conditions.

In intervalometer mode, a single still image is taken at specified time intervals using preconfigured image settings and strobe channel up to a specified maximum number of images or until other exit conditions are detected, such as memory limitation for data writing or low voltage. In triggered mode, the camera takes a low resolution image (trigger evaluation image – TEI) using unobtrusive red light strobe or ambient light at specified intervals to evaluate the scene for change, such as the arrival of a target. The triggering process is illustrated graphically in Fig. 56. When sufficient change is detected, a full resolution image is taken using the desired strobe channel for proper illumination. The TEI sequence is also stored to allow post-deployment evaluation of trigger performance and adjustment of trigger sensitivity.

### 6.5 Triggered operation details

The trigger operation relies on motion detection by using a background subtraction algorithm termed Mixture-of-Gausians (MOG; [9]). With OCTOPUS, a background model is constructed at the start of data collection from a series of TEI images (320 × 240 monochrome) of the scene background. TEI images can be illuminated using a lower detectability strobe such as red (600-nm) which is less detectable by many marine organisms, or collected with ambient lighting. After the TEI images are captured, a low pass filter is applied (Gaussian Blur) with kernel size 5x5 pixels, and the image is cropped to the boundaries specified in the region-of-interest (ROI) parameter. The low pass filtering eliminates noise and produces a more stable background, and the ROI allows for a focus area for motion detection to be specified, for example, away from the image edges. After the background model is established, each successive frame is evaluated for differences relative to the background (or image “foreground”) based on an initial sensitivity threshold (in OCTOPUS configuration this is *foreground_threshold* parameter). The background model is continually updated as images are collected. Once the foreground is extracted as a binary mask image, it is subjected to a second level of scrutiny by using one of two approaches; 1) total pixel level – this is simply a count of non-zero pixels in the mask, or 2) by considering object size, where the foreground mask is evaluated using a connected components step, and looking for objects that meet a minimum pixel area. Before applying the connected components step, a dilation and erosion morphological operator is sequentially applied to the foreground mask to merge fragmented foreground objects that are likely a single target. If the total number of foreground pixels exceeds the min_pixel_count (approach 1), or any of the objects in the scene exceed the *min_object_size* parameter value (approach 2), conditions for a trigger event have been met. Capturing a triggered image consists of resetting camera resolution to the specified desired resolution (*image_resolution* parameter), and if desired, apply contrast enhancement using the Contrast Limited Adaptive Histogram Equalization (CLAHE) implementation in OpenCV. A secondary strobe channel can be specified for this operation, for example a white, full spectrum, strobe for capturing the true color properties of the target. The full resolution image is then written to disk.

### 6.6 Image downloading and configuration editing

The Raspberry Pi computer is set up to automatically connect to a specified wireless network, if available. This needs to be set up during the initial system build, with a user specified network. To download images collected during a deployment, the system must be powered on within range of the wifi router. Alternatively, the front port can be opened and an ethernet cable used to make a hardwired connection to a router. Then an FTP program such as WinSCP or FileZilla can be used to connect to the Raspberry Pi and transfer the files to another computer. The “settings.cfg” file can also be accessed at this time by directly editing the file using an SSH client or by copying the file to a separate computer, editing and then replacing it on the Raspberry Pi.

## 7 Validation and characterization

### 7.1 System performance

We tested the performance of this camera system in one of the most challenging situations for an aquatic camera trap: tracking the movements of a highly mobile animal (octopuses) in a high-current location. Four camera systems were built and tested at Rosario Beach Marine Laboratory followed by a series of field deployments at Driftwood Park, in Island County, Washington state to monitor ruby octopuses (*Octopus rubescens*) at den sites [10]. Driftwood Park is located on Admiralty Inlet, one of only two inlets to the Puget Sound, Washington, USA, and therefore experiences significant and frequent tidal currents. Cameras were mounted in deployment frames and placed above occupied octopus’ dens. In total, 16 deployments were performed which amassed over 785 h of data collection (nearly 33 days). During these deployments the systems were able to record 46 individual octopuses, with 15,972 min (266 h) of octopuses present beneath the cameras in addition to 1,485 instances of other macrofauna species. A total of 249,163 triggered images were captured, which likely represents a relatively high value of triggered images for the deployment time due to the high current nature of the deployment site. This is opposed to 1.4 million images that would have been captured by a 2 s interval time-lapse system that would be need to capture data at a similar temporal resolution as this device. By utilizing the motion activated feature of the camera, octopus were monitored continuously both day and night. The UV strobe system incorporated into the camera was used to distinguish individual octopus by fluorescing subdermal tags, however this function could be easily replaced with a different lighting system adapted to the researchers needs. System settings for these field deployments were configured to utilize auto exposure without auto contrast, an ISO of 800, image quality of 95 and monochrome image depth. The data collected led to insights into the diel activity cycles of this octopus species and revealed new inter-species interactions, specifically with kelp greenlings, examples of these interactions can be found following the attached link [10].

### 7.2 Capabilities of the system

• Maximum deployment duration ∼72 h.
• Can detect motion and capture images in any lighting condition.
• Hardware and code are very customizable.
• Can withstand depths of ∼800ft.
• Wireless access and configuration.
• Easily adapted lighting systems.
• Motion detection sensitivity can be adjusted to limit excess stimuli.
• Far Red lighting limits disturbance to animals.

### 7.3 Limitations of the system

• While much cheaper than comparable systems, this is not a budget system at ∼$1000 per system.
• Electronic assembly and Raspberry Pi flashing ability necessary.
• Bulky and heavy at ∼30 lbs dry weight.

### CRediT authorship contribution statement

**Jefferson W. Humbert:** Conceptualization, Methodology, Validation, Investigation, Data curation, Visualization, Project administration. **Kirt L. Onthankv:** Formal analysis, Investigation, Resources, Data curation, Supervision, Funding acquisition. **Kresimir Williams:** Software, Resources, Supervision.

## Declaration of Competing Interest

The authors declare that they have no known competing financial interests or personal relationships that could have appeared to influence the work reported in this paper.
